# Chemical richness and diversity of uncultivated ‘Entotheonella’ symbionts in marine sponges

**DOI:** 10.1038/s41589-025-02066-0

**Published:** 2025-11-13

**Authors:** Maria Dell, Masato Kogawa, Alena B. Streiff, Taro Shiraishi, Alessandro Lotti, Christoph M. Meier, Michelle A. Schorn, Christopher Field, Jackson K. B. Cahn, Hiromi Yokoyama, Yuito Yamada, Eike Peters, Yoko Egami, Yu Nakashima, Karen Co Tan, Christian Rückert, Mohammad Alanjary, Jörn Kalinowski, Tomohisa Kuzuyama, Paco Cardenas, Shirley Pomponi, Detmer Sipkema, Amy Wright, Kentaro Takada, Ikuro Abe, Toshiyuki Wakimoto, Haruko Takeyama, Jörn Piel

**Affiliations:** 1 Institute of Microbiology, Eidgenössische Technische Hochschule Zürich (ETH), Zurich, Switzerland; 2 Department of Life Science and Medical Bioscience, Waseda University, Tokyo, Japan; 3 Computational Bio Big-Data Open Innovational Laboratory, AIST-Waseda University, Tokyo, Japan; 4 Research Organization for Nano & Life Innovation, Waseda University, Tokyo, Japan; 5 Graduate School of Agricultural and Life Sciences, The University of Tokyo, Tokyo, Japan; 6 Collaborative Research Institute for Innovative Microbiology, The University of Tokyo, Tokyo, Japan; 7 Laboratory of Microbiology, Wageningen University and Research, Wageningen, The Netherlands; 8 Laboratory of Natural Product Chemistry, Faculty of Pharmaceutical Sciences, Hokkaido University, Sapporo, Japan; 9 Graduate School of Pharmaceutical Sciences, The University of Tokyo, Tokyo, Japan; 10 Center for Biotechnology (CeBiTec), Bielefeld University, Bielefeld, Germany; 11 Bioinformatics Group, Wageningen University and Research, Wageningen, The Netherlands; 12 Pharmacognosy, Department of Pharmaceutical Biosciences, BioMedical Center, Uppsala University, Uppsala, Sweden; 13 Museum of Evolution, Uppsala University, Uppsala, Sweden; 14 Bioprocess Engineering, Wageningen University and Research, Wageningen, The Netherlands; 15 Harbor Branch Oceanographic Institute, Florida Atlantic University, Fort Pierce, FL USA; 16 School of Marine Biosciences, Kitasato University, Sagamihara, Kanagawa Japan

**Keywords:** Bacteria, Biosynthesis, Natural products

## Abstract

Marine sponges are the source of numerous bioactive natural products that serve as chemical defenses and provide pharmaceutical leads for drug development. For some of the compounds, symbiotic bacteria have been established as the actual producers. Among the known sponge symbionts, ‘*Candidatus* Entotheonella’ members stand out because of their abundant and variable biosynthetic gene clusters (BGCs). Here, to obtain broader insights into this producer taxon, we conduct a comparative analysis on eight sponges through metagenomic and single-bacterial sequencing and biochemical studies. The data suggest sets of biosynthetic genes that are largely unique in 14 ‘Entotheonella’ candidate species and a member of a sister lineage named ‘*Candidatus* Proxinella’. Four biosynthetic loci were linked in silico or experimentally to cytotoxins, antibiotics and the terpene cembrene A from corals. The results support widespread and diverse bacterial roles in the chemistry of sponges and aid the development of sustainable production methods for sponge-derived therapeutics.

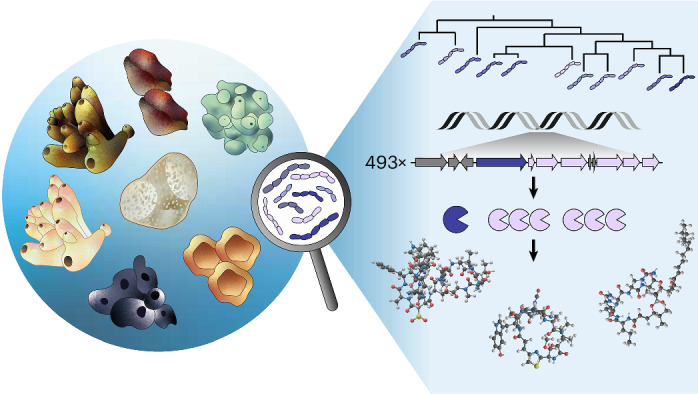

## Abstract (web summary)

Marine sponges host bacteria that produce diverse bioactive compounds. Here, the authors conduct a large-scale metagenomic, single-bacterial and biochemical study to reveal the untapped biosynthetic potential of ‘Entotheonella’ symbionts, a talented producer taxon from microbial dark matter.

## Main

**Fig. 1:**
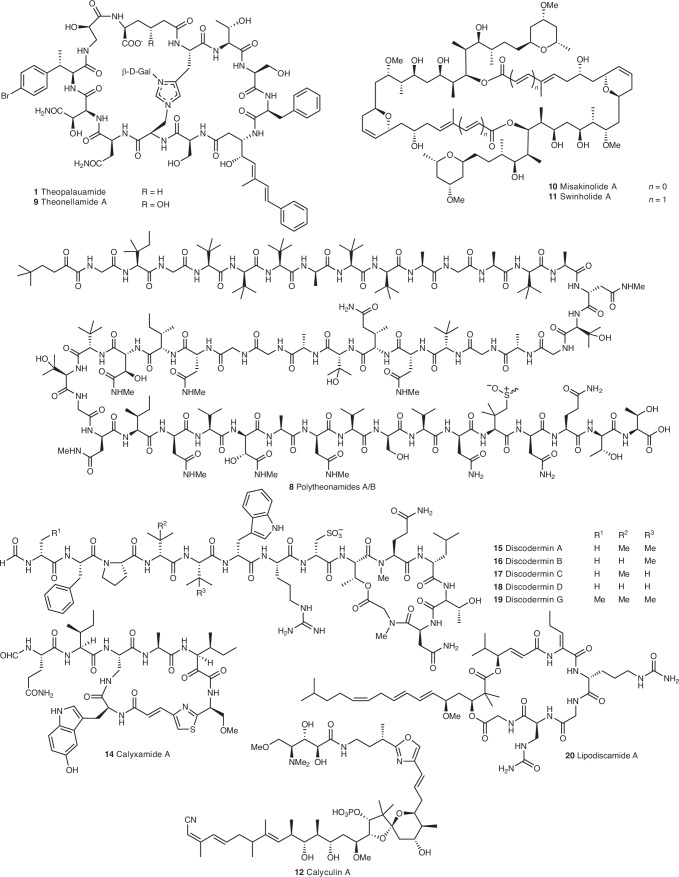
Sponge-derived NPs relevant to this study (selection). A full overview of numbered compounds is provided in Supplementary Fig. 1.

Sponges (Porifera) are ancient metazoans with unusually diverse bioactive natural products (NPs)^1,2^ with suspected or demonstrated roles as chemical defenses against grazers or epibionts^3–8^. In addition, sponge NPs are a rich resource for drug development, with spongouridine and halichondrins as examples of important leads in antiviral and cancer therapy^9–11^. While some sponge NPs are host synthesized^12,13^, evidence increases that many others are products of the sponge microbiome^14–17^. Studying these often diverse bacterial communities remains challenging, as most members resist cultivation, making functional characterization difficult^18^. Bacterial origins have been established for several sponge NPs, including the anticancer drug candidates psymberin^19^, peloruside A^20,21^ and renieramycin^22^. The most prolific known sponge symbionts belong to the candidate genus ‘Entotheonella’ (quote format refers to uncultivated status), first reported by Bewley, Schmidt, Haygood, Faulkner and coworkers from a Palauan *Theonella* *swinhoei* sponge^23–26^. *T*. *swinhoei* displays remarkable chemical diversity across distinct chemotypes^24,27,28^. In the Palauan variant, chemical analysis localized theopalauamide (**1**) (Fig. 1) to a cell fraction enriched in filamentous bacteria^26^ named ‘*Candidatus* Entotheonella palauensis’, suggesting it as the producer^23^. Further genomic and biosynthetic work identified ‘Entotheonella’ producers in other *T*. *swinhoei* chemotypes, assigning them to four candidate species^29–31^.

These four symbionts, members of the candidate phylum ‘Tectomicrobia’ (‘Entotheonellaeota’), feature unusually large ~10-Mb genomes with diverse biosynthetic gene clusters (BGCs) for known sponge compounds and predicted unknown NPs. In the chemically rich yellow *T*. *swinhoei* chemotype (Y) from Japan, ‘*Candidatus* Entotheonella factor’ produces most known polyketides and peptides, including **2**–**8** (Table 1, Fig. 1 and Supplementary Fig. 1)^29,32,33^, while the coinhabiting ‘*Candidatus* Entotheonella gemina’ contains only orphan BGCs^29^. Recently, ‘*Candidatus* Entotheonella arcus’ was found colonizing some yellow *T*. *swinhoei* specimens^31^. In contrast, white *T*. *swinhoei* chemotypes (W) from Japan and Israel contain ‘*Candidatus* Entotheonella serta’, producing compounds such as **9**–**11**, in addition to containing many orphan BGCs (Table 1 and Fig. 1)^30,34,35^.

**Table 1:** Genome-sequenced ‘Entotheonella’ phylotypes and their host sponges analyzed in previous work or the current study (specified in column 1). Further details can be found in Supplementary Table 1

Sponge (suborder)	Known compounds (numbers in parentheses refer to representative structures in Fig. 1 and Supplementary Fig. 1)	Symbiont variants (E. refers to ‘Entotheonella’, P. to ‘Proxinella’)
*T*. *swinhoei* Y (Astrophorina); previous studies	Konbamide (**2**), keramamides (**3**), nazumamide A (**4**), pseudotheonamides (**5**), cyclotheonamide (**6**), onnamides and theopederins (**7**), polytheonamides (**8**), aurantosides and sterols	‘*Ca*. E. factor’ TSY1 ‘*Ca*. E. gemina’ TSY2 ‘*Ca*. E. arcus’ YB-1
*T*. *swinhoei* YB (Astrophorina); this study	See above	‘*Ca*. E. factor’ TSYB1 ‘*Ca*. E. gemina’ TSYB2 ‘*Ca*. E. mitsugo’ TSYB3
*T. swinhoei* WA (Astrophorina); previous study	Theonellamides (**9**) and misakinolides (**10**)	‘*Ca*. E. serta’ TSWA1
*T. swinhoei* WB (Astrophorina); this study	Theonellamides (**9**) and swinholides (**11**)	‘*Ca*. E. serta’ TSWB1 ‘*Ca*. E. consors’ TSWB2
*Theonella* sp. 1 BA (Astrophorina); this study	Unknown	‘*Ca*. E. melakyensis’ TCBA1 ‘*Ca*. E. serta’ TCBA2
*Theonella* sp. 2 BT (Astrophorina); this study	Unknown	‘*Ca*. E. symbiotica’ BT01 ‘*Ca*. E. inquilina’ BT02 ‘*Ca*. E. melakyensis’ BT03 ‘*Ca*. E. catenata’ BT04
*D. calyx* (Astrophorina); this study	Calyculins (**12**), kasumigamide (**13**), calyxamides (**14**), indole derivatives and cyclodipeptides	‘*Ca*. E. armillaria’ DC1
*D. kiiensis* (Astrophorina); this study	Discodermins (**15**–**19**), lipodiscamides (**20**)	‘*Ca*. E. monilis’ DK1
*D. dissoluta* (Astrophorina); this study	Discodermolides	‘*Ca*. E. tacita’ DD1 ‘*Ca*. E. baccata’ DD2 ‘*Ca*. E. tertia’ DD3
*A. cribrophora* (Spirophorina); this study	Unknown	‘*Ca*. P. opulenta’ AC1

Research on other sponge microbiomes revealed BGCs assigned to ‘Entotheonella’ by in situ hybridization. These encode the biosynthesis of calyculins (**12**)^36^ and kasumigamides (**13**)^37^ from *Discodermia* *calyx* and psymberin from a *Psammocinia* sp. sponge^38^. However, their metabolic diversity and phylogenetic affiliation remained unknown without further genomic data. A 16S ribosomal RNA (rRNA) gene-based study suggested ‘Entotheonella’ as a diverse lineage with numerous members primarily in sponges but also detected in sediments and soil^39^. These data and the high BGC diversity in few genome-sequenced representatives suggest a major untapped NP resource.

Here, we perform metagenomic, single-bacterial and functional studies to investigate these uncultivated organisms more broadly, particularly evaluating whether chemical richness is a general feature of this taxon. We interrogated how this feature distributes among taxon members and whether additional bioactive compounds are produced by these symbionts. Our data cover 15 candidate species across eight sponge chemotypes, assigned to 14 ‘Entotheonella’ phylotypes and an unexpected BGC-rich sister candidate genus. Results indicate high BGC diversity among ‘Entotheonella’ phylotypes with biochemically supported roles in producing both known and orphan sponge metabolites. This widespread chemical richness provides a foundation for targeted NP discovery from microbial dark matter.

## Results

### Selection of sponges for sequencing

**Fig. 2:**
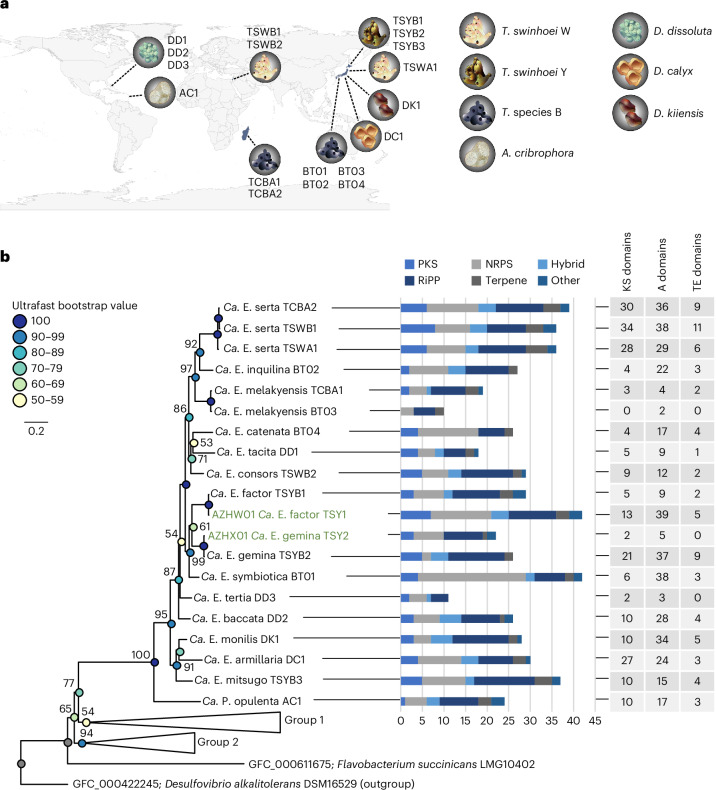
Distribution, phylogenetic relationships and metabolic potential of the analyzed sponge symbionts. **a**, Geographic origin of the analyzed sponges. Sponges are shown as idealized icons explained on the right. On the map, the labels besides the icons refer to the identifiers of symbionts identified in the sponges. **b**, Whole-genome phylogram generated with autoMLST of analyzed symbiont genomes and the two already published symbionts ‘*Ca*. E. factor’ TSY1 (AZHW01) and ‘*Ca*. E. gemina’ TSY2 (AZHX01), with the two latter labeled in green. Colored circles on nodes represent Ultrafast Bootstrap values. The composition of collapsed clades group 1 and group 2 is provided in the Supplementary Information. For each branch, the bar chart provides numbers of BGCs or BGC fragments identified by antiSMASH. The BGC classes used by antiSMASH were implemented here, with minor classes being combined as ‘other’. Hybrid refers to BGC loci combining features of several classes (PKS–NRPS, NRPS–RiPPs, etc.). The rightmost table shows total counts of core domains in PKS (ketosynthase (KS) domains) and NRPS (A domains) systems, as well as termination TE domains for both systems, per genome. Domain numbers might be underrepresented for genomes with lower coverage (for example, BT02, BT04 and DD3; <70%) or overrepresented for genomes with higher contamination (for example, TSYB1, TSWA1, TCBA1 and DD1; >10%). More detailed genome statistics are provided in Supplementary Table 3.

We initiated our study by selecting a taxonomically and geographically diverse set of ‘Entotheonella’-containing sponges (Table 1). These comprised Japanese *Discodermia* *kiiensis*^29,39^, previously identified as a source for discodermin antibiotics^36^ and lipodiscamide cytotoxins^40,41^, *D*. *calyx*^36^, harboring the cytotoxic calyculins (for example, **12**)^42^ and calyxamides (for example, **14**), and *Discodermia* *dissoluta*^43^ from the Bahamas, containing the anticancer discodermolides^44,45^. All sponges were known to contain ‘Entotheonella’^29,36,39,43^ but lacked genome sequences. In addition, microscopy revealed symbionts with an ‘Entotheonella’-like morphology in two unidentified *Theonella* specimens with a new, blue phenotype from Japan and the Mozambique Channel. Furthermore, ‘Entotheonella’-like DNA contigs were detected in a sequenced *Aciculites* *cribrophora* metagenome. These three sponges with uncharacterized chemistry were also included in our study. Lastly, we reassessed the previously analyzed^39^ chemically complex Japanese *T*. *swinhoei* Y with new assembly methods to generate improved ‘Entotheonella’ genomes. For this purpose, a further specimen of this chemotype, named *T*. *swinhoei* Y2, was collected. In total, the analyzed sponges encompassed eight specimens collected at seven locations and belonging to two suborders, at least six species and eight chemotypes (Fig. 2a).

### Identification of 14 ‘Entotheonella’ phylotypes

We previously observed that some ‘Entotheonella’ variants resisted metagenomic sequencing but were amenable to single-bacterial sequencing^30^. We, therefore, used either metagenomic or single-filament sequencing or both (Supplementary Tables 2 and 3), depending on method success. Metagenomic sequencing was used for *D*. *dissoluta* and *Theonella* sp. 1 BA after mechanical enrichment of filamentous bacteria. *A*. *cribrophora* underwent full metagenome sequencing with subsequent binning. For remaining sponges, single-bacterial sequencing was performed in addition to or instead of metagenomics using cell separation, microdroplet encapsulation, microscopy-aided sorting and genome amplification^46^. This procedure proved valuable for *D*. *calyx*, where multiple metagenomic attempts failed or yielded poor genome coverage^46^. Single-bacterial sequencing was also applied when plasmids or multiple ‘Entotheonella’ phylotypes per sponge were detected, as in *T*. *swinhoei* Y containing two ‘Entotheonella’ symbionts and one or more plasmids^29^.

Assembly quality assessed using CheckM^47^ indicated ~13% to >90% genome completeness (Supplementary Table 3). The most complete genome was obtained for ‘E. serta’ from *T*. *swinhoei* WB (95.7% completeness, 7.86% contamination), while the lowest-quality dataset was a metagenome-assembled genome (MAG) of ‘Entotheonella tertia’ from *D*. *dissoluta* (12.9% completeness, 0.0% contamination, 2.13-Mbp assembly size). Estimated genome sizes ranged from 5.36 to 16.54 Mbp (Supplementary Table 3), with high-quality values around 9 Mbp. Except for ‘Poriflexus aureus’ (~14 Mbp), previously identified in two *Theonella* sponges^46^, ‘Entotheonella’ members feature, on the basis of a previous large-scale study^48^, some of the largest genomes identified among sponge symbionts. Phylogenomic relationships were studied using FastANI^49^ and autoMLST^50^. According to binning, single-bacterial sequencing and phylogenomic data (Fig. 2b), sponges contained one to four ‘Entotheonella’ phylotypes representing different candidate species. Additionally, we identified an *A*. *cribrophora* symbiont initially suggested by GTDB-Tk^51^ to belong to ‘Entotheonellaceae’ (Supplementary Information). Deeper analysis using average nucleotide identity (ANI), multilocus sequence typing (MLST) and 16S rRNA gene sequences supported its affiliation with a distinct tectomicrobial candidate genus (Supplementary Fig. 2). We included this organism, named ‘Proxinella opulenta’ AC1, in the current study because of its ‘Entotheonella’-like BGC richness, as discussed below. This contrasts with the reported genomes from the tectomicrobial genus ‘Bathynella’ with low BGC numbers^39^.

Among all analyzed sponges, we identified 14 distinct ‘Entotheonella’ variants outside of ‘P. opulenta’ AC1, with proposed names in Table 1. Different sponges mostly harbored distinct ‘Entotheonella’ phylotypes, consistent with previous 16S rRNA gene-based observations^39^, but some closely related symbionts appeared in different sponge species, suggesting horizontal transfer or inheritance from a common ancestor. For example, ‘E. serta’ was identified in *T*. *swinhoei* WA and WB and in *Theonella* sp. 1 BA from three different locations. ‘E. melakyensis’ was found in the blue *Theonella* sp. sponges from Japan and the Mozambique Channel. Despite ANI values slightly below the species cutoff (93.63%; Supplementary Fig. 3), multilocus phylogeny suggests the same candidate species (Fig. 2b). Symbiont variability also existed among specimens of the same host type; *T*. *swinhoei* Y1 and Y2 both contain ‘E. factor’ and ‘E. gemina’, while only Y2 additionally harbors a third ‘Entotheonella’ phylotype, ‘E. mitsugo’. Similarly, ‘E. serta’ was the sole variant in *T*. *swinhoei* WA but accompanied by ‘E. consors’ in *T*. *swinhoei* WB. These results reveal complex symbiont coevolution and horizontal acquisition patterns with likely consequences for sponge chemistry. Comparing our 14 ‘Entotheonella’ variants to ‘E. arcus’^31^ and ‘E. halido’^52^ reported during the completion of our study using FastANI (Supplementary Fig. 4) confirmed them as distinct candidate species. By uncovering 11 additional ‘Entotheonella’ and one ‘Proxinella’ phylotypes, we expanded knowledge beyond *T*. *swinhoei* sponges and identified ‘E. mitsugo’ as yet another phylotype in addition to ‘E. factor’, ‘E. gemina’ and ‘E. arcus’ in some yellow specimens of *T. swinhoei*.

We analyzed 16S rRNA gene sequences for comparison to the whole-genome tree (Fig. 2b). Using ssu-finder in CheckM^47^, we identified 27 16S rRNA genes or fragments in all genomes, except for ‘E. melakyensis’ TCBA1, ‘E. serta’ TCBA2 and ‘E. tertia’ DD3. Four sequences were excluded as chimeric or duplicated. Of the remaining 23, eight were of sufficient length for phylogenetic analysis. The 16S rRNA phylogram largely mirrored the whole-genome phylogeny (Supplementary Fig. 5a). To relate our variants to the theopalauamide-containing, unsequenced ‘E. palauensis’ from Palauan *T*. *swinhoei*^23,24^, we compared the reported four 16S rRNA gene sequences from this sponge to our data. Only one (AF130847) exceeded 1,300 nt and was included in the phylogram (Supplementary Fig. 5b), which suggested a distinct phylotype. Further alignment including our shorter sequences (Supplementary Fig. 6a) and all four ‘E. palauensis’ 16S rRNA genes showed pairwise identities around 97% (Supplementary Fig. 6b), indicating no close relationship with any of our phylotypes despite the previous finding that the Palauan *T*. *swinhoei* contains NPs similar to those assigned to ‘E. serta’ in *T. swinhoei* WA and WB^23,24^.

### Few shared gene clusters across BGC-rich symbionts

**Fig. 3:**
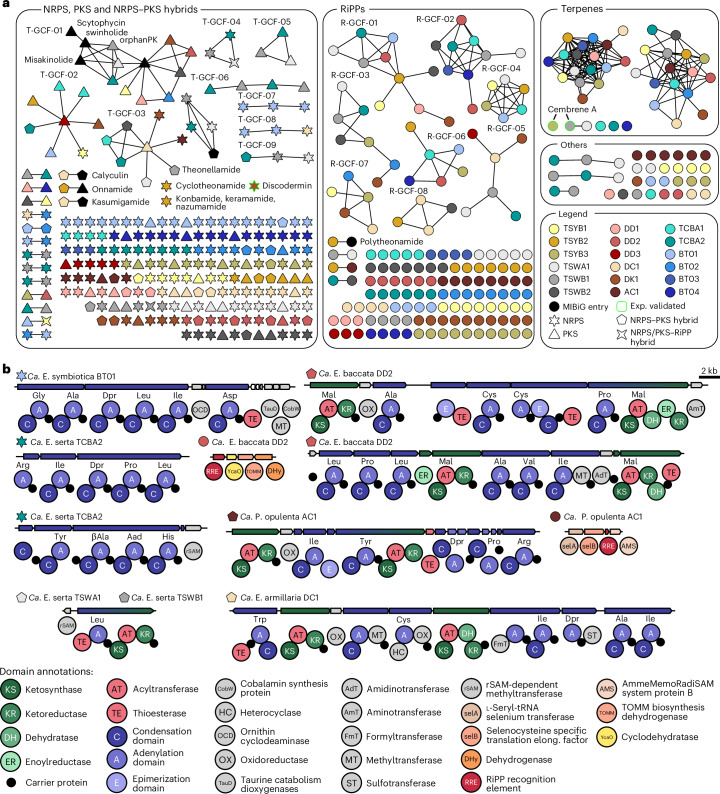
Biosynthetic potential analysis of all genomes. **a**, BiG-SCAPE output of a BGC network divided into the major NP classes: thiotemplate, RiPPs, terpenes and others. If two shapes are connected, their BGC (fragments) are similar to each other. The various colors and shapes refer to the genome and thiotemplate enzyme family, respectively, as described in the legend. **b**, Selected BGCs and their encoded biosynthetic domains are shown. For acyltransferase (AT) and A domains, the substrate selectivity as predicted by antiSMASH is written above the domain. The protein family of domains is provided in the domain annotations. Dpr: 2,3-diaminopropionic acid.

To evaluate the biosynthetic potential of the symbiont genomes, we searched for BGCs using antiSMASH^53^ followed by manual reanalysis for validation and detection of orphan biosynthetic loci. Genomes contained a consistently high number of BGCs (fragments) that ranged from 10 to 42 (Fig. 2b and Supplementary Table 4). In fragmented genome sequences, the BGC numbers for large, multimodular polyketide synthases (PKSs) or nonribosomal peptide synthetases (NRPSs) are overrepresented when BGCs are distributed over multiple contigs. To allow for a better comparison, Fig. 2b also shows catalytic domain counts including terminal domains for such multimodular assembly lines. For information about the chemical diversity across ‘Entotheonella’ variants, we assessed BGC similarities with the Biosynthetic Gene Similarity Clustering and Prospecting Engine (BiG-SCAPE)^54^, which groups BGCs into gene cluster families (GCFs) and compares them to already characterized ones in the MIBiG database^55^. The visualized data in Fig. 3, thus, allowed us to assign BGCs to putatively known or orphan compound types and compare the BGC diversity across phylotypes.

We detected a total of 493 BGCs or BGC fragments in the ‘Entotheonella’ and ‘Proxinella’ genomes, assigned to 369 GCFs and grouped by biosynthetic pathway classes: thiotemplate-based pathways (NRPSs and PKSs), ribosomally synthesized and post-translationally modified peptides (RiPPs), terpenes or other. Within this network, only seven links to previously characterized BGCs with MIBiG database entries were identified. All these belonged to BGCs already identified earlier in ‘Entotheonella’^29,30,56^ (Table 1).

Five additional GCFs matched previously assigned BGC types lacking MIBiG entries, namely konbamides (for example **20**), keramamides (**3**), cyclotheonamides (**6**), nazumamide A (**4**) (Fig. 1 and Supplementary Fig. 1) and a partially characterized orphan proteusin from ‘*Ca*. E. factor’ TSY1 (ref. ^29^). The high percentage (96.7%) of unassigned GCFs and high GCF-to-BGC ratio (0.75) indicate considerable metabolic distinctness variation among the 18 newly analyzed symbionts.

In a previous study, we assigned the known polyketides and peptides from *T. swinhoei* Y to ‘E. factor’ TSY1 BGCs located on a plasmid (encoding pathways for onnamide and polytheonamide) and two genomic regions (encoding cyclotheonamide, konbamide, keramamide and nazumamide biosynthesis)^29^. The co-occurring symbiont ‘*Ca*. E. gemina’ contained exclusively orphan BGCs. Unexpectedly, analysis of single-bacterial genomes of ‘E. factor’ TSYB1, ‘E. gemina’ TSYB2 and ‘E. mitsugo’ TSYB3 in *T. swinhoei* YB collected at the same Japanese location suggested that all known NP BGCs belong to ‘E. gemina’ TSYB2 instead of ‘E. factor’ TSYB1. Reanalyzing the data of the initial study regarding potential misassignments did not reveal errors. The data also contradicted a misassignment of 16S rRNA genes, as core genomes of each ‘E. factor’ and ‘E. gemina’ pair were largely identical, including the orphan BGCs. This suggests the BGCs and plasmid were either exchanged between ‘Entotheonella’ variants or differentially acquired from another source. Supporting this, BGC mobility was also observed in another study that detected ‘E. factor’ BGCs in ‘E. serta’^31^. Beneficial properties of mobile BGCs, for example, in the context of host defense or colonization, might underly the observed symbiont retention or switching patterns.

To further assess chemical diversity, we manually reanalyzed BiG-SCAPE results (Fig. 3a) and evaluated BGC similarities across phylotypes. All three ‘E. serta’ variants (TCWA1, TCWB1 and TCBA2) from blue and white *Theonella* sponges share a substantial BGC repertoire, that is, 7–22 BGCs per symbiont pair (Supplementary Fig. 7). These include BGCs for related misakinolides and swinholides and a BGC for theonellamide. Genomes of different candidate species, however, contain mostly unique BGC sets (Supplementary Fig. 7).

Manual inspection and clinker^57^ analysis of BGCs classified as shared by BiG-SCAPE revealed that many grouped thiotemplated enzymes show only partial similarities (T-GCF-02 to T-GCF-09; Supplementary Figs. 8–10), while some related pathways were not grouped by BiG-SCAPE. One example is a staphyloxanthin-like BGC discovered in an earlier ‘Entotheonella’ study (named theoxanthin BGC)^35^. This BGC differs from typical NP clusters and, therefore, was missed in the antiSMASH analysis underlying the BiG-SCAPE network. We manually identified such BGCs in ten of 18 genomes and analyzed their relatedness using clinker^57^ (Supplementary Fig. 11). This showed highly conserved architectures that might, given their prevalence, indicate an important function for this candidate genus, possibly similar to staphyloxanthins that serve as antioxidant virulence factors during *Staphylococcus* *aureus* host colonization^58^.

Among the remaining highly similar loci, several were classified as RiPP-type, RiPP-like or RRE-containing by antiSMASH^59^ (Supplementary Figs. 12–15). However, all lacked identifiable precursor peptides, leaving their involvement in NP biosynthesis unclear. The few BGCs that were clearly shared by multiple phylotypes had architectures suggesting involvement in primary metabolic processes, that is, hopanoid, carotenoid, ectoine and pyrroloquinoline quinone biosynthesis. Additionally, a type III PKS BGC (Supplementary Fig. 16) found in nine of the 18 genomes was previously shown to encode biosynthesis of alkyl resorcinols and hydroquinones that might function as redox cofactors^56^. Thus, many antiSMASH^53^-detected shared BGC types likely belong to primary rather than secondary metabolism. An exception may be a BGC (T-GCF-03) encoding a bimodular hybrid NRPS-PKS with an unusual N-terminal thioesterase (TE) domain identified in eight genomes (Supplementary Fig. 9). Similarity searches revealed related enzymes with identical architecture in >50 phylogenetically diverse bacteria, mostly derived from eukaryotic hosts. However, these BGCs are uncharacterized.

We also checked the mOTUs database, the largest bacterial genome repository at the time of writing, for additional ‘Tectomicrobia’ members, finding 66 MAGs (Supplementary Table 5) beyond those reported here or in published work^29,30,60^. Of these, 64 are primarily non-‘Entotheonella’ members containing 3–5 shared BGCs that appear to belong to primary metabolites (carotenoid, hopanoid, ladderane and PKS-like type I fatty acid synthase) or single NRPS modules, indicating low chemical diversity. Of the two remaining MAGs, both assigned to ‘Entotheonella’, one from a sponge metagenome contains four contigs with PKS or NRPS genes. The second MAG remained BGC rich after manual curation to eliminate BGCs from primary metabolism (12 NP contigs and 21 in total). This MAG originated from soil, supporting previous 16S rRNA data suggesting the existence of terrestrial BGC-rich members of this candidate genus^39^.

In conclusion, the BGC analysis revealed high diversity and variability in predicted NP biosynthetic pathways and structures among the analyzed symbionts, with few BGCs assigned to known compounds. These findings warranted a closer examination of orphan pathways at the in silico and functional level.

### BGC candidates for orphan compounds and sponge cytotoxins

In our dataset, 357 of the 369 GCFs lacked assigned NPs. Of these, 310 represented unique BGCs. Such singletons were found in every ‘Entotheonella’ genome, suggesting, together with the numerous phylotypes encountered in this candidate genus, a large NP discovery resource. Examples of BGCs of as-yet unknown function are shown in Fig. 3b. They include several NRPS systems, one of them associated with a radical *S*-adenosylmethionine (rSAM) *C*-methyltransferase homolog as an unusual enzyme combination; another BGC with this feature is discussed below. Among the putative RiPP BGCs, an operon stood out that encodes a dioxygenase-RiPP recognition element fusion enzyme and homologs of the selenocysteine proteins SelA and SelB, suggesting noncanonical biochemistry. This BGC was also present in the ‘*Ca*. P. opulenta’ AC1 genome, along with BGCs for a keramamide-like NRPS, further RiPPs and other compound types.

With sequencing data from *D*. *dissoluta* available, we searched for a BGC candidate for discodermolide, an anticancer polyketide that had reached phase 1 clinical trials^61^. Analysis of methanolic sponge extracts confirmed the compound in the collected specimen (Supplementary Figs. 17 and 18). However, none of the three ‘Entotheonella’ genomes of *D*. *dissoluta* contained a convincing candidate. As we only sequenced the enriched filamentous symbiont fraction, the discodermolide producer might be an organism distinct from ‘Entotheonella’. Another missing BGC was the one encoding the biosynthetic pathway of discokiolides, depsipeptides reported from *D*. *kiiensis* collected at a different location from ours^62^. In agreement, an analysis of *D*. *kiiensis* extracts did not suggest that our specimens contained these compounds.

**Fig. 4:**
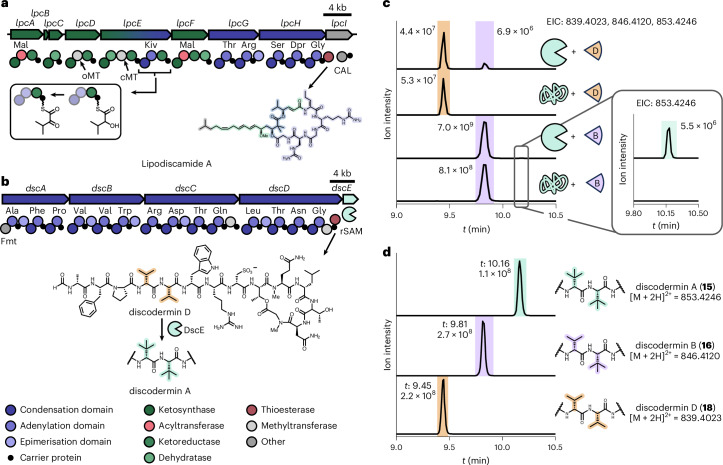
In silico prediction of lipodiscamide biosynthesis and biochemical study of discodermin biosynthesis. **a**, Putative lipodiscamide BGC. Shown above the NRPS and PKS proteins are the predicted substrate specificities for the respective A and AT domains. The module architectures and predicted substrate specificities fit well to the chemical structure of lipodiscamide A. As an example, the KR-catalyzed reaction of α-ketoisovaleric acid to the corresponding α-hydroxyl moiety is shown. A full biosynthetic scheme is provided in Extended Data Fig. 2. **b**, BGC encoding the discodermin NRPS. Shown above the NRPS proteins are the predicted substrate specificities for the respective A domains. A full biosynthetic scheme is provided in Extended Data Fig. 3. The reaction of the rSAM methyltransferase DscE acting on discodermin intermediates is shown. **c**, HPLC–HRMS traces of in vitro reconstitutions with native (pacman shape) or heat-denatured DscE (noodle shape) and discodermin D or discodermin B (wedges). Depicted are extracted ion chromatograms (EICs). Total ion counts of HPLC–HRMS runs are provided in Supplementary Fig. 21. **d**, HPLC–HRMS traces of isolated discodermin A (blue), B (purple) and D (orange) serving as analytical standards for reactions shown in **c** (color-coded similarly). HPLC–HRMS traces on all further control reactions are provided in Supplementary Fig. 23. Kiv, α-ketoisovaleric acid; CAL, CoA-acyl ligase; Fmt, formyltransferase.

**Extended Data Fig. 1:**
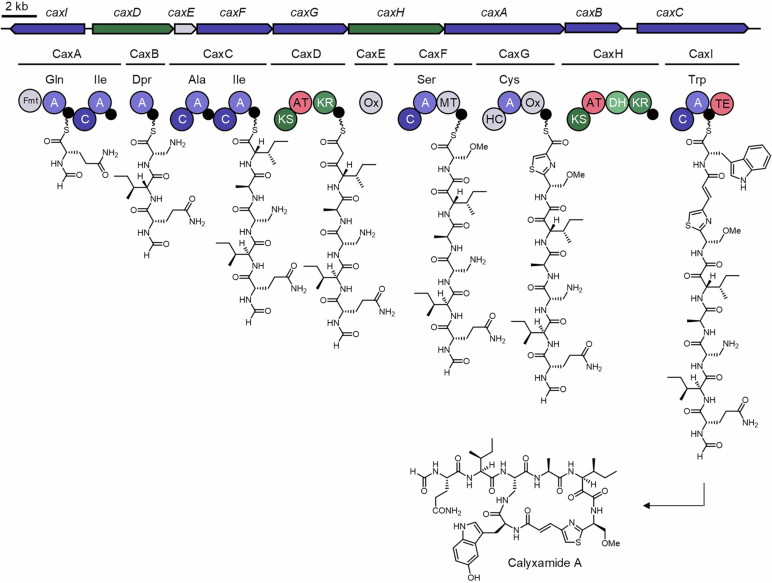
Proposed biosynthetic model for calyxamide biosynthesis. The modular architecture of the enzymes is shown with intermediates attached to the carrier proteins (visualized as black circles). Labels above the adenylation domains refer to the substrate specificity predicted by antiSMASH. Fmt, formyltransferase; A, adenylation domain; C, condensation domain; KS, ketosynthase; AT, acyltransferase; KR, ketoreductase; Ox, oxygenase; MT, methyltransferase; HC, heterocyclization domain; DH, dehydratase domain; TE, thioesterase; Dpr; 2,3-diaminopropionic acid.

**Extended Data Fig. 2:**
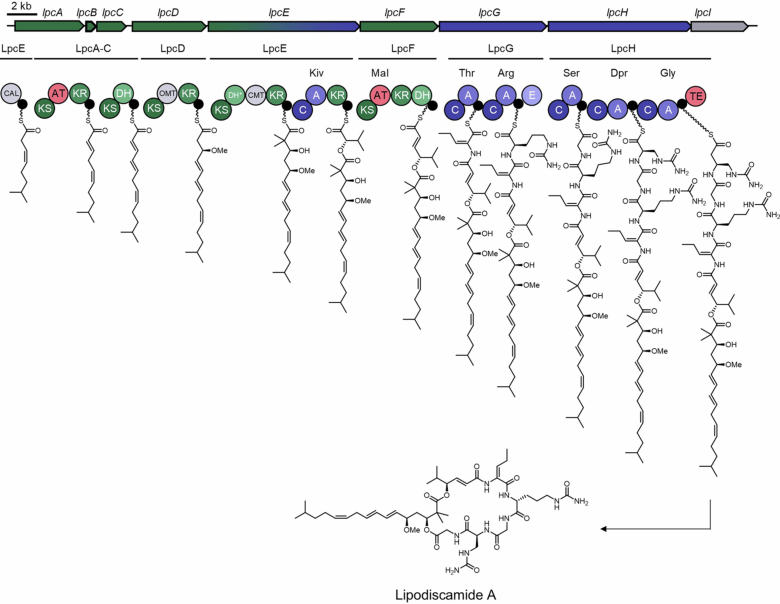
Proposed biosynthetic model for lipodiscamide biosynthesis. The modular architecture of the enzymes for lipodiscamide biosynthesis is shown with intermediates attached to the carrier proteins (visualized as black circles). Labels above the adenylation domains refer to the substrate specificity predicted by antiSMASH. CAL, coenzyme A ligase domain; KS, ketosynthase; AT, acyltransferase; KR, ketoreductase; DH, dehydratase domain; OMT, *O*-methyltransferase; CMT, *C*-methyltransferase; C, condensation domain; A, adenylation domain; E, epimerization domain; TE, thioesterase; Kiv, α-keto isovaleric acid; Dpr, 2,3-diaminopropionic acid; * = putatively non-functional or degraded domain due to a shortened amino acid sequence.

**Extended Data Fig. 3:**
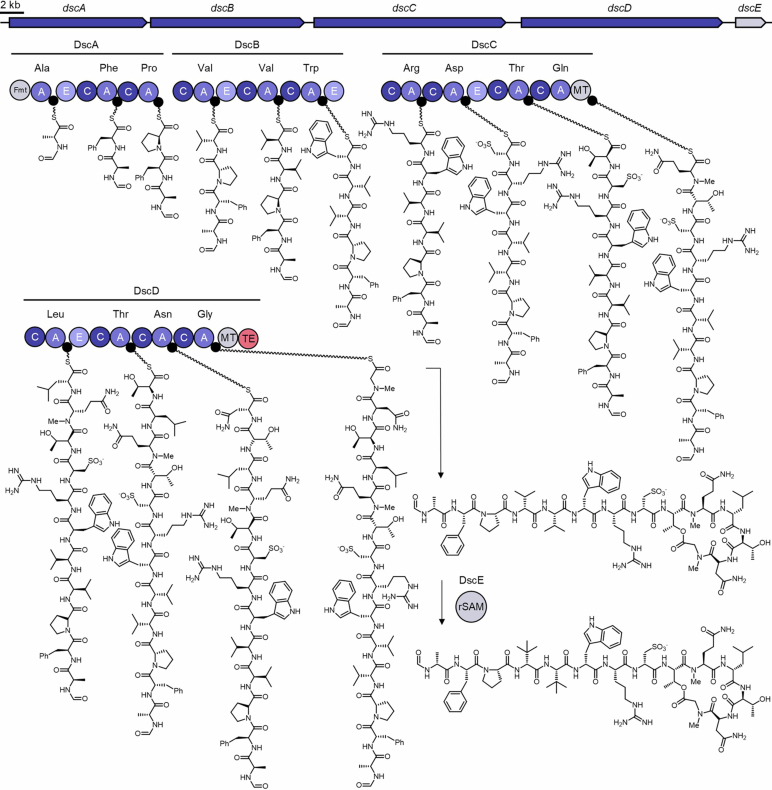
Proposed biosynthetic model for discodermin biosynthesis. The modular architecture of the enzymes for discodermin biosynthesis is shown with intermediates attached to the carrier proteins (visualized as black circles). Labels above the adenylation domains refer to the predicted substrate specificitiy by antiSMASH^11^. Fmt, formyltransferase; A, adenylation domain; E, epimerization domain; C, condensation domain; MT, methyltransferase; TE, thioesterase; rSAM, SAM-dependent methyltransferase.

In contrast to the missing discodermolide genes, we found three additional BGCs that architecturally matched reported sponge NPs. *D*. *calyx* from Shikine-Jima contains cytotoxic calyxamides (for example, **14**; Fig. 1)^63^, cyclic peptides with a formyl starter, a thiazole unit and two polyketide-like extensions, including an unusual C1 extension also found in keramamides (for example, **3**; Supplementary Fig. 1)^64^. Correspondingly, the ‘E. armillaria’ DC1 genome contains two regions with a keramamide-type BGC matching the calyxamide structure (Fig. 3b, Supplementary Tables 6 and 7 and Extended Data Fig. 1), suggesting this symbiont as the source. In the ‘E. monilis’ DK1 genome from *D*. *kiiensis*, we identified BGCs matching the cytotoxic lipodiscamides (for example, **20**; Fig. 1) and the discodermin antibiotics (**15**–**19**), known compounds from this sponge^40,41,65–69^. As a lipodiscamide candidate, the *lpc* BGC in ‘E. monilis’ DK1 encodes a PKS–NRPS machinery that shows perfect architectural agreement with the polyketide-peptide hybrid structure of **20** (Fig. 4a, Supplementary Tables 7 and 8 and Extended Data Fig. 2). This includes a characteristic NRPS module with a ketoreductase (KR) domain, previously reported to generate α-hydroxyacid residues, as present in the hydroxyisovalerate ester moiety of **20** (ref. ^70^). Four PKS modules are predicted to catalyze four elongations of a methyloctenoyl starter with methoxy and geminal dimethyl modifications introduced by *O*-methyltransferase and *C*-methyltransferase domains, respectively (Extended Data Fig. 2). The BGC also encodes a sulfotransferase homolog, consistent with the sulfonated lipodiscamides^41^. Another BGC (*dsc*) in ‘E. monilis’ DK1 encodes four NRPS proteins totaling 14 modules (Fig. 4b and Supplementary Table 9), including a predicted loading module with a formyltransferase domain. This feature and the overall order and predicted specificities (Fig. 4b and Supplementary Table 7) of adenylation (A) domains fit well with the tetradecapeptide structure of discodermins (**15**–**19**; Figs. 1 and 4b and Extended Data Fig. 3). The only deviation of this prediction from the final chemical structure is aspartic acid (Asp) as a substrate for the A domain in module eight of DscC instead of cysteic acid (Cya) present in discodermins. Additionally, epimerase and *N*-methyltransferase domains in some modules align with d-amino acids and *N*-methylated peptide bonds in discodermins.

Collectively, these analyses suggest that most compounds previously reported from the sponges are produced by ‘Entotheonella’, with the possible exception of discodermolides. To complement our in silico studies with functional data, we selected one tentatively assigned (*dsc*) and one orphan gene locus (*cmb*) for biochemical enzyme studies.

### RiPP-like modification in nonribosomal biosynthesis

An unusual feature of discodermins (**15**–**19**; Figs. 1 and 4b) is the presence of variants with optional *C*-methylations at three positions that contain alanine and valine in nonmethylated congeners. Because of these structural variants, we speculated that *C*-methylation might occur late in biosynthesis rather than through incorporation of premethylated amino acids. The *dsc* BGC encodes the protein DscE with homology to cobalamin-dependent rSAM methyltransferases, which typically catalyze radical *C*-methylations^71,72^. An extreme example is 16–17 *C*-methylations in polytheonamides (**8**) from ‘E. factor’ (refs. ^32,73–76^), peptides superficially similar to discodermins in their *t*-leucine residues and alternating dl-configurations. However, polytheonamides are RiPPs in contrast to the nonribosomally synthesized discodermins.

To interrogate the function of DscE, we reisolated the nonmethylated congener discodermin D (**18**) from *D*. *kiiensis* as a substrate for in vitro methylation. DscE was prepared by aerobically expressing its codon-optimized gene in *Escherichia* *coli* Tuner (DE3) as an N-terminally His_6_-tagged variant. The gene was coexpressed with the *Azotobacter* *vinelandii* *isc* operon for iron–sulfur cluster biosynthesis^77^ and native *btuCEDFB* genes for cobalamin uptake, previously reported to aid the production of B_12_-dependent rSAM enzymes^78^. After anaerobic purification using nickel affinity chromatography (Supplementary Fig. 19), iron–sulfur clusters and the cobalamin cofactor were anaerobically reconstituted by adding iron and sulfur sources (ammonium iron(II) sulfate, l-cysteine, cysteine desulfurase (IscS) and pyridoxalphosphate) and methylcobalamin (MeCbl) (Supplementary Fig. 20).

**Extended Data Fig. 4:**
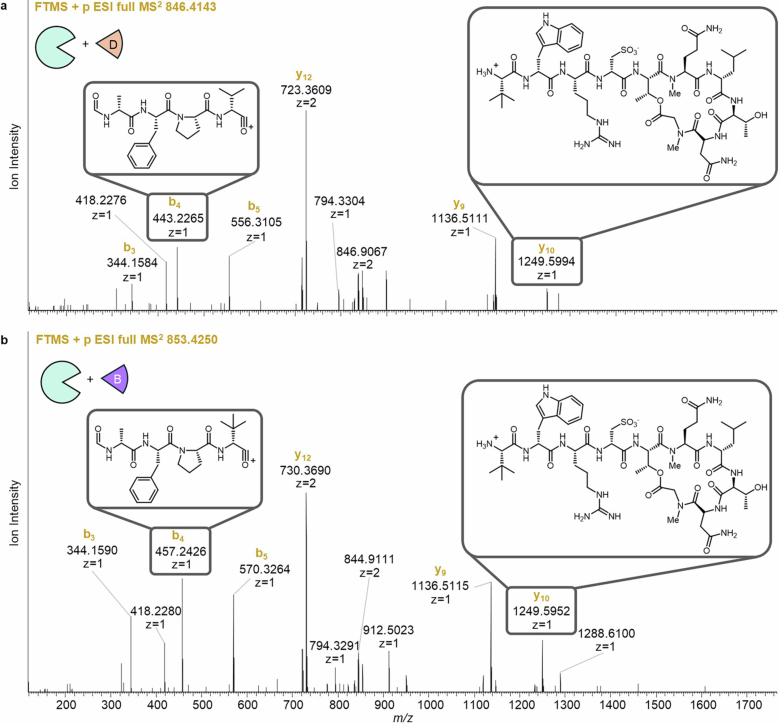
MS^2^ spectra of products from the in vitro reconstitution of rSAM DscE methyltransferase activity. The significant mass fragments for the respective product are depicted with potential fragments. **a**, Incubation of DscE with discodermin D (as visualized with the cartoon on the upper left). The structures in the boxes are potential fragments that fit to the respective fragmentation *m/z*. **b**, Incubation of DscE with discodermin B (as depicted with the cartoon on the upper left). The structures in the boxes are potential fragments that fit to the respective fragmentation *m/z*.

We tested the activity of DscE by incubation with unmethylated discodermin D, SAM, MeCbl and a reductant system (methyl viologen, DTT and NADPH) under anaerobic conditions. High-performance liquid chromatography–high-resolution mass spectrometry (HPLC–HRMS) analysis showed formation of a product with a 14-Da mass increase (Fig. 4c and Supplementary Fig. 21), localized to V5 by MS^2^ fragmentation (Extended Data Fig. 4). Comparison to authentic standards from *D*. *kiiensis* confirmed its identity as discodermin B (**16**) (Fig. 4d and Supplementary Figs. 22 and 23). When the methylation assay was repeated with monomethylated discodermin B (**16**) from *D*. *kiiensis* as substrate, small amounts of dimethylated peptide were detected with properties identical to discodermin A (**15**) (Fig. 4c).

These data on the RiPP-like discodermin modification and the good correspondence between NRPS architecture and discodermin structure support biosynthesis of these peptides by ‘E. monilis’ DK1. The origin of *N*-ethylglycine (EtGly) and Cya building blocks remains unclear. EtGly formation by *C*-methylation of A1 was not observed in our assays. EtGly, a known transamination product of the central metabolite 2-ketobutyrate, might be directly incorporated by the NRPS. Similarly, Cya, for which biosynthetic gene candidates were not identified, might first form as a free amino acid. This is supported by its structural similarity to Asp, the predicted A domain substrate of the corresponding NRPS module. While no gene candidates for previously described Cya biosynthetic enzymes^79,80^ were detected in the ‘E. monilis’ genome, Cya might be produced by another member of the sponge holobiont, including the host, as this amino acid is a known sponge metabolite^81^. *tert*-Leucine is a residue of several other predicted NRPS/PKS products and might likewise be generated by radical *C*-methylation, notably the promising sponge-derived anticancer drug candidates plocabulin^82,83^ and hemiasterlin^84^, both with unknown biosynthetic origin.

### Characterization of an orphan terpene pathway

A range of terpene NPs have been reported from *T*. *swinhoei*, including the isonitrile diterpene amitorine A^85^ and several steroids^86^. Inspection of our genome data revealed 50 predicted terpene biosynthetic loci across all 18 genomes (Fig. 2b). However, most exhibited architectures that suggest carotenoid or hopanoid biosynthesis. This agrees with our previous detection of carotenoids in single ‘Entotheonella’ filaments using Raman microscopy^46^.

**Fig. 5:**
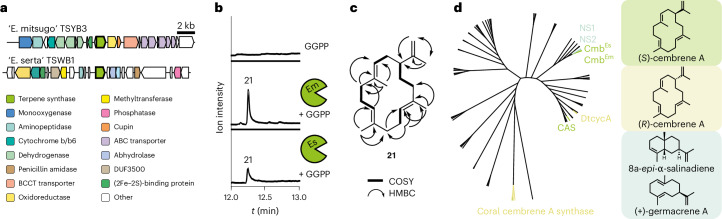
Terpene synthase responsible for cembrene A biosynthesis. **a**, BGCs containing the *cmb* gene (terpene synthase) found in the genomes of ‘E. mitsugo’ TSYB3 and ‘E. serta’ TSWB1. **b**, GC–MS traces of in vitro reconstitutions of Cmb with GGPP. **c**, Structure elucidation of **21**, product formed in the reaction of Cmb with GGPP. **d**, Phylogenetic tree of selected bacterial terpene synthases and the homolog catalyzing cembrene A (**21**) formation in soft corals^94,95^. GenBank identifiers of the sequence used to construct this tree are shown in Supplementary Table 11. NS1, 8a-*epi*-α-selinene synthase from *Nostoc* sp. PCC 7120; NS2, (+)-germacrene A synthase from *Nostoc* *punctiforme* PCC 73102; DtcycA, (*R*)-cembrene synthase from *Streptomyces* sp. SANK 60404; CAS, (*S*)-cembrene synthase from *Allokutzneria* *albata*.

Among terpene loci lacking carotenoid-type and hopanoid-type genes, we identified genome regions in the *Theonella* sp. 1 BA symbiont ‘E. serta’ TCBA2, the *T*. *swinhoei* YB symbiont ‘E. mitsugo’ TSYB3, the *T*. *swinhoei* WB symbiont ‘E. serta’ TSWB1 and the *T*. *swinhoei* WA symbiont ‘E. serta’ TSWA1 with closely related genes encoding a predicted class I terpene synthase, termed Cmb. Each *cmb* gene was embedded in a distinct genomic environment with unclear roles in NP biosynthesis (Fig. 5a and Supplementary Fig. 24). To assess the terpene synthase function, we selected two of the four enzymes for further analyses. We heterologously expressed codon-optimized genes from ‘E. mitsugo’ TSYB3 (named *cmb*^*Em*^) and ‘E. serta’ TSWB1 (*cmb*^*Es*^) (Supplementary Table 10) in *E*. *coli* BL21 (DE3) as N-terminally His_6_-tagged proteins (Supplementary Fig. 25a). Incubation with geranyl pyrophosphate (GPP), farnesyl pyrophosphate (FPP) or geranyl-GPP (GGPP) in vitro and analysis by gas chromatography (GC)–MS (Supplementary Fig. 25b) revealed the formation of geraniol (Supplementary Fig. 26) and several sesquiterpenes (Supplementary Fig. 27) when incubated with GPP and FPP, respectively.

**Extended Data Fig. 5:**
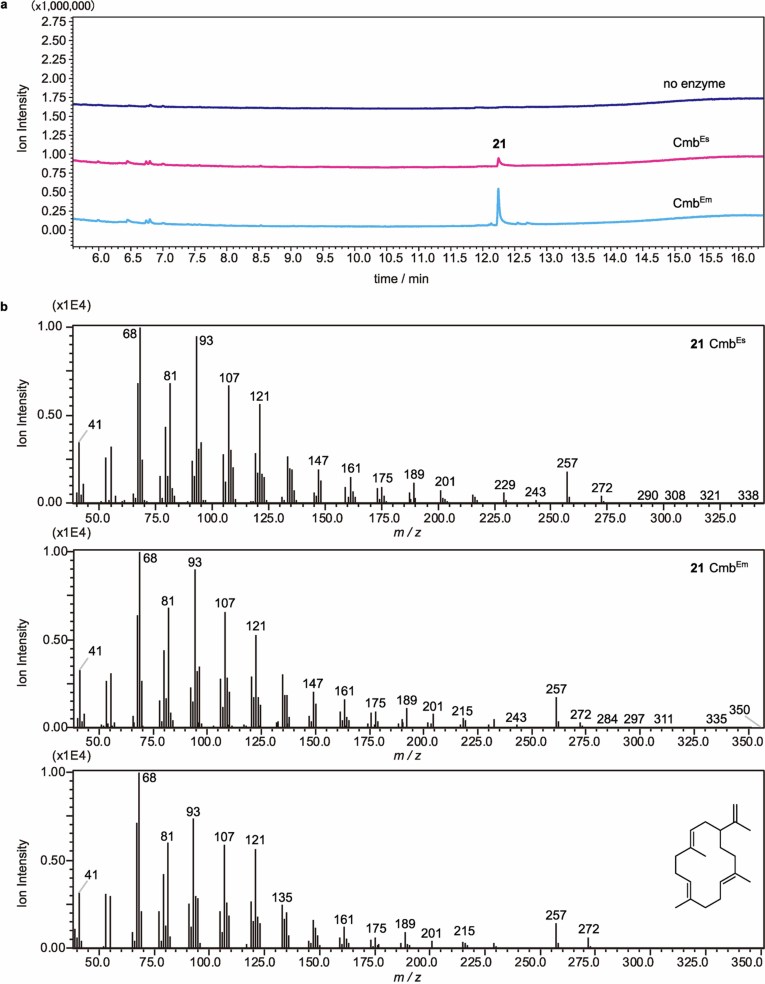
In vitro reconstitution of the terpene synthase Cmb with GGPP as a substrate. **a**, GC-MS TIC traces of ethyl acetate extracts of in vitro assays with FPP and terpene synthases. **b**, EI mass spectra of the reaction product of Cmb^Es^ (top), Cmb^Em^ (middle) and reference spectrum for cembrene A (bottom).

However, incubating GGPP with either Cmb^Em^ or Cmb^Es^ resulted in the formation of a single compound with identical retention times and mass spectra in both cases (Fig. 5b and Extended Data Fig. 5). This compound was purified from a preparative-scale enzymatic reaction using Cmb^Es^ from ‘E. serta’, yielding 0.8 mg of product. Nuclear magnetic resonance (NMR)-based structure elucidation (Fig. 5c, Extended Data Table 1 and Supplementary Figs. 28–32) identified the terpene as cembrene A (**21**). Optical rotation measurements and comparison to literature values^87,88^ established the compound as (*S*)-cembrene A ([α]_D_^20^ = +5.7).

## Discussion

This study provides deeper insights into sponge symbionts of the candidate genus ‘Entotheonella’. We show that large and variable sets of unique BGCs are a consistent feature across the investigated members of this lineage. Moreover, among four types of biosynthetically unassigned polyketides and modified peptides previously reported from the selected sponges (discodermolides, calyxamides, lipodiscamides and discodermins), three were bioinformatically or functionally linked to ‘Entotheonella’ BGCs, supporting widespread roles of these symbionts as producers of structurally complex sponge NPs. The data also suggest the existence of a second BGC-rich taxon, ‘Proxinella’, within the candidate phylum ‘Tectomicrobia’ (ref. ^39^). While defensive roles were demonstrated for some sponge NPs^8^, rigorous ecological studies are needed to test this hypothesis for the ‘Entotheonella’ compounds. Alternative functions may be to mediate interactions within the microbiome or to aid the producer in sponge colonization, for example.

Various non-‘Entotheonella’ symbionts were previously bioinformatically or functionally linked to sponge NPs. These include a Chloroflexi member in *T*. *swinhoei* as the aurantoside source^46^, various polyketide-producing bacteria in *Mycale* *hentscheli*^20,21^, cyanobacteria producing halogenated compounds in dysideid sponges^89^, an intracellular renieramyin-producing gammaproteobacterium in a *Haliclona* sponge^90^ and diverse bacteria producing halogenated RiPPs^91^. In addition, sponge hosts have been demonstrated to synthesize some terpenes^12^ and peptides^13,92^. Our identification of the ‘Entotheonella’ product cembrene A was unexpected. Cembranoids are known from various organisms including corals and plants^93^. Recent biochemical studies assigned cembrene biosynthesis in sponges and octocorals^12,94,95^ to the animals. Our data show that terpenes of marine invertebrates can have diverse origins even for identical compounds. In agreement, cembrene A cyclases were also reported from actinomycetes^96,97^. However, their sequences greatly differ, suggesting convergent evolution (Fig. 5d and Supplementary Fig. 33). Phylogenetically, the two ‘Entotheonella’ homologs were more similar to cyanobacterial 8a-*epi*-α-selinene and germacrene A cyclase than to other cembrene cyclases (Fig. 5d).

To date, ‘Entotheonella’ cultivation attempts have been unsuccessful except for one report on a mixed culture^23^. The genome data provided here and in earlier studies^29,35^ might aid targeted cultivation approaches to access the diverse chemistry of this talented producer taxon. The available genetic information on assigned and orphan pathways might also enable additional supply strategies, including heterologous BGC expression and the targeted search for alternative culturable producer organisms containing homologous genes^98^, approaches likely to become increasingly successful with current and future genome initiatives.

## Methods

### General

Our research complied with all relevant ethical regulations.

The sample size for sponge samples was chosen on the basis of their availability: one for *A*. *cribrophora*, *D*. *calyx*, *D*. *kiiensis* and *T*. *swinhoei* WA; two for *T*. *swinhoei* WB and *Theonella* sp. 1 BA; three for *D*. *dissoluta* and *T*. *swinhoei* YB; four for *Theonella* sp. 2 BT. No statistical analyses were included in this study and none of the sponge specimens were excluded from our analyses.

### Sponge collection

Information on sponge collection sites and dates are provided in Supplementary Table 1. For each specimen, one sample was subjected to metagenomic sequencing as described below (Extended Data Table 2).

### Protocol A—enrichment of filamentous bacteria and DNA isolation

All protocol variants were applied to freshly collected sponges unless stated otherwise.

#### *T*. *swinhoei* WA and *T*. *swinhoei* WB

The enrichment of filamentous bacteria and subsequent DNA isolation and sequencing were conducted in a previous study^30^. The sequence dataset of that study was used for reanalysis as described below.

#### *Theonella* sp. 1 BA and *D*. *dissoluta*

Filamentous bacteria were mechanically enriched before DNA isolation using a modified method reported by Bewley et al.^25^. Sponge tissue (1 cm^3^) was soaked in 10 ml of calcium-free and magnesium-free artificial sea water (CMF-ASW; 10 mM Tris-HCl pH 8, 2.5 mM EGTA, 2.15 mM NaHCO_3_, 33 mM Na_2_SO_4_, 9 mM KCl and 449 mM NaCl). After overnight incubation at 4 °C under gentle mixing, the tissue was cut into small pieces using a sterile scalpel and transferred to a new 15-ml conical tube. The sample was submerged in PBS (8.4 mM Na_2_HPO_4_, 1.5 mM KH_2_PO_4_ and 150 mM NaCl, pH 7.5, sterile-filtered and stored at room temperature), collagenase enzyme (1 µl per ml of PBS; final concentration: 240 µg ml^−1^) was added and the mixture was incubated at 37 °C for 1 h. Subsequently, 10 ml of CMF-ASW was added and the sponge tissue was incubated at 4 °C for 2.5 h while mixing gently. After passing the sample through a 40-µm nylon filter into a 50-ml conical tube, the retained sponge tissue was transferred to a sterile mortar and ground with a pestle. The ground sample was then filtered through another 40-µm nylon filter into a new 50-ml conical tube and the filter was washed with 10–15 ml of CMF-ASW. The filtrates were combined and centrifuged for 10 min at 700*g* to sediment tissue and bacterial cells. The supernatant was carefully transferred into a new tube and the pellet was resuspended in 10 ml of CMF-ASW and centrifuged for 10 min at 20*g* to remove sponge tissues and unwanted debris. The supernatant was then transferred into a new 15-ml conical tube and the pellet was resuspended in 6 ml of CMF-ASW followed by another centrifugation step (10 min at 200*g*) to remove unicellular bacteria. The supernatant was again transferred to a new 15-ml conical tube and the pellet was resuspended again in 6 ml of CMF-ASW for another round of centrifugation (10 min at 200*g*) to further wash the now enriched filamentous bacterial cells. All centrifugation steps were performed at 4 °C. The cell fractions were assessed by microscopic analysis of each fraction. The DNA isolation was performed from enriched filamentous bacterial cells. For this, 1.2 ml of the 6 ml of enriched filamentous bacterial cells were used to pellet the filamentous bacteria (centrifugation for 3 min at maximum speed). The supernatant was removed and the pellet was resuspended in 250 µl of resuspension buffer (30 mM Tris-HCl pH 8.0, 1 mM EDTA and 0.1% SDS) supplemented with 15 µl of proteinase K solution (20 mg ml^−1^). After incubation for 30 min at 50 °C, the treated cells were cooled on ice for 2 min followed by centrifugation at maximum speed for 5 min. The supernatant was extracted with one volume of phenol, chloroform and isoamyl alcohol (25:24:1, v/v/v). The extraction was centrifuged at 9,000*g* for 10 min and the aqueous phase was transferred to a fresh tube. After another two rounds of extraction with one volume of chloroform, the aqueous phase was transferred to a precooled tube and DNA precipitation was performed by addition of one volume of cold 2-propanol and 0.1 volumes of 3 M sodium acetate and overnight incubation at −20 °C. The sample was centrifuged at maximum speed for 30 min and the supernatant was carefully removed. The resulting DNA pellet was washed twice with one volume of 70% ethanol (centrifugation at maximum speed for 10 min), followed by drying under a sterile hood for 5 min. The DNA was resuspended in 30 µl of elution buffer NE (Macherey-Nagel, NucleoSpin) and incubated overnight at 4 °C. To further remove RNA from the sample, the DNA solution (28 µL) was treated with 1.5 µl of RNase A (10 mg ml^−1^) and incubated at 30 °C for 70 min. The treated DNA solution was then precipitated for 1 h at −20 °C with isopropanol and sodium acetate (1:0.1, v/v) following the same procedure as described above for washing the DNA and subsequent dilution of the DNA pellet overnight at 4 °C. The quality of the DNA was assessed by absorption at 230 nm, 260 nm and 280 nm and by gel electrophoresis.

### Protocol B—metagenomic DNA extraction from sponge samples

#### *A*. *cribophora*

The sponge was stored in RNAlater at −20 °C upon collection. The DNA of this sponge was isolated as previously reported by Peters et al.^39^. In brief, the defrosted sponge was rinsed with ASW before it was minced and homogenized using a Precellys 24 homogenizer (Bertin). The DNA was then isolated using the standard protocol of the DNeasy PowerSoil pro kit (Qiagen). Additionally, high-molecular-weight (HMW) DNA was isolated for Oxford Nanopore sequencing using the MagAttract HMW DNA kit (Qiagen), following the ‘disruption/lysis of tissue’ protocol according to the manufacturer’s instructions, followed by the ‘manual purification of HMW genomic DNA from fresh or frozen tissue’ protocol. Sponge pieces were weighed after thawing and squeezed to remove RNALater but were not rinsed. Samples were treated following the set of standard protocols mentioned above, except gentle mixing was used instead of vortexing and only a Pipetman P1000 pipette was used to handle the DNA.

#### *T*. *swinhoei* YB, *D*. *calyx* and *D*. *kiiensis*

Single-bacterial genome sequencing of ‘Entotheonella’ was conducted as previously described^46^. In short, the sponge tissue was minced in CMF-ASW and the fraction that passed through a 40-µm mesh was collected as the bacterial fraction. Then, filamentous bacteria were enriched by centrifugation at 1,000*g*. After 30 s, the supernatant was collected and the pellet was isolated at 10 min. Sponge tissue or unicellular organisms were removed by each step. Filamentous bacteria were suspended in PBS and lysed by Ready-lyse lysozyme (Epicentre; 10 U per µl, 37 °C, 30 min), proteinase K (Promega; 1 mg ml^−1^, 50 °C, 30 min) and heat treatment (95 °C, 15 min). The DNA was purified with a DNeasy blood and tissue kit (Qiagen) from the lysate.

### Protocol C—acquisition of single-amplified genomes (SAGs)

#### *T*. *swinhoei* YB, *D*. *calyx, D*. *kiiensis* and *Theonella* sp. 2 BT

Single-bacterial genome sequencing of ‘Entotheonella’ was conducted as previously described^46^. In short, the sponge tissue was minced in CMF-ASW and the fraction that passed through a 40-µm mesh was collected as the bacterial fraction. Then, filamentous bacteria were enriched by centrifugation at 1,000*g*. After 30 s, the supernatant was collected and the pellet was isolated at 10 min. Sponge tissue or unicellular organisms were removed by each step. Filamentous bacteria were suspended in PBS and encapsulated into microdroplets with a diameter of 50 µm (ref. ^46^). The droplets containing single ‘Entotheonella’ filaments were manually picked with a micropipette (Drummond) under microscopic observation and isolated into 0.2-ml PCR tubes. The isolated bacteria were lysed by Ready-lyse lysozyme (Epicentre; 10 U per µl, 37 °C, 30 min), proteinase K (Promega; 1 mg ml^−1^, 50 °C, 30 min) and heat treatment (95 °C, 15 min). To acquire single-bacterial amplified ‘Entotheonella’ genomes, multiple displacement amplification (MDA) was performed for 3 h with the REPLI-g single-cell kit (Qiagen). The MDA reactions were performed with 40 single filaments each from *D*. *calyx* and *D*. *kiiensis* and 96 single filaments each from *T*. *swinhoei* YB and *Theonella* sp. 2 BT.

### DNA sequencing

#### *T*. *swinhoei* WA and *T*. *swinhoei* WB

The isolated metagenomic DNA from the fraction of enriched filamentous bacteria was sequenced in a previous study^30^ and was here subjected to an improved binning analysis as described below.

#### *Theonella* sp. 1 BA and *D*. *dissoluta*

The isolated metagenomic DNA from the enriched filamentous bacterial fraction was sequenced by the Functional Genomics Center Zürich using an Illumina HiSeq2500 system.

#### *A*. *cribophora*

The metagenomic DNA samples isolated from this sponge were sequenced by Novogen Europe using the Illumina Novaseq600 platform and the PE150 library and sequencing kits^39^. Additionally, the extracted HMW DNA was sequenced in two rounds using an Oxford Nanopore Technologies MinION Mk1C. For the first round, the ligation sequencing kit SQK-LSK109 (Oxford Nanopore Technologies), the NEBNext Companion Module for the Oxford Nanopore Technologies ligation sequencing kit (New England Biolabs) and NBD104 barcodes (Oxford Nanopore Technologies) were used to make the sequencing libraries, following the ‘ligation sequencing gDNA—native barcoding’ (SQK-LSK109 with EXP-NBD104) protocol. The second sequencing library was made using the same kits, except new barcodes from the NBD114 kit (Oxford Nanopore Technologies) were used and the ‘ligation sequencing gDNA—native barcoding’ (SQK-LSK109 with EXP-NBD104 and EXP-NBD114) protocol was followed. Then, 200-ng samples of five sponge libraries were combined for the final sequencing library. The first round of sequencing was performed on an already used, flushed flowcell (R9.4.1), with approximately 590 pores available. The second round of sequencing was performed on a new flowcell (R9.4.1), with approximately 1,301 pores available.

#### *T*. *swinhoei* YB, *D*. *calyx*, *D*. *kiiensis* and *Theonella* sp. 2 BT

Sequencing libraries were prepared from each SAG using the Nextera XT kit and short-read sequencing with MiSeq (Illumina) was conducted. Additionally, sequencing libraries were prepared with the rapid sequencing kit (Nanopore) from metagenomic DNA of the isolated filamentous bacterial fraction and sequenced by MinION (Nanopore) using the flowcell R9.4.1. Regardless of the genome construction method, at least nine MDA products were used to determine the genome of each variant.

### Assembly and binning

#### *T*. *swinhoei* WA and *T*. *swinhoei* WB

DNA sequencing, assembly and binning was performed in a previous study^34^. For the work on *T*. *swinhoei* WB conducted in that study, the binning generated a single, inseparable bin containing the genomes of two ‘*Ca*. Entotheonella’ genomes at near-identical coverage. The assembly of the ‘*Ca*. E. serta’ TSWA1 single-bacterial genome from *T*. *swinhoei* WA^34^ now allowed a refinement of this bin into sequences of very high identity (‘*Ca*. E. serta’ TSWB1) and moderate identity (‘*Ca*. E. consors’ TSWB2) and a small fraction with no apparent homology (unknown source). The latter was discarded.

#### *Theonella* sp. 1 BA and *D*. *dissoluta*

The raw reads were assembled using SPAdes and binned on the basis of tetranucleotide frequency and sequence coverage in a process described in more detail below. For the quality control of the metagenomes, BBDuk (version 37.55, Joint Genome Institute) was first used in right-trimming mode with a *k*-mer length of 23 down to 11 and a Hamming distance of 1 to filter out sequencing adaptors. A second pass with a *k*-mer length of 31 and a Hamming distance of 1 was used to filter out PhiX sequences. A third and final pass performed quality trimming on both read ends with a Phred score cutoff of 14 and an average quality score cutoff of 20, with reads under 45 bp or containing Ns subsequently rejected. When a metagenomic assembly required more than 3 TB of RAM to complete, the reads were first *k*-mer-normalized with BBNorm (version 37.55, Joint Genome Institute) using a minimum depth of 2 and target depth of 80. The normalized paired-end and unnormalized singleton reads of each read set were assembled using metaSPAdes^99^ (version 3.11.0) without the error correction module but otherwise default parameters. Scaffolds smaller than 1 kbp were then filtered out. For the binning, the quality-controlled paired-end reads were aligned to the assembled scaffolds using BWA (version 0.7.17)^100^ and then filtered with a Python script for an identity of at least 97%, an alignment length of 200 bp and a minimum alignment coverage of 90% of the read length. The alignments were then sorted by SAMtools (version 1.9)^101^. Coverage depth across the scaffolds was calculated using the MetaBAT2 (version 2.12.1)^102^ jgi_summarize_bam_contig_depths script and this information was then used by MetaBAT2 to bin the scaffolds with default parameters.

#### *A*. *cribophora*

Metagenomic reads were processed for quality trimming as described in Peters et al.^39^. Using BBtools suite (version 37.64) with parameters ktrim = r, k = 23, mink = 7, hdist = 1, tpe, tbo, qtrim = rl, trimq = 20, ftm = 5, maq = 20 and minlen = 50, adaptors were removed and quality filtering and normalization were performed. The short and long reads were used for a hybrid assembly using metaSPAdes (version 3.12) with the ‘--only-assembler’ flag. Binning was performed using MetaWRAP (version 1.2) with minimum completeness of 50% and maximum contamination of 10%, as in Peters et al.^39^.

#### *T*. *swinhoei* YB, *D*. *calyx* and *D*. *kiiensis*

The quality of acquired short reads for each SAG was controlled with fastp (version 0.20.0)^103^ (options: -q 25-r -x) and de novo assembly with SPAdes (version 3.12.0)^104^ (option: --sc-careful) was implemented. Then, the taxonomy and genome completeness of the SAG contigs were evaluated with CheckM (version 1.0.6)^47^ and QUAST (version 4.5)^105^. On the basis of the ccSAG^106^ method, strain-level clustering of ‘Entotheonella’ SAGs was implemented. Then, ‘Entotheonella’ long reads were extracted by short-read mapping with SAGs of each ‘Entotheonella’ strains. Lastly, draft genomes of ‘Entotheonella’ were acquired by de novo assembly of long reads by Canu (version 1.4)^107^ and polished by Pilon (version 1.22)^106^ using short reads of the same strain. For *T*. *swinhoei* YB, the raw reads generated in the study by Wilson et. al.^29^ were merged with the newly generated single-bacterial assembled data to produce a hybrid genome.

#### *Theonella* sp. 2 BT

The quality of acquired short reads for each SAG was controlled with fastp (version 0.20.0)^103^ (options: -q 25-r -x) and de novo assembly with SPAdes (version 3.12.0)^104^ (option: --sc-careful) was implemented. Then, the taxonomy and genome completeness of the SAG contigs were evaluated with CheckM (version 1.0.6)^47^ and QUAST (version 4.5)^105^. Finally, strain-level clustering of ‘Entotheonella’ SAGs and coassembly of clustered SAGs were implemented on the basis of the ccSAG^106^ method.

### Additional genome processing

The quality of the bins was assessed using the CheckM (version 1.0.13)^47^ lineage workflow, which included taxonomic assignment and the generation of summary plots. Bins with ≥90% completeness and ≤5% contamination were deemed of high quality, those with ≥70% completeness and ≤10% contamination were deemed of good quality, those with ≥50% completeness and ≤10% contamination were deemed of medium quality and any bins with <50% completeness or >10% contamination were deemed of low quality. Genes were predicted with Prodigal (version 2.6.3)^108^ in meta mode (-p meta) with the closed end (-c) and mask Ns (-m) options. Contigs were taxonomically identified with Kaiju (version 1.6.2)^109^ against a provided subset of the National Center for Biotechnology Information BLASTnr database containing all proteins belonging to archaea, bacteria, viruses, fungi and microbial eukaryotes (nr_euk).

Contigs that were independently sequenced in other studies^29,34,36,37^ were manually scaffolded if applicable using published sequence data. Where SAG data were available that corresponded to phylotypes also identified in metagenomic samples (*T*. *swinhoei* WB and *Theonella* sp. 1 BA), BLAST searches were used to retrieve additional nonbinned contigs where they could be unambiguously assigned to a draft MAG. After genome assembly, all genomes were scaffolded using the Multi-CSAR^110^ reference-based contig scaffolder, using each other as references, resulting in a notable reduction in the number of contigs and improved N50 and L50 values (Supplementary Table 2). Contigs below 500 bp in length were removed.

Evaluation of sequence quality was performed using the lineage_wf command in CheckM^47^. Phylogenomic analysis was performed using the online tool autoMLST^50^. The default nearest organisms and default MLST genes were selected, IQ-TREE Ultrafast Bootstrap was performed analysis with 1,000 replicates, ModelFinder was run, inconsistent MLST genes were filtered, the fast alignment mode (MAFFT FFT-NS-2) was implemented and a concatenated alignment was created. FastANI^49^ analysis was performed using the comparison mode ‘many to many’ and a matrix was created (Supplementary Fig. 3). Annotation of genes was performed with RASTtK^111^.

### Tree-building methods

#### Automated multilocus species tree

The autoMLST tree in Fig. 2b was generated using the autoMLST tool (https://automlst.ziemertlab.com/index)^50^ in de novo mode. The pipeline selects single-copy genes present in the organism set and infers a tree from the extracted sequences. The default nearest organisms and default MLST genes were selected, IQ-TREE Ultrafast Bootstrap analysis (1,000 replicates) was performed, ModelFinder was run, inconsistent MLST genes were filtered, fast alignment mode (MAFFT FFT-NS-2) was implemented and a concatenated alignment was generated. To run a larger dataset, the closest genomes to the symbiont genome of *A*. *cribophora* were identified with GTDB-Tk^51^ and used as reference genomes to run autoMLST locally and generate Supplementary Fig. 2. The same options were used including filtering of genes with inconsistent phylogeny (as described in the autoMLST methods) to safeguard against possible contamination. A list of the selected genes and functions can be found in Supplementary Table 12.

#### 16S rRNA gene analysis and tree-building methods

16S rRNA genes or fragments were identified using the ssu_finder method incorporated in CheckM^47^. The sequences were then aligned using MUSCLE Alignment in Geneious 8.1.9 and the following settings: maximum number of iterations, 16; optimization, diagonal (keep tree from iteration 1; distance measure in iteration 1, kmer4_6; clustering method in iterations 1 and 2, UPGMB; tree-rooting method in iterations 1 and 2, pseudo; sequence weighting scheme in iterations 1 and 2, CLUSTALW; terminal gaps, half penalty; anchor spacing, 32; diagonals, min length = 24; minimum column anchor scores, min best = 90; hydrophobicity, multiplier = 1.2); optimization, anchor (keep tree from iteration 2; distance measure in subsequent, pctif_kimura; clustering method in subsequent, UPGMB; tree-rooting method in subsequent, pseudo; sequence weighting scheme in subsequent, CLUSTALW; objective score, spm; gap open score = −1; diagonals, margin = 5; minimum column anchor scores, min smoothed = 90; hydrophobicity, window size = 5).

To infer trees, only complete genes (more than 1,500 nt) were taken into account for comparison to the complete gene deposited for ‘*Ca*. E. palauensis’ (AF130847.1). The trees were generated in MEGA7 (ref. ^112^) using the neighbor-joining and maximum-likelihood methods. More details can be found in the figure caption of Supplementary Fig. 5.

#### Construction of the phylogenetic tree for the Cmb terpene synthase

The tree was inferred through the tree builder function of Geneious 8.1.9 (alignment type, global alignment with free end gaps; cost matrix, Blosum45; genetic distance model, Jukes–Cantor; tree-building method, neighbor joining; gap open penalty, 8; gap extension penalty, 2).

### Biochemical studies

#### BGC analysis

To evaluate the biosynthetic potential of the analyzed genomes, antiSMASH7^59^ was run on all genomes using the webserver (https://antismash.secondarymetabolites.org/#!/start) in relaxed mode and with all extra features selected (KnownClusterBlast, ClusterBlast, SubClusterBlast, MIBiG cluster comparison, ActiveSiteFinder, RREFinder, Cluster Pfam analysis, Pfam-based GO term annotation, TIGRFam analysis and TFBS analysis). All the gbks files from the antiSMASH output were combined and analyzed using BiG-SCAPE with the following parameters: -v --mode auto --mibig21 --mix --cutoffs 0.5 --include_singletons. The output was further processed with Cytoscape (version 3)^113^. Here, the network file for the group labeled mix was used for further visualization. The nodes were divided into biosynthetic groups of thiotemplate-based pathways, RiPPs, terpenes and other. The latter contained all BGCs for indoles, ladderanes, ectoine, phosphonates and homoserine lactones but not for thiotemplate-based, RiPP or terpene biosynthesis. Furthermore, BGCs were manually analyzed for reoccurring modular architectures within the different genomes and occurrence of protein families on the basis of automated gene annotations generated with RASTtk and BLAST searches.

#### Isolation of discodermins

Frozen sponge specimens of *D*. *kiiensis* (100 g, wet weight) were extracted with methanol and the extract was partitioned between *n*-butanol and water. The *n*-butanol fraction was then fractionated by octadecylsilyl flash chromatography (C18-prep, Nacalai Tesque, Japan) using a stepwise gradient with H_2_O and methanol (0% to 100% methanol). The 80% methanol fraction was further purified by HPLC (Cosmosil MS-II, 10 × 250 mm; Nacalai Tesque) using CH_3_CN:H_2_O (2:3) containing 0.05% trifluoroacetic acid. Finally, another round of HPLC, using the same column with CH_3_OH:H_2_O (7:3) containing 0.05% trifluoroacetic acid, was performed to obtain discodermin A (10.2 mg), discodermin B (4.5 mg) and discodermin D (3.4 mg).

#### Overexpression of *dscE*

The gene *dscE* was codon-optimized for *E*. *coli* and synthesized by Twist Bioscience with an N-terminal His_6_ tag and within the pET-28a(+) backbone. Electrocompetent NiCo21(DE3) *E*. *coli* cells (New England Biolabs) were transformed with the pET-28a(+)-DscE plasmid (KanR) together with the plasmids pDB1282 (AmpR)^77^ and pBAD42-BtuCEDFB (SpecR)^78^. Precultures were prepared in Luria–Bertani (LB) medium supplemented with the appropriate antibiotics and incubated overnight at 37 °C and 180 rpm. Then, 500 ml of terrific broth (TB) medium supplemented with 50 ml of glycerol, appropriate antibiotics, 0.5 mM δ-aminolevulinic acid, 300 mM CoCl_2_ and 1.5 mM MeCbl were inoculated with 1% preculture and incubated at 37 °C and 180 rpm. At an optical density at 600 nm (OD_600_) of 0.3, a 20-ml aliquot was taken (noninduced); protein expression of pDB1282 and pBAD42-BtuCEDFB was induced with 0.2% (w/v) l-arabinose and the medium was supplemented with 50 mM ammonium iron(II) sulfate and 300 mM l-cysteine. The culture was further incubated at 37 °C and 180 rpm until an OD_600_ ≈ 1.0 was reached (half-induced). After cooling the culture at 4 °C for 30 min, protein expression of pET-28a(+)-DscE was induced with 1 mM IPGT. The culture was incubated overnight at 16 °C and 140 rpm.

#### Purification of DscE

After an aliquot was taken (induced), cells from the overexpression culture were harvested by centrifugation at 6,000*g* for 20 min and 4 °C. The pellet was transferred into an anaerobic chamber and dissolved in 1 ml of lysis buffer (50 mM HEPES pH 7.8, 300 mM KCl, 0.05% (v/v) Triton X-100, 10% glycerol and 20 mM imidazole) per 0.1-g pellet. The cells were sonicated four times for 2 min each at 20% amplitude, alternating between 5 s on and 5 s off (total lysate). The total lysate was cleared by centrifugation for 5 min at 14,000*g* and aliquots of the supernatant and the pellet were taken. The supernatant was incubated with 1 ml of Protino Ni-NTA agarose (Macherey-Nagel) for 1 h at 4 °C. After transferring the suspension onto an appropriate column, the flowthrough was collected. The sample was washed with 10 ml of lysis buffer and DscE was eluted by adding 5 ml of elution buffer (50 mM HEPES pH 7.8, 300 mM KCl, 0.05% (v/v) Triton, 10% glycerol and 100 mM imidazole) onto the column (eluate). The eluate was incubated with 1 ml of chitin resin (New England Biolabs) for 15 min at room temperature. After transferring the suspension onto an appropriate column, the flowthrough was collected (chitin eluate) and the buffer was exchanged with reconstitution buffer (50 mM HEPES pH 7.5, 300 mM KCl, 10% glycerol and 1 mM DTT) using a 30-kDa Amicon Ultra-0.5 centrifugal filter device (Merck) followed by a concentration step (concentrated and exchanged). Using all collected aliquots, a 12% SDS–PAGE was performed (Supplementary Fig. 19). The ultraviolet (UV)–visible light spectrum (λ = 200–100 nm) was recorded for the concentrated sample (Supplementary Fig. 20) and the protein concentration was measured using the UV absorbance at 280 nm and the calculated extinction coefficient ε.

#### Fe–S cluster reconstitution of DscE

The iron–sulfur clusters and cobalamin cofactors were reconstituted overnight at 4 °C. To approximately 127 mM DscE (one equivalent), 12 equivalents of l-cysteine, 13 equivalents of ammonium iron(II) sulfate, 20 equivalents of DTT, 2 equivalents of MeCbl, 1 mM IscS and 315 mM pyridoxalphosphate were added.

#### DscE in vitro assay

The reconstitution reaction was added to a 1-ml bed volume of TALON metal affinity resin (Takara) and incubated for 15 min at room temperature. After transferring the suspension onto an appropriate column, the flowthrough aliquot was collected. The sample was washed with 10 ml of lysis buffer and collected in three fractions (aliquots W1, W2 and W3). Proteins were eluted with 5 ml of elution buffer and collected in three fractions as well (aliquots E1, E2 and E3). Using these seven aliquots, SDS–PAGE analysis was performed using a 12% SDS–PAGE gel (Supplementary Fig. 19). As most of the DscE enzymes were in the flowthrough, this fraction was concentrated using an equilibrated 30-kDa Amicon Ultra-0.5 centrifugal filter device. Furthermore, the buffer was exchanged to reaction buffer (50 mM HEPES pH 7.5, 150 mM KCl and 10% glycerol). After this buffer exchange, the UV–visible light spectrum (λ = 200–100 nm) was recorded for the concentrated sample (Supplementary Fig. 20) and the protein concentration was measured using the UV absorbance at 280 nm. In vitro reactions were set up as follows in a total volume of 50 µl in reaction buffer and incubated overnight at room temperature: 20 mM DscE, 0.2 mM SAM, 0.2 mM methyl viologen, 0.2 mM NADPH, 0.2 mM substrate, 0.1 mM MeCbl and 2 mM DTT. The following controls were included: (1) heat-inactivated DscE instead of DscE; (2) DMSO instead of substrate; and (3) no addition of reduction system (methyl viologen, NADPH and DTT) (Supplementary Fig. 23). The next day, reactions were transferred out of the anaerobic chamber and quenched by adding 50 µl of 0.5 M formic acid in methanol. Samples were analyzed using HPLC–HRMS with the following parameters: solvent A, H_2_O + 0.1% formic acid; solvent B, CH_3_CN + 0.1% formic acid; column, Kinetex 2.6 mm XB-C18 100 Å (150 × 4.6 mm); flow rate, 1 ml min^−1^; column oven, 50 °C. The gradient was adjusted as follows: starting condition of 10% solvent B for 2 min, followed by a linear gradient over 10 min to 65% solvent B and an even steeper gradient over 1 min toward 98% solvent B. The column was further flushed with 98% solvent B for 3.5 min followed by a 0.4-min equilibration step to 10% solvent B. Before a new measurement, an equilibration step of 10% B over 3 min was performed. The MS instrument was operated in positive ionization mode at a scan range of 200–2,000 *m*/*z* and a resolution of 70,000. The spray voltage was set to 3.5 kV, S-lens was set to 50, sheath gas was set to 57.50, probe heater temperature was set to 462.50 °C and capillary temperature was set to 281.25 °C. The reaction was performed in triplicate and was repeated multiple times with freshly purified enzyme. Data analysis was conducted with Xcalibur 4.1 (Thermo Fisher).

#### Overproduction and purification of Cmb variants

The genes encoding CmbA homologs in ‘*Ca*. E. serta’ TSWB1 and ‘*Ca*. E. mitsugo’ TYSB3 were PCR-amplified from the synthetic genes (synthesized by Twist Bioscience; Supplementary Table 10) using the primers Cmb^Es^-F and Cmb^Es^-R or Cmb^Em^-F and Cmb^Em^-R, respectively (Supplementary Table 13). The PCR-amplified genes were analyzed on an agarose gel and the gene fragments were purified from there. Subsequent digestion with NdeI and HindIII and ligation into a pET28b(+) (Novagen) followed by introduction into *E*. *coli* DH5α resulted in the plasmids pET28b(+)-Cmb^Es^ and pET28b(+)-Cmb^Em^. The final plasmid constructs were then used to transform *E*. *coli* BL21 (DE3) (Stratagene).

*E*. *coli* BL21 (DE3) cells harboring pET28b(+)-Cmb^Es^ or pET28b(+)-Cmb^Em^ were precultured in LB medium containing 50 μg ml^−1^ kanamycin at 37 °C. The preculture was used to inoculate TB medium containing 50 μg ml^−1^ kanamycin and the cultures were grown at 37 °C for 2 h. Gene expression was then induced by the addition of IPTG at a final concentration of 0.1 mM and growth was continued at 18 °C for 14–16 h. The cells were harvested by centrifuging at 3,910*g* and 4 °C for 10 min. The cell pellets were resuspended in 50 mM Tris-HCl pH 8.0, 0.5 mM NaCl, 20 mM imidazole and 20% glycerol and cells were disrupted using a Branson Sonifier 250 (Emerson). The lysate was centrifuged at 34,700*g* at 4 °C for 10 min. The recombinant Cmb proteins were purified from the resulting supernatant using Ni-NTA Superflow resin (Qiagen). After washing with the buffer containing 50 mM Tris-HCl pH 8.0, 0.5 mM NaCl, 20 mM imidazole and 20% glycerol, the protein was eluted with 50 mM Tris-HCl pH 8.0, 0.5 mM NaCl, 250 mM imidazole and 20% glycerol. Finally, the protein was concentrated to an appropriate concentration with Vivaspin 10,000-kDa molecular weight cutoff.

#### Cmb in vitro assays

The standard assay was performed at 30 °C in a 100-μl reaction mixture containing 50 mM HEPES–NaOH pH 7.5, 2.0 mM MgCl_2_, 5.0 μM Cmb and 1.0 mM GGPP, GPP or FPP. The reaction was quenched by addition of 200 µl of ethyl acetate. After centrifugation, the upper layer was analyzed by GC–MS using a Shimadzu GCMS-QP2020. Sample introduction was performed by split injection onto a Shimadzu GLC SH-Rxi-5ms (5% diphenyl–95% dimethylpolysiloxane) column (30 m, 0.25-mm inner diameter, 0.25-µm film thickness). The injector temperature was 230 °C. The initial column temperature was 50 °C and this temperature was held for 1 min after injection. Next, the temperature was increased to 150 °C at 10 °C min^−1^ and then to 280 °C at 20 °C min^−1^. The temperature was held at 280 °C for the remainder of the 22.5-min program. Data analysis was conducted with LabSolutions CS (version 4.42).

#### Isolation and structure elucidation of cembrene A (21)

To obtain **21**, the Cmb^Es^ assay was performed in a 50-ml reaction mixture containing 50 mM HEPES–NaOH pH 7.5, 0.5 mM GGPP, 2.0 mM MgCl_2_ and 2.9 μM Cmb^Es^. The reactions were incubated at 30 °C overnight and extracted with hexane (2 × 100 ml) and ethyl acetate (2 × 100 ml). The combined organic layer was dried with MgSO_4_, concentrated under reduced pressure and the target diterpene was purified by column chromatography on silica gel with *n*-hexane to yield cembrene A (**1**, 0.8 mg). NMR spectra were recorded on a JEOL ECA-600 spectrometer operating at 600 MHz for ^1^H and 150 MHz for ^13^C nuclei. NMR data were analyzed using Delta 5.3 (JEOL). The optical rotations were recorded with a P-2100 polarimeter (JASCO) and compared to the reported values for (*R*)-cembrene (−12) (ref. ^87^) and (*S*)-cembrene (+19.5) (ref. ^88^).

### Reporting summary

Further information on research design is available in the Nature Portfolio Reporting Summary linked to this article.

## Online content

Any methods, additional references, Nature Portfolio reporting summaries, source data, extended data, supplementary information, acknowledgements, peer review information; details of author contributions and competing interests; and statements of data and code availability are available at 10.1038/s41589-025-02066-0.

## Supplementary information

Supplementary Information Supplementary Note, Figs. 1–34, Tables 1–4 and 6–13 and References.

Reporting Summary

Supplementary Table 5 Summary of additional ‘Tectomicrobia’ members found in the mOTUs database.

## Figures and Tables

**Extended Data Table 1:**
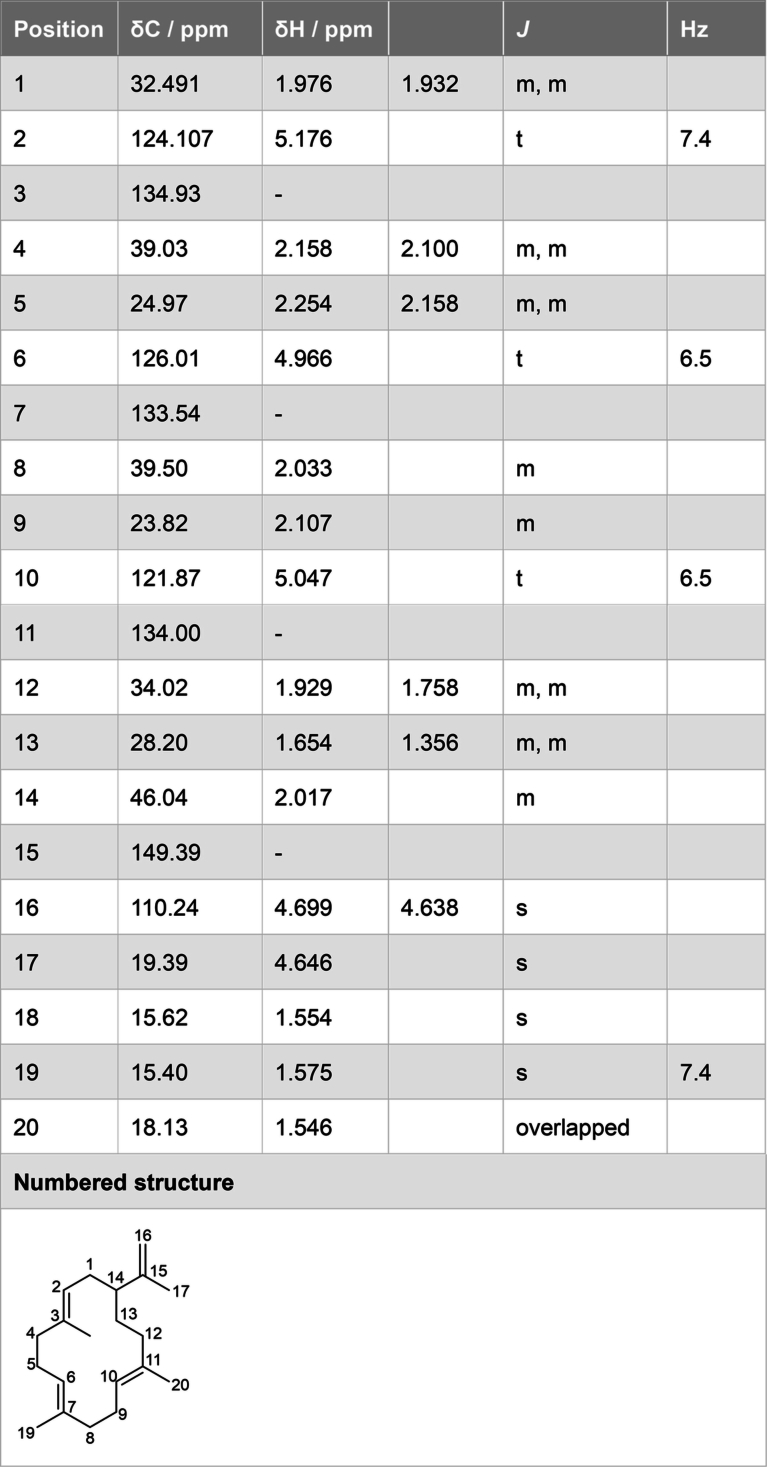
NMR data for structure elucidation of compound 21

^13^C and ^1^H NMR spectral data for structure elucidation of compound **21**. (^1^H-NMR, 600 MHz; ^13^C-NMR, 150 MHz; chloroform-d_6_).

**Extended Data Table 2:**
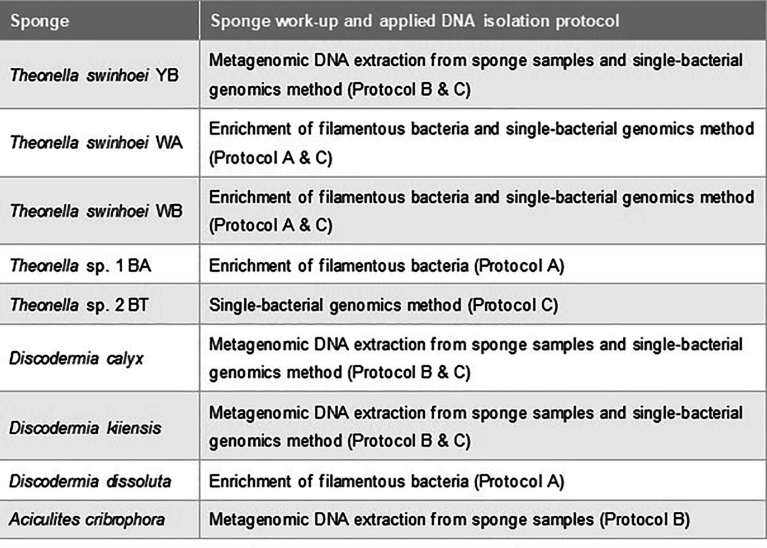
Summary of methods on sponge sample processing

DNA isolation and prior work-up of sponge samples.

## Data Availability

All data supporting the findings of this study are available within the main text and the Supplementary Information. DNA sequences were deposited to the European Nucleotide Archive under BioProjects PRJEB80215 (all except for ‘*Ca*. P. opulenta’ AC1) and PRJEB59408 (‘*Ca*. P. opulenta’ AC1) with the following accession numbers: ‘*Ca*. E. symbiotica’ BT01, GCA_964656635; ‘*Ca*. E. inquilina’ BT02, GCA_964656685; ‘*Ca*. E. melakyensis’ BT03, GCA_964656765; ‘*Ca*. E. catenata’ BT04, GCA_964656715; ‘*Ca*. E. armillaria’ DC1, GCA_964656755; ‘*Ca*. E. tacita’ DD1, GCA_964656785; ‘*Ca*. E. baccata’ DD2, GCA_964656735; ‘*Ca*. E. tertia’ DD3, GCA_964656775; ‘*Ca*. E. monilis’ DK1, GCA_964656725; ‘*Ca*. E. melakyensis’ TCBA1, GCA_964656645; ‘*Ca*. E. serta’ TCBA2, GCA_964656625; ‘*Ca*. E. serta’ TSWA1, GCA_964656745; ‘*Ca*. E. serta’ TSWB1, GCA_964656705; ‘*Ca*. E. consors’ TSWB2, GCA_964656675; ‘*Ca*. E. factor’ TSYB1, GCA_964656695; ‘*Ca*. E. gemina’ TSYB2, GCA_964656665; ‘*Ca*. E. mitsugo’ TSYB3, GCA_964656655; ‘*Ca*. P. opulenta’ AC1, GCA_965178525. The *dsc* BGC was deposited to MIBiG with accession number BGC0003182. Other data related to this work (for example, HPLC–HRMS) are available from the lead contact upon request.
