# Supplementary material for: Chemical richness and diversity of uncultivated ‘Entotheonella’ symbionts in marine sponges
Source: Nat Chem Biol. 2025 Nov 13;22(2):217–28. doi: 10.1038/s41589-025-02066-0 (PMC12858407; doi:10.1038/s41589-025-02066-0)
Supplement: Supplementary file 1 — Supplementary Note, Figs. 1–34, Tables 1–4 and 6–13 and References. [file 41589_2025_2066_MOESM1_ESM.pdf]

nature chemical biology

Supplementary information https://doi.org/10.1038/s41589-025-02066-0

Chemical richness and diversity of uncultivated ‘Entotheonella’ symbionts in marine sponges

In the format provided by the authors and unedited

Table of contents

Supplementary Note 3
Supplementary Figure 1 5
Supplementary Figure 2 6
Supplementary Figure 3 7
Supplementary Figure 4 8
Supplementary Figure 5 9
Supplementary Figure 6 10
Supplementary Figure 7 11
Supplementary Figure 8 12
Supplementary Figure 9 13
Supplementary Figure 10 14
Supplementary Figure 11 15
Supplementary Figure 12 16
Supplementary Figure 13 17
Supplementary Figure 14 18
Supplementary Figure 15 19
Supplementary Figure 16 20
Supplementary Figure 17 21
Supplementary Figure 18 22
Supplementary Figure 19 23
Supplementary Figure 20 24
Supplementary Figure 21 25
Supplementary Figure 22 26
Supplementary Figure 23 27
Supplementary Figure 24 28
Supplementary Figure 25 29
Supplementary Figure 26 30
Supplementary Figure 27 31
Supplementary Figure 28 32
Supplementary Figure 29 33
Supplementary Figure 30 34
Supplementary Figure 31 35
Supplementary Figure 32 36
Supplementary Figure 33 37
Supplementary Figure 34 38
Supplementary Table 1 40
Supplementary Table 2 41
Supplementary Table 3 42
Supplementary Table 4 43
Supplementary Table 5 44

Supplementary Table 6 45
Supplementary Table 7 46
Supplementary Table 8 47
Supplementary Table 9 48
Supplementary Table 10 49
Supplementary Table 11 51
Supplementary Table 12 52
Supplementary Table 13 54
Supplementary References 55

Supplementary Note

Composition of branches that are collapsed to group 1: *Marinomonas spartinae* CECT 8886, *Photobacterium piscicola* NCCB 100098, *Vibrio toranzoniae* CECT 7225, *Ferrimonas balearica* DSM 9799, *Verminephrobacter eiseniae* EF01-2, *Pseudomonas lactis* DSM 29167, *Pseudomonas* sp. TKP, *Pseudomonas paralactis* DSM 29164, *Pseudomonas flexibilis* JCM 14085, *Derxia lacustris* HL-12, *Rubrobacter xylanophilus* DSM 9941. Composition of branches that are collapsed to group 2: *Streptomyces sporocinereus* NBRC 100766, *Streptomyces hokutonensis* R1-NS-10, *Streptomyces mobaraensis* NBRC13819; DSM 40847, *Streptomyces decoyicus* NRRL 2666, *Streptomyces niger* NRRL B-3857, *Mycobacterium septicum* DSM 44393, *Nocardia gamkensis* NBRC 108242, *Lentzea kentuckyensis* NRRL B-24416, *Leucobacter musarum* subsp. *musarum* CBX152, *Leucobacter celer* subsp. *astrifaciens* CBX151, *Loktanella soesokkakensis* CECT 8367, *Roseovarius albus* CECT 7450, *Methylobacterium nodulans* ORS 2060, *Nitratireductor pacificus* pht-3B, *Sphingomonas echinoides* ATCC 14820. All these genomes were automatically chosen during the de novo mode analysis with autoMLST. [1]

Supplementary Figures

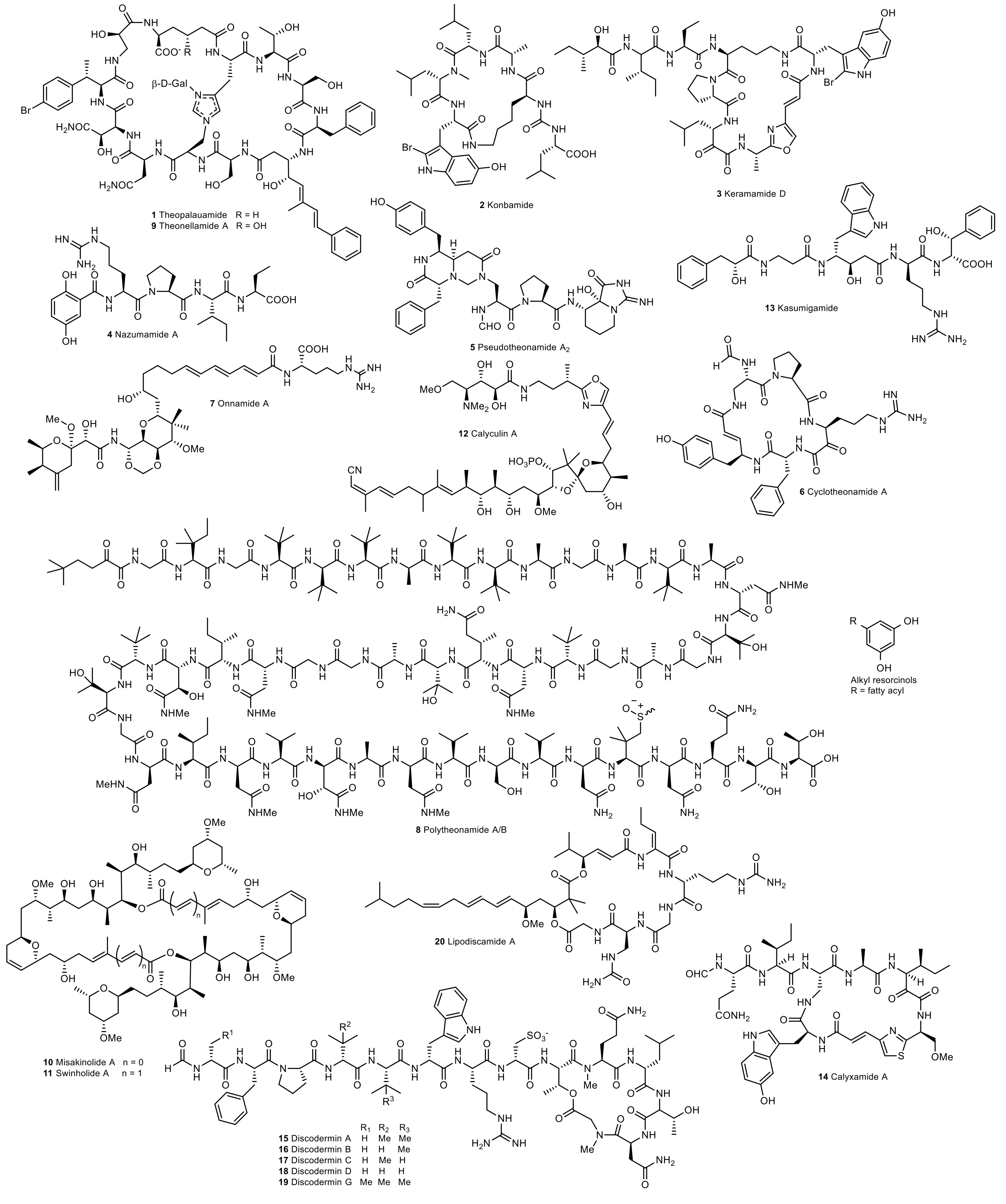

Supplementary Fig. 1: Sponge-derived natural products relevant to this study.

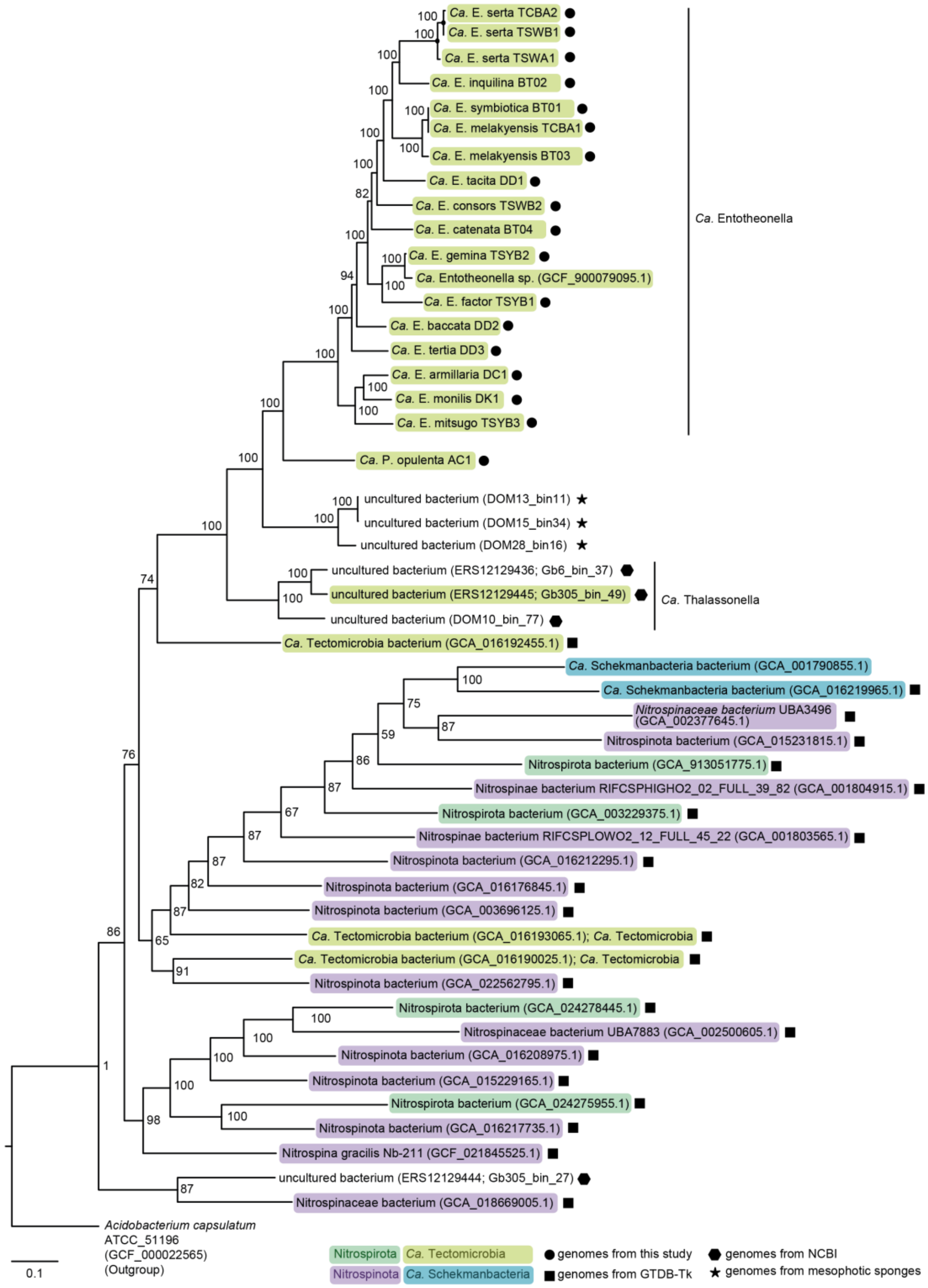

Supplementary Fig. 2: Phylogenomic analysis of '*Ca.* Entotheonella' and '*Ca.* Proxinella'. Analysis includes genomes from this study, their closest organisms identified with GTDB-TK[2], and four additional bins found in the metagenomes of DOM sponges from Dominica. The tree was generated using a local copy of autoMLST[1] and subsequently rooted with autoMLST's underlying algorithm. With this analysis we compared the symbiont genome identified in the sponge *Aciculites cribrophora* to specific organisms of interest (the closest ones identified with GTDB-TK) rather than the genomes that are automatically chosen in the *de novo* mode of autoMLST[1]. Based on this analysis and the ANI values in Supplementary Fig. 3, '*Ca.* P. opulenta' AC1 potentially belongs to a new candidate genus within 'Tectomicrobia'.

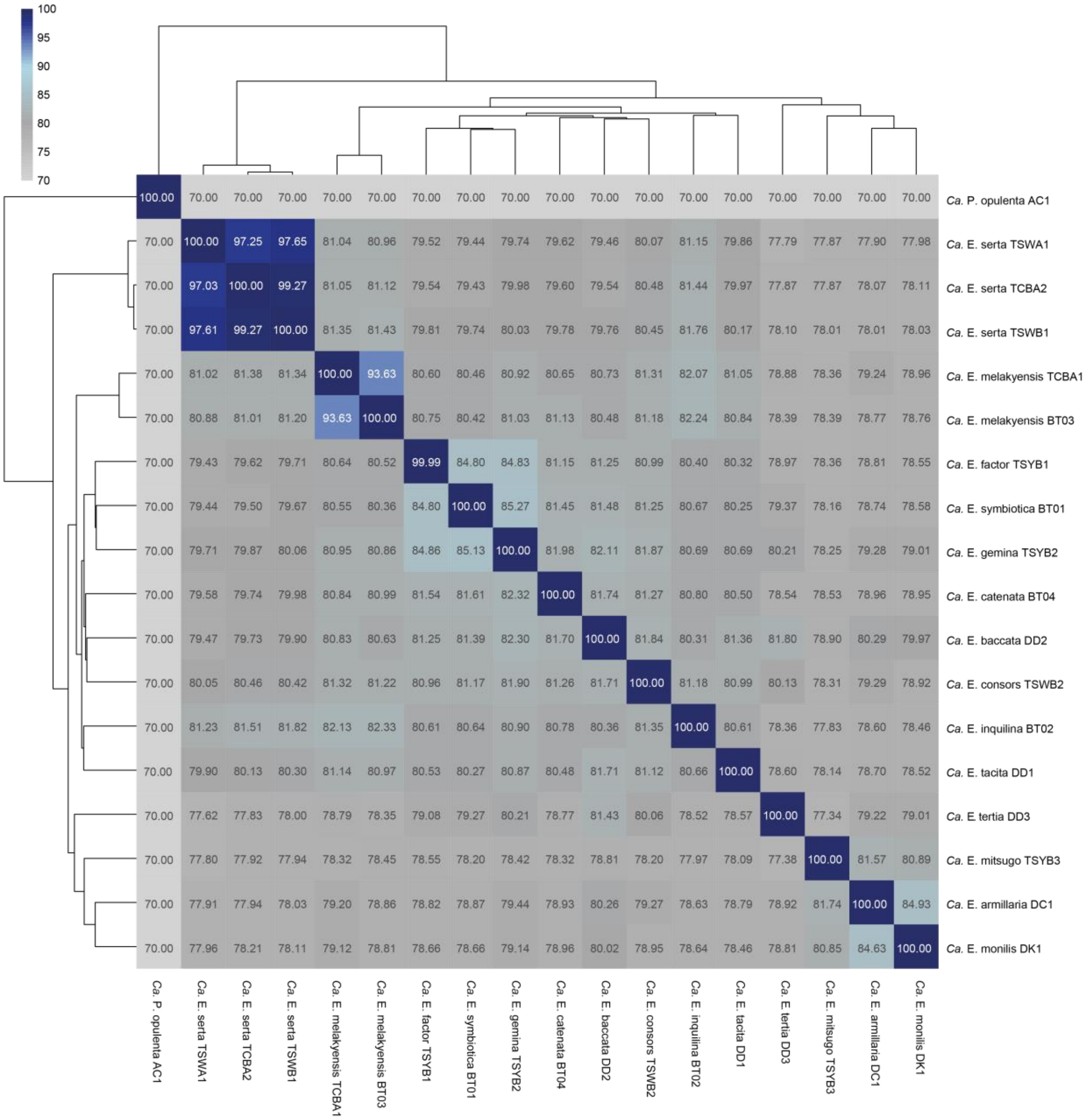

Supplementary Fig. 3: Matrix of calculated fastANI values. The matrix was prepared using the fastANI tool[3] with the method all-vs-all. The values represent the percentage of shared average nucleotide identities.

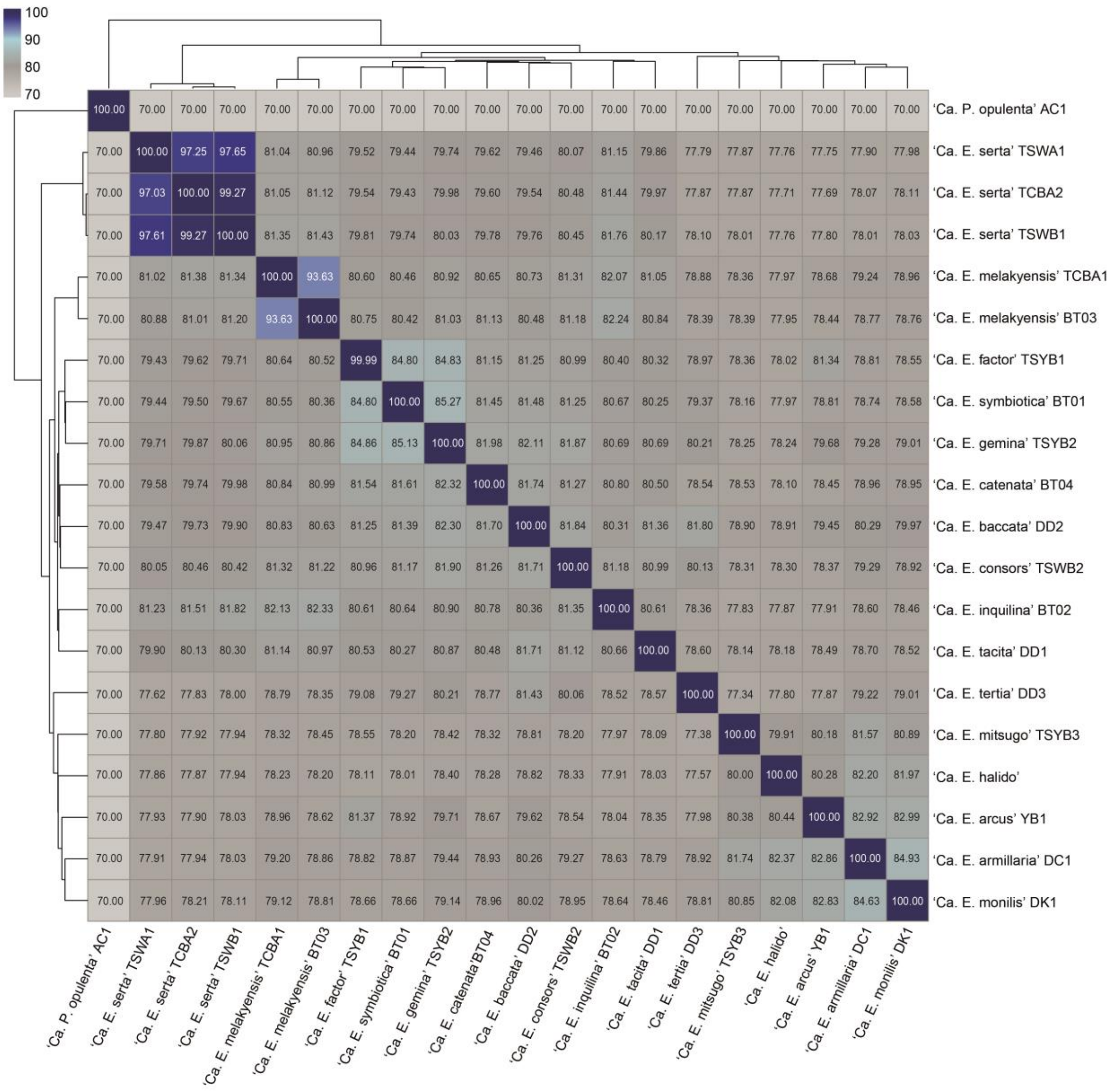

Supplementary Fig. 4: Matrix of calculated fastANI values including the genome of '*Ca.* E. arcus' Y-B1 and '*Ca.* E. halido'. The matrix was prepared using the fastANI tool[3] with the method all-vs-all. The values represent the percentage of shared average nucleotide identities.

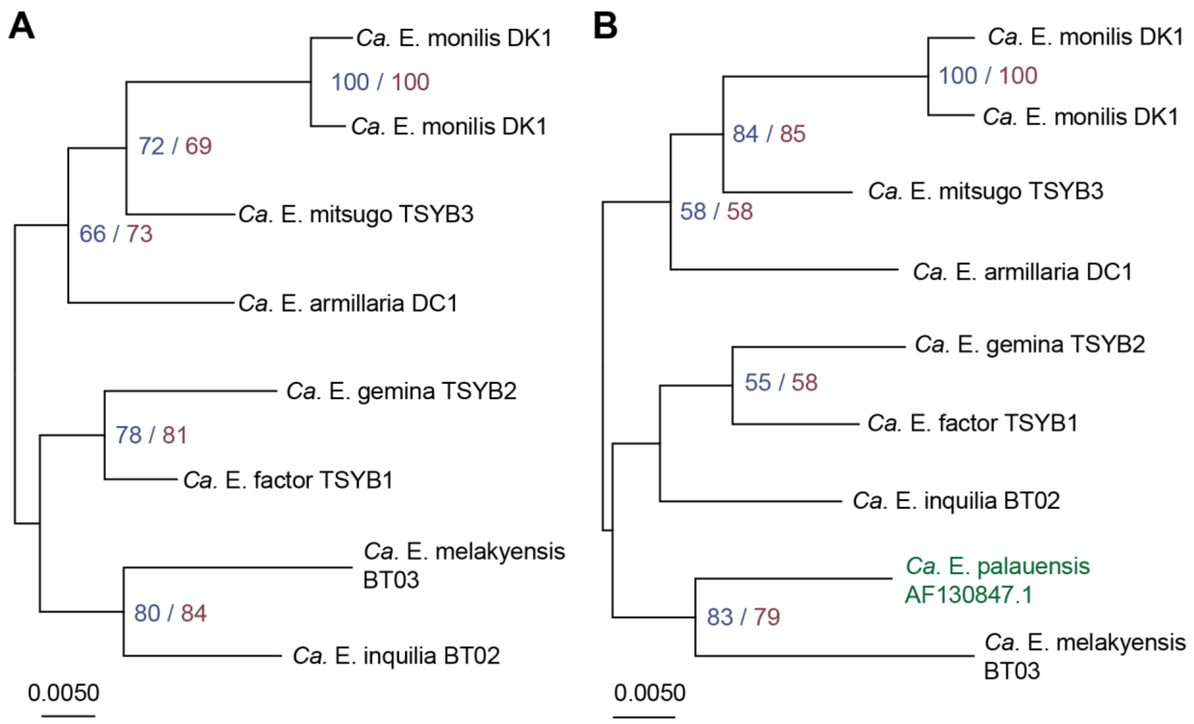

Supplementary Fig. 5: 16S rRNA phylogenetic analysis of tectomicrobial symbionts identified in this study (A) and compared to '*Ca.* E. palauensis' (B, written in green font). Phylogenetic trees were generated based on a MUSCLE alignment (generated with Geneious 8) of full-length 16S rRNA genes. Trees were generated using the Maximum Likelihood (ML) and the Neighbor-Joining (NJ) algorithms. Shown are the branches that resulted from the ML method. If the same branches appeared in the NJ calculations, the nodes show bootstrap values in blue for the ML tree and in red for the NJ tree. The ML method is based on the General Time Reversible model[4]. The tree with the highest log likelihood (-3363.8384 (**A**) and (-3114.0780) (**B**)) is shown. The percentage of trees in which the associated taxa clustered together is shown next to the branches. Initial tree(s) for the heuristic search were obtained automatically by applying NJ and BioNJ algorithms to a matrix of pairwise distances estimated using the Maximum Composite Likelihood (MCL) approach, and then selecting the topology with superior log likelihood value. The tree is drawn to scale, with branch lengths measured in the number of substitutions per site (next to the branches). When evolutionary history was inferred using the NJ method[5] and the branch appearance was in accordance to the ML algorithm, the percentage of replicate trees in which the associated taxa clustered together in the bootstrap test (500 replicates) are shown next to the branches[6]. The tree is drawn to scale, with branch lengths shown in the scale bar. The evolutionary distances were computed using the p-distance method[4] and are in the units of the number of base differences per site. The analysis involved 8 (**A**) or 9 (**B**) nucleotide sequences. All ambiguous positions were removed for each sequence pair. A total of 1524 (**A**) or 1336 (**B**) positions were in the final dataset. Evolutionary analyses were conducted in MEGA7[7].

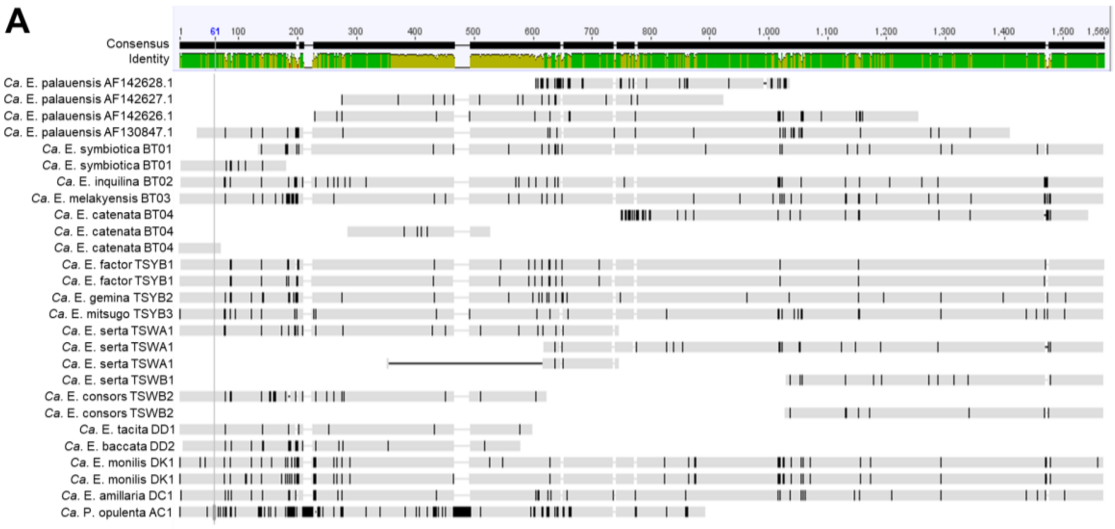

B

	Ca. E. palauensis AF142628.1	Ca. E. palauensis AF142627.1	Ca. E. palauensis AF142626.1	Ca. E. palauensis AF130847.1	Ca. E. symbiotica BT01	Ca. E. symbiotica BT01	Ca. E. inquiliana BT02	Ca. E. melakyensis BT03	Ca. E. catenata BT044	Ca. E. catenata BT044	Ca. E. catenata BT04	Ca. E. factor TSYB1	Ca. E. factor TSYB1	Ca. E. gemina TSYB2	Ca. E. mitsugo TSY3	Ca. E. serta TSWA1	Ca. E. serta TSWA1	Ca. E. serta TSWA1	Ca. E. serta TSWB1	Ca. E. consors TSWB2	Ca. E. consors TSWB2	Ca. E. tacita DD1	Ca. E. baccata DD2	Ca. E. monilis DK1	Ca. E. monilis DK1	Ca. E. armillaria DC1	Ca. P. opulenta AC1
Ca. E. palauensis AF142628.1		89.24	90.19	88.61	89.49	0.00	89.95	89.25	87.68	0.00	0.00	89.02	89.02	89.02	89.49	86.33	90.10	80.58	100	66.67	83.33	0.00	0.00	88.79	88.79	88.32	90.88
Ca. E. palauensis AF142627.1			97.00	97.33	97.49	0.00	96.68	97.65	88.08	95.79	0.00	97.16	97.16	96.92	97.00	97.39	97.51	35.17	0.00	96.88	0.00	97.97	96.73	96.84	97.33	96.35	88.56
Ca. E. palauensis AF142626.1				96.79	96.08	0.00	96.08	96.08	92.43	96.73	0.00	96.98	96.98	96.18	97.89	96.10	95.40	34.62	94.17	96.73	94.20	97.66	97.21	97.18	97.28	97.08	88.92
Ca. E. palauensis AF130847.1					96.34	93.38	95.96	95.96	94.38	97.66	100	97.08	97.08	96.70	96.48	95.98	95.94	34.52	95.24	94.92	96.57	96.96	96.25	95.96	95.96	95.81	84.09
Ca. E. symbiotica BT01						100	96.11	96.10	94.30	96.73	0.00	97.76	97.76	97.40	96.53	95.58	97.24	34.89	96.63	94.17	98.13	97.15	95.26	95.67	95.88	96.03	83.82
Ca. E. symbiotica BT01							96.07	93.82	0.00	0.00	100	96.63	96.63	95.51	94.38	94.94	0.00	34.89	0.00	94.38	0.00	95.51	94.86	92.70	93.82	94.94	89.89
Ca. E. inquiliana BT02								96.25	94.68	96.73	100	96.45	96.45	95.73	96.12	95.99	97.04	0.00	96.65	95.51	96.65	96.39	95.48	95.59	95.66	95.92	84.76
Ca. E. melakyensis BT03									94.05	96.73	100	96.31	96.31	95.99	95.33	96.14	96.82	35.17	97.00	94.66	96.82	96.76	93.97	95.26	95.33	95.19	84.91
Ca. E. catenata BT04										0.00	0.00	95.44	95.44	95.06	93.42	0.00	94.18	35.17	96.67	0.00	97.46	0.00	0.00	93.29	93.29	94.05	80.14
Ca. E. catenata BT04											0.00	97.20	97.20	97.20	96.73	96.26	0.00	3.38	0.00	96.73	0.00	97.20	96.73	97.20	96.73	97.20	81.25
Ca. E. catenata BT04												100	100	100	98.57	100	0.00	0.00	0.00	100	0.00	100	100	95.71	98.57	98.55	95.71
Ca. E. factor TSYB1													100	97.69	96.84	95.13	97.35	34.07	97.57	94.81	98.51	97.11	95.85	96.18	96.24	96.90	85.33
Ca. E. factor TSYB1														97.69	96.84	95.14	97.35	34.07	97.57	94.82	98.51	97.11	95.85	96.18	96.25	96.90	85.34
Ca. E. gemina TSYB2															96.38	95.28	96.71	34.34	96.63	94.65	97.76	96.58	96.23	95.26	95.33	96.77	84.91
Ca. E. mitsugo TSY3																95.57	96.61	34.89	95.88	93.78	96.45	96.40	95.47	96.71	96.78	97.17	85.71
Ca. E. serta TSWA1																	100	35.99	0.00	95.86	0.00	96.93	96.60	94.00	94.00	95.28	83.67
Ca. E. serta TSWA1																		100	97.38	100.00	97.01	0.00	0.00	96.07	96.18	96.39	93.33
Ca. E. serta TSWA1																			0.00	4.51	0.00	2.28	2.01	35.17	35.17	34.34	31.03
Ca. E. serta TSWB1																				0.00	96.82	0.00	0.00	96.44	96.63	96.26	0.00
Ca. E. consors TSWB2																					0.00	95.13	95.09	93.10	93.10	95.34	81.64
Ca. E. consors TSWB2																						0.00	0.00	96.45	96.64	96.45	0.00
Ca. E. tacita DD1																							95.66	94.78	94.78	95.86	82.41
Ca. E. baccata DD2																								94.16	94.54	96.04	81.29
Ca. E. monilis DK1																									99.28	96.05	84.48
Ca. E. monilis DK1																										96.12	84.70
Ca. E. armillaria DC1																											85.02
Ca. P. opulenta AC1																											

Supplementary Fig. 6: Comparison of identified 16S rRNA genes. A, MUSCLE Alignment of all identified 16S rRNA genes (see Experimental procedures) using Geneious 8. **B**, Table of pairwise sequence identities calculated from the alignment. Values greater than 97% are highlighted in grey.

	Ca. E. melakyensis TCBA1	*Ca.* E. serta TCBA2	*Ca.* E. serta TSWA1	*Ca.* E. serta TSWB1	*Ca.* E. consors TSWB2	*Ca.* E. symbiotica BT01	*Ca.* E. inquiliana BT02	*Ca.* E. melakyensis BT03	*Ca.* E. catenata BT04	*Ca.* E. factor TSYB1	*Ca.* E. gemina TSYB2	*Ca.* E. mitsugo TSYB3	*Ca.* E. tacita DD1	*Ca.* E. baccata DD2	*Ca.* E. tertia DD3	*Ca.* E. armillaria DC1	*Ca.* E. monilis DK1	*Ca.* P. opulenta AC1	MiBiG
Ca. E. melakyensis TCBA1		3	3	3	3	4	2	3	3	3	4	4	3	2	2	2	1	2	0
Ca. E. serta TCBA2			7	22	2	2	1	2	2	1	1	1	2	2	1	1	0	1	4
Ca. E. serta TSWA1				14	1	2	1	1	1	1	2	2	1	0	0	1	1	1	4
Ca. E. serta TSWB1					1	2	2	3	2	2	3	3	3	1	0	2	1	1	4
Ca. E. consors TSWB2						1	2	1	2	3	3	3	2	2	0	3	1	1	1
Ca. E. symbiotica BT01							5	1	0	1	3	2	1	3	1	2	2	0	0
Ca. E. inquiliana BT02								2	0	1	4	4	3	2	0	2	2	2	1
Ca. E. melakyensis BT03									2	2	2	1	2	1	0	0	0	2	0
Ca. E. catenata BT04										1	1	0	2	2	0	0	0	0	0
Ca. E. factor TSYB1											4	3	2	0	1	0	0	2	1
Ca. E. gemina TSYB2												2	3	2	1	2	0	3	2
Ca. E. mitsugo TSYB3													1	1	0	1	2	0	0
Ca. E. tacita DD1														1	0	0	1	2	1
Ca. E. baccata DD2															0	0	1	2	1
Ca. E. tertia DD3																1	1	0	0
Ca. E. armillaria DC1																	3	2	1
Ca. E. monilis DK1																		0	1
Ca. P. opulenta AC1																			0

Supplementary Fig. 7: Number of shared BGCs between symbiont genomes as identified with BiG-SCAPE. The numbers were calculated based on the BiG-SCAPE output.

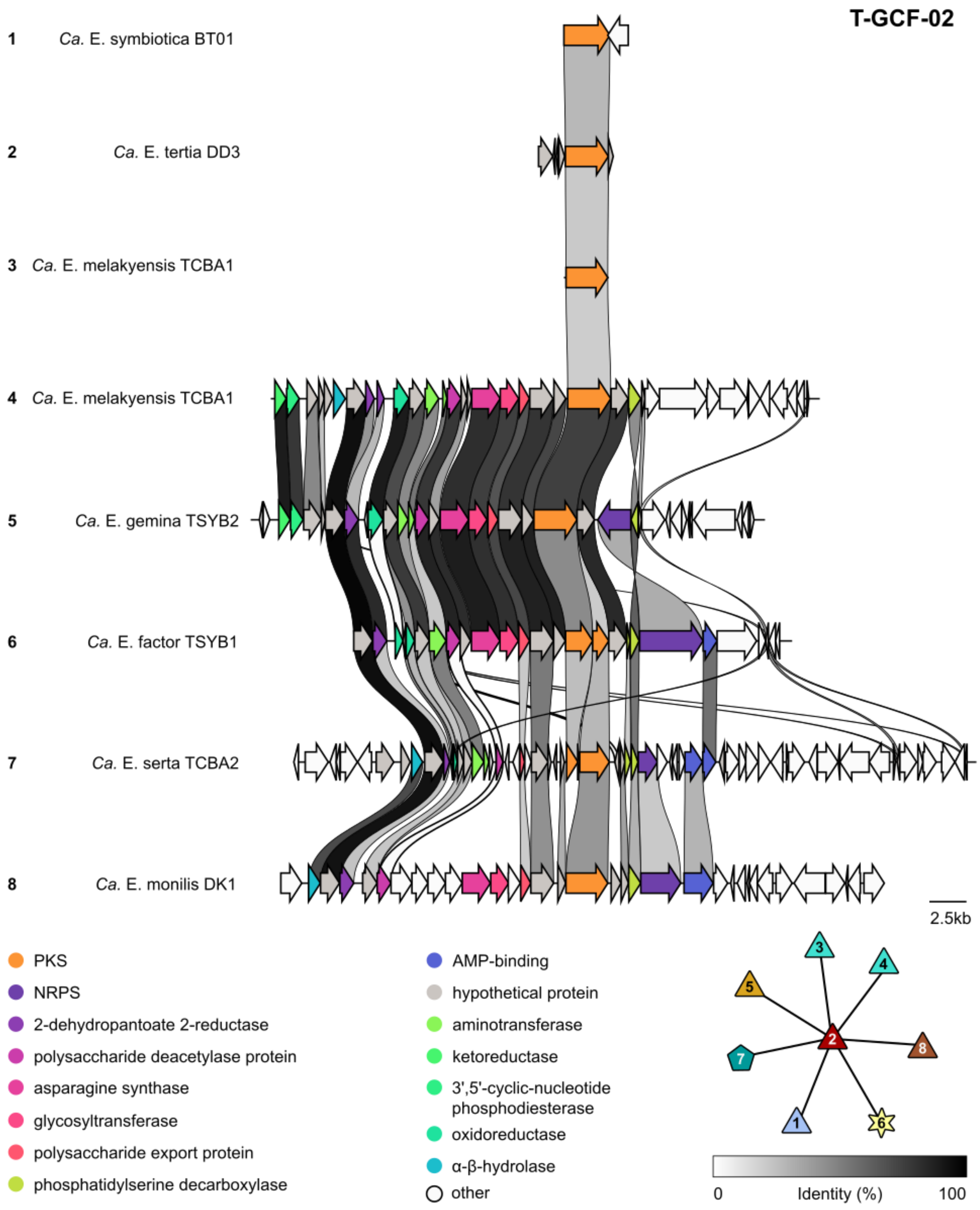

Supplementary Fig. 8: Comparison of BGCs assigned to the Gene Cluster Family T-GCF-02. The BGCs form a sub-network (bottom right to the figure) that is part of the BGC network analysis shown in Fig. 3. The comparison was done using clinker[8] with a cutoff of 0.3. The orange gene that is shared among all BGCs encodes a PKS. Multiple genes in the middle of the BGC are related to each other. 8 out of 18 genomes shared this BGC. Shared genes are connected by black/grey shades.

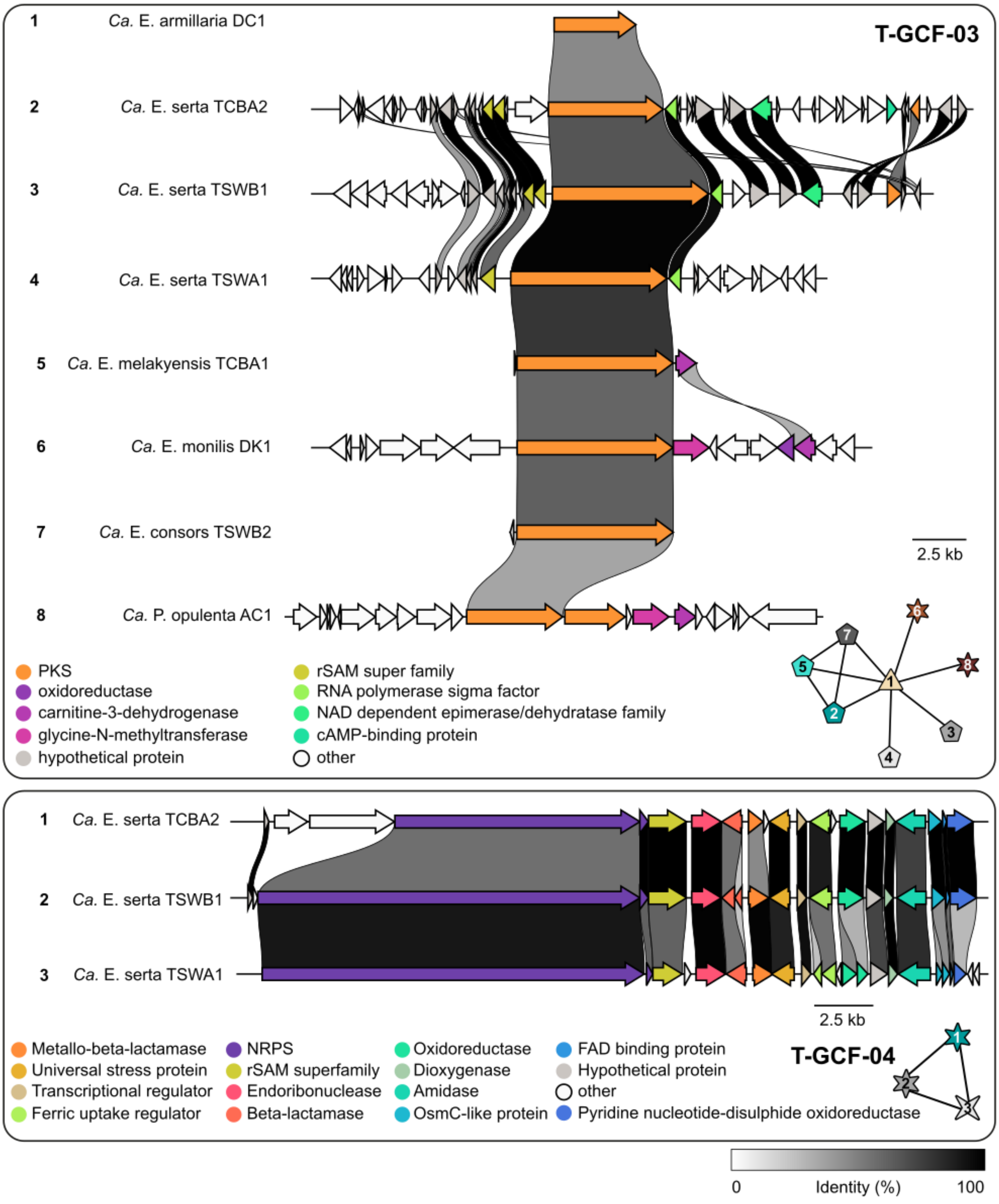

Supplementary Fig. 9: Comparison of BGCs assigned to the Gene Cluster Families T-GCF-03 and T-GCF-04. The BGCs form sub-networks (bottom right to the BGCs) that are part of the BGC network analysis shown in Fig. 3. The comparison was done using clinker[8] with a cutoff of 0.3. In T-GCF-03 central to the similar genes is a gene encoding a PKS with an N-terminal TE-domain and an adenylation domain. In T-GCF-04 the central gene encoding an NRPS and all the genes encoding putative tailoring enzymes are similar to each other in the three genomes of ‘*Ca.* E. serta’. Shared genes are connected by black/grey shades.

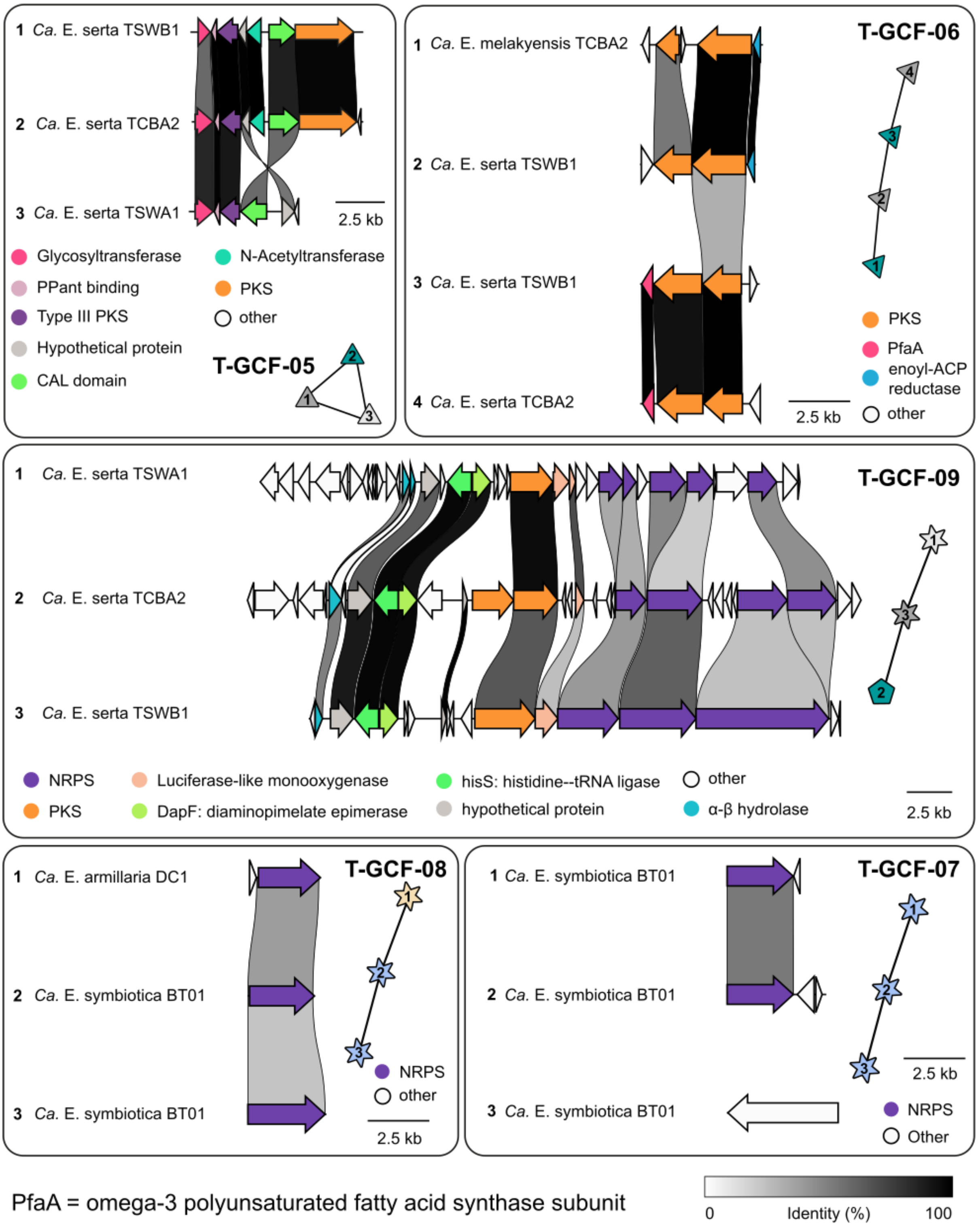

Supplementary Fig. 10: Comparison of BGCs assigned to the Gene Cluster Families T-GCF-05, T-GCF-06, T-GCF-07, T-GCF-08, and T-GCF-09. The BGCs form sub-networks (right side within the boxes) that are part of the BGC network analysis shown in Fig. 3. The comparison was done using clinker[8] with a cutoff of 0.3. Shared genes are connected by black/grey shades.

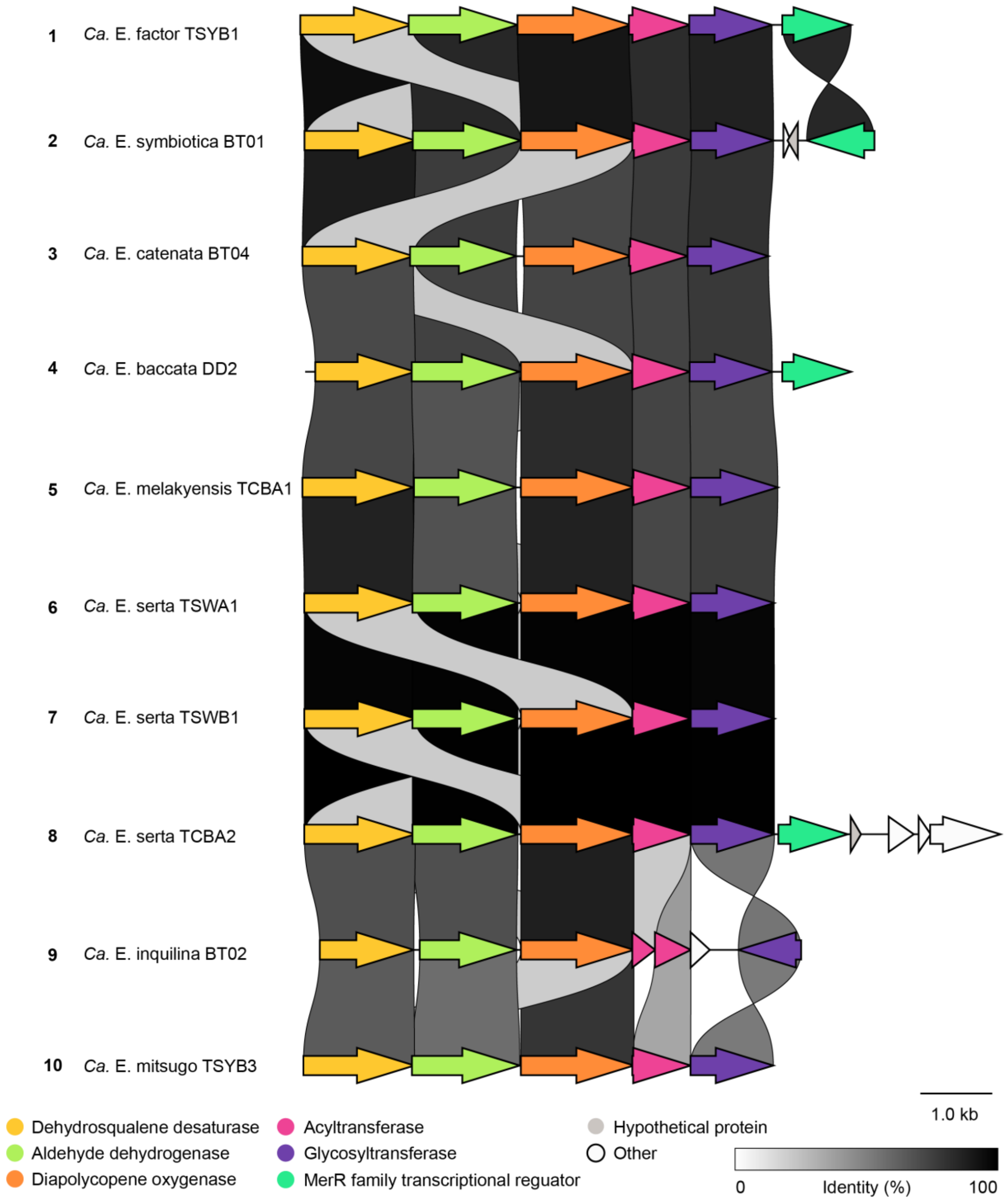

Supplementary Fig. 11: Comparison of BGCs putatively encoding theoxanthin biosynthesis. The genes were manually identified based on a previously identified genes encoding the biosynthesis for theoxanthin in '*Ca.* Entotheonella' bacteria[9]. The co-occurrence of this set of genes was identified in ten different genomes and compared using clinker[8] with a cutoff of 0.3. Shared genes are connected by black/grey shades.

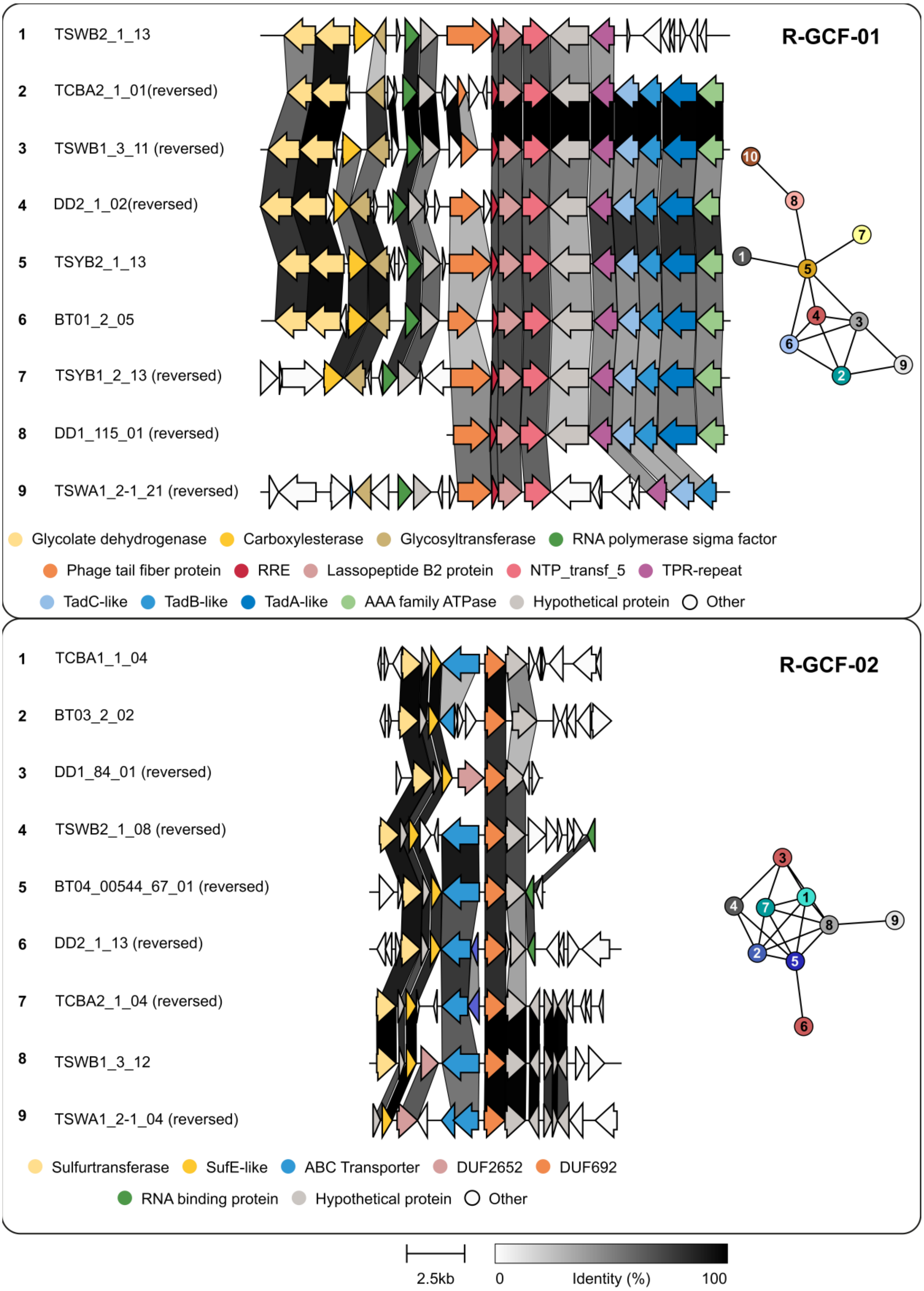

Supplementary Fig. 12: Comparison of BGCs in the RiPP group of biosynthetic families (R-GCF-01 to R-GCF-02). The BGCs form sub-networks (right side within the boxes) that are part of the BGC network analysis shown in Fig. 3. Shown are the BGCs in R-GCF-1 (top) and R-GCF-2 (bottom) using clinker[8] with a cutoff of 0.3. Shared genes are connected by black/grey shades.

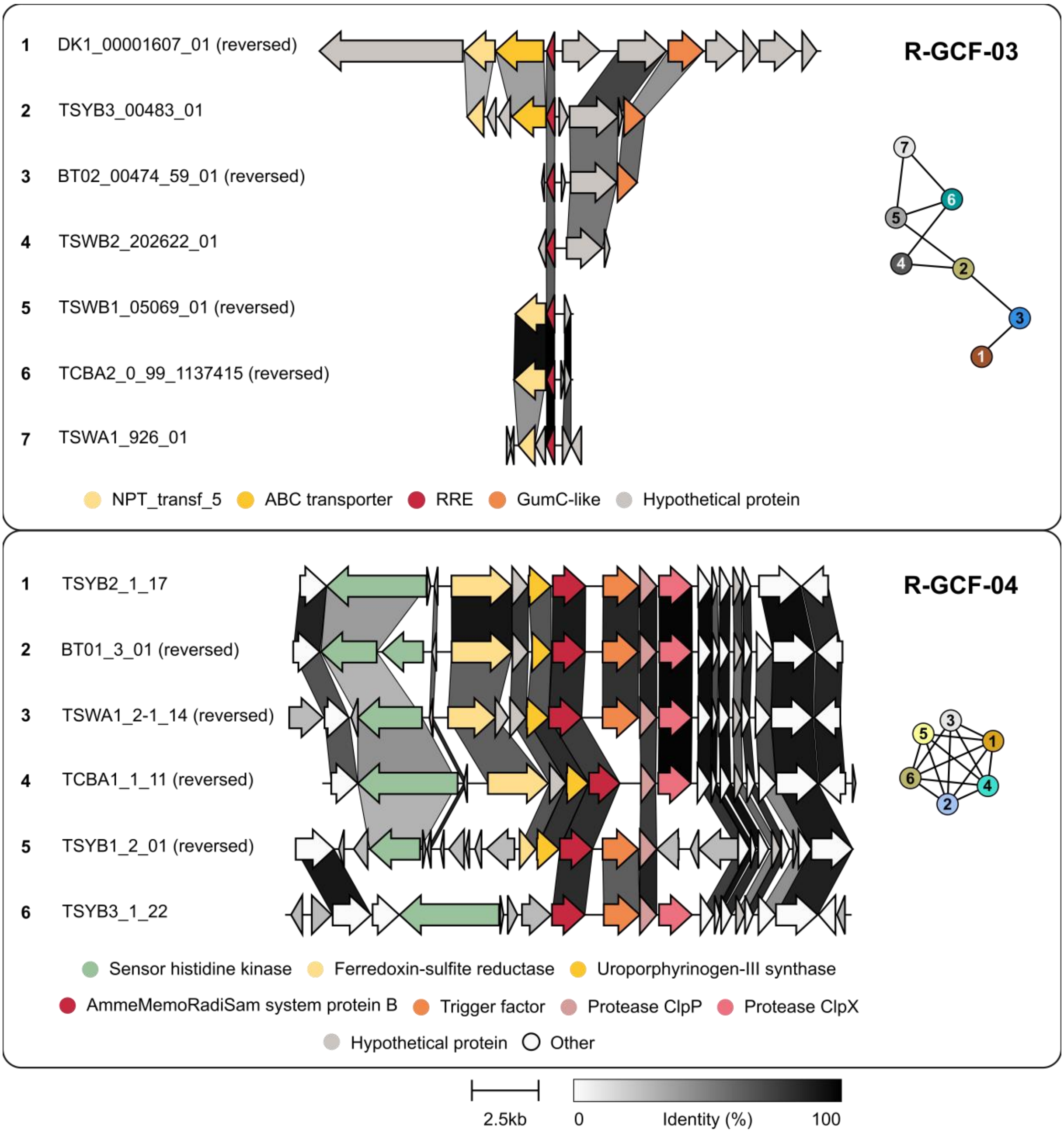

Supplementary Fig. 13: Comparison of BGCs in the RiPP group of biosynthetic families (R-GCF-03 to R-GCF-04). The BGCs form sub-networks (right side within the boxes) that are part of the BGC network analysis shown in Fig. 3. Shown are the BGCs in R-GCF-3 (top) and R-GCF-4 (bottom) using clinker[8] with a cutoff of 0.3. Shared genes are connected by black/grey shades.

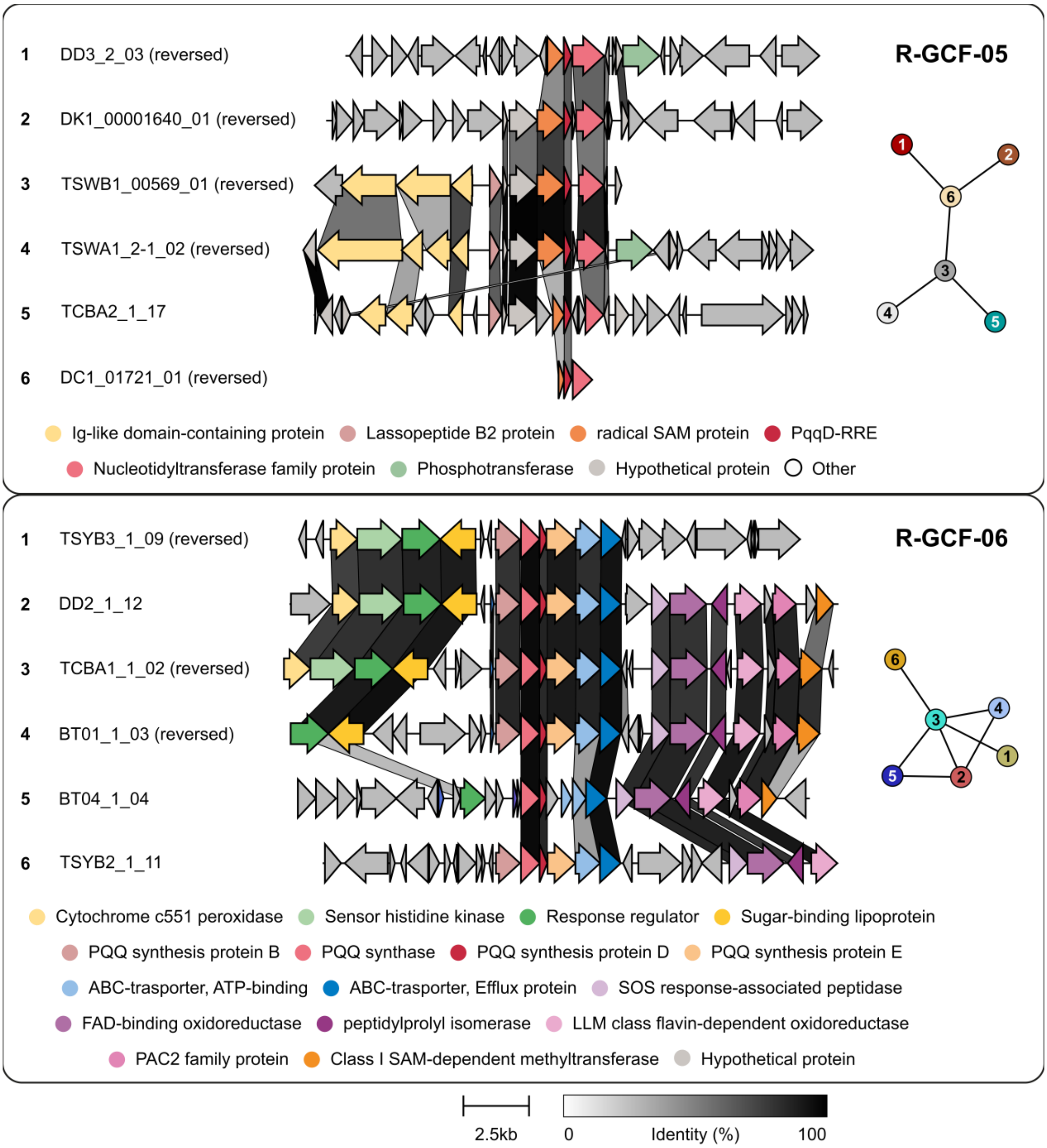

Supplementary Fig. 14: Comparison of BGCs in the RiPP biosynthetic families (R-GCF-05 to R-GCF-06). The BGCs form sub-networks (right side within the boxes) that are part of the BGC network analysis shown in Fig. 3. Shown are the BGCs in R-GCF-5 (top) and R-GCF-6 (bottom) using clinker[8] with a cutoff of 0.3. Shared genes are connected by black/grey shades.

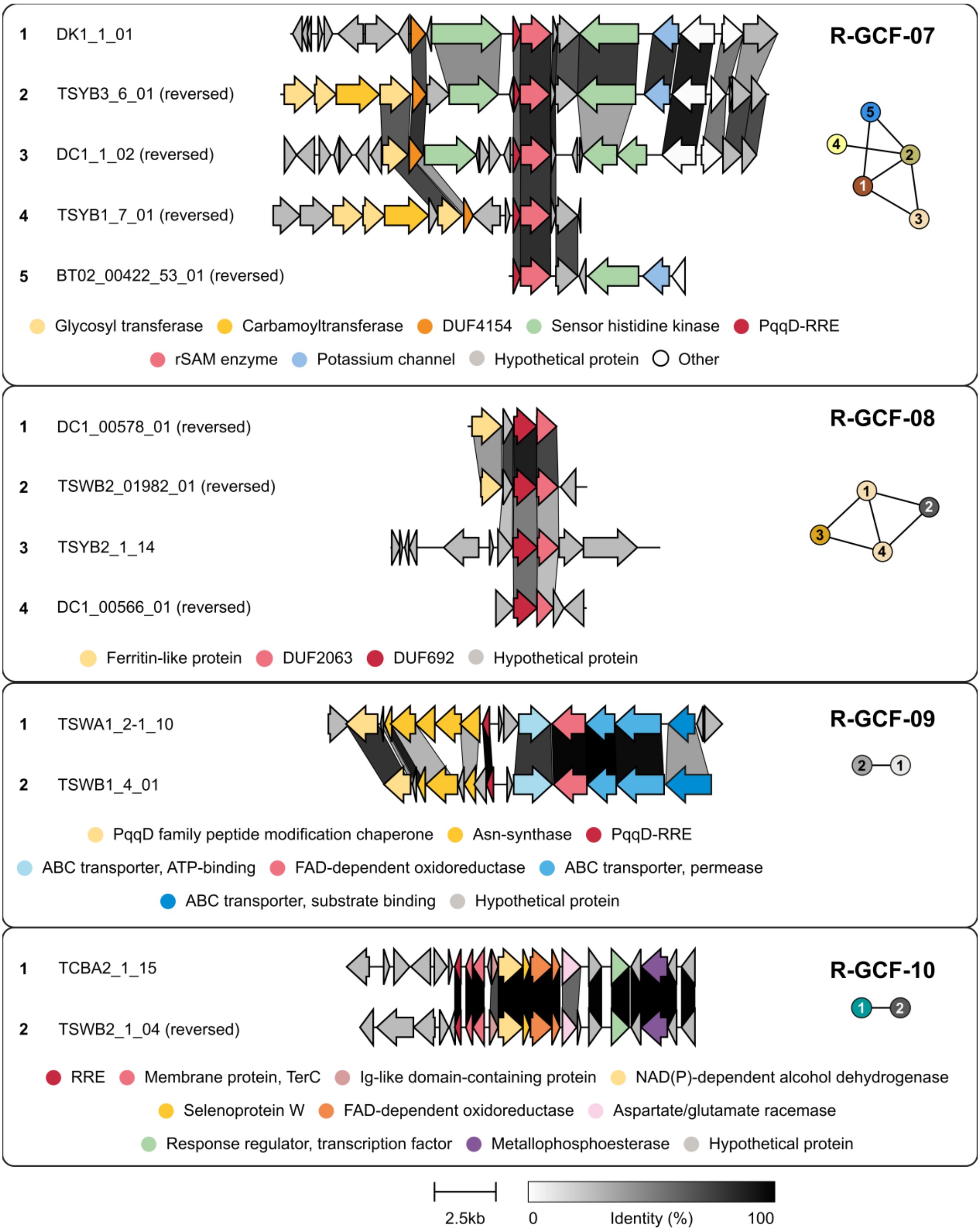

Supplementary Fig. 15: Comparison of BGCs in the RiPP biosynthetic families (R-GCF-07 to R-GCF-10). The BGCs form sub-networks (right side within the boxes) that are part of the BGC network analysis shown in Fig. 3. Shown are the BGCs in R-GCF-7 (top) R-GCF-8 (middle top), R-GCF-9 (middle bottom) and R-GCF-10 (bottom) using clinker[8] with a cutoff of 0.3. Shared genes are connected by black/grey shades.

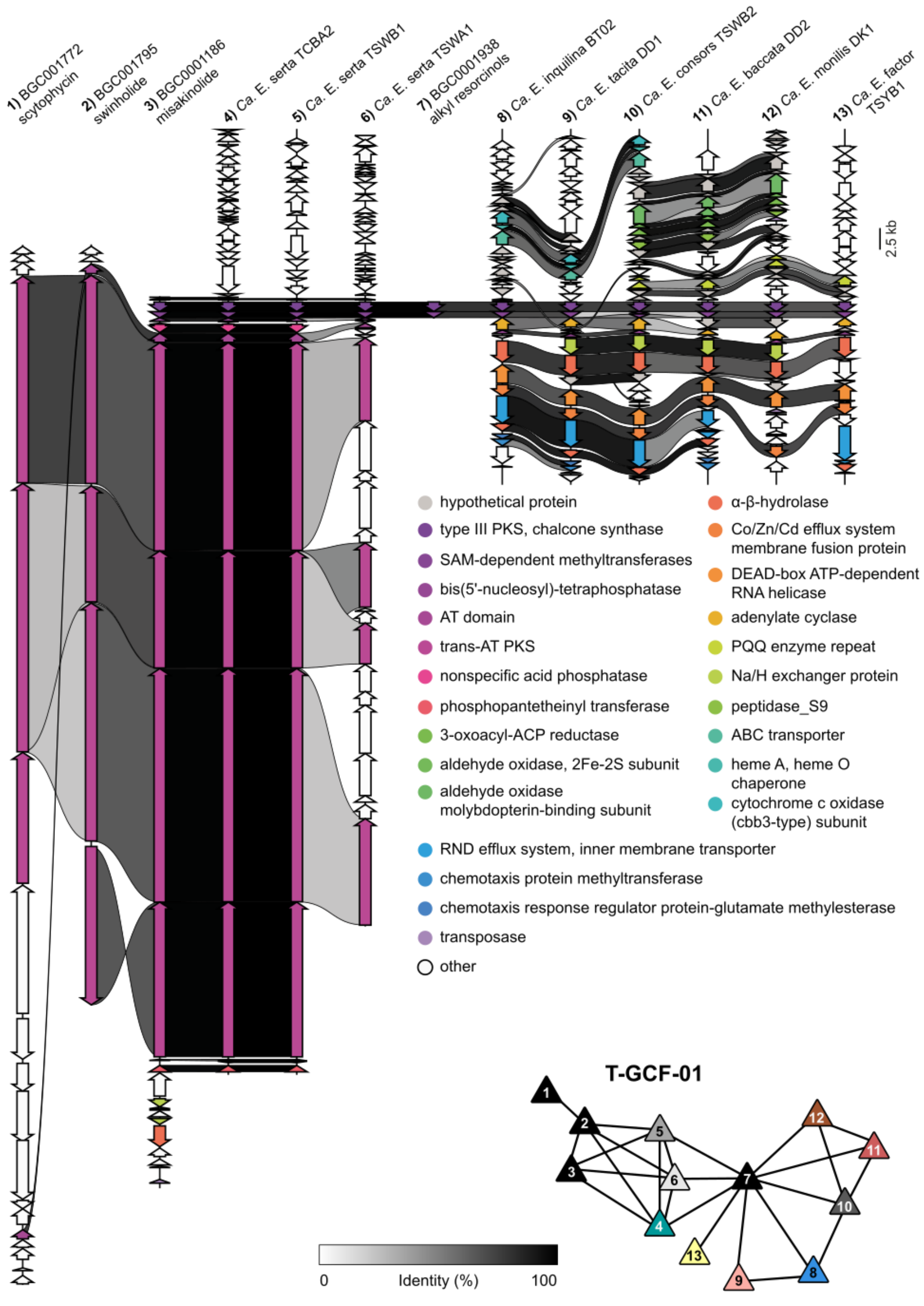

Supplementary Fig. 16: Comparison of BGCs in T-GCF-01. The BGCs form a sub-network (bottom right to the BGCs) that are part of the BGC network analysis shown in Fig. 3. Comparison of BGCs in T-GCF-01 was done using clinker[8] with a cutoff of 0.3. One central gene encoding a type III PKS that had previously been linked to the biosynthesis of resorcinols and hydroquinones[10] was identified in 9 of the total 18 genomes from this study. The large *trans*-AT PKS locus shared in 6 genomes encodes the biosynthesis of the structurally related misakinolides (BGC0001186) and swinholides. Shared genes are connected by black/grey shades.

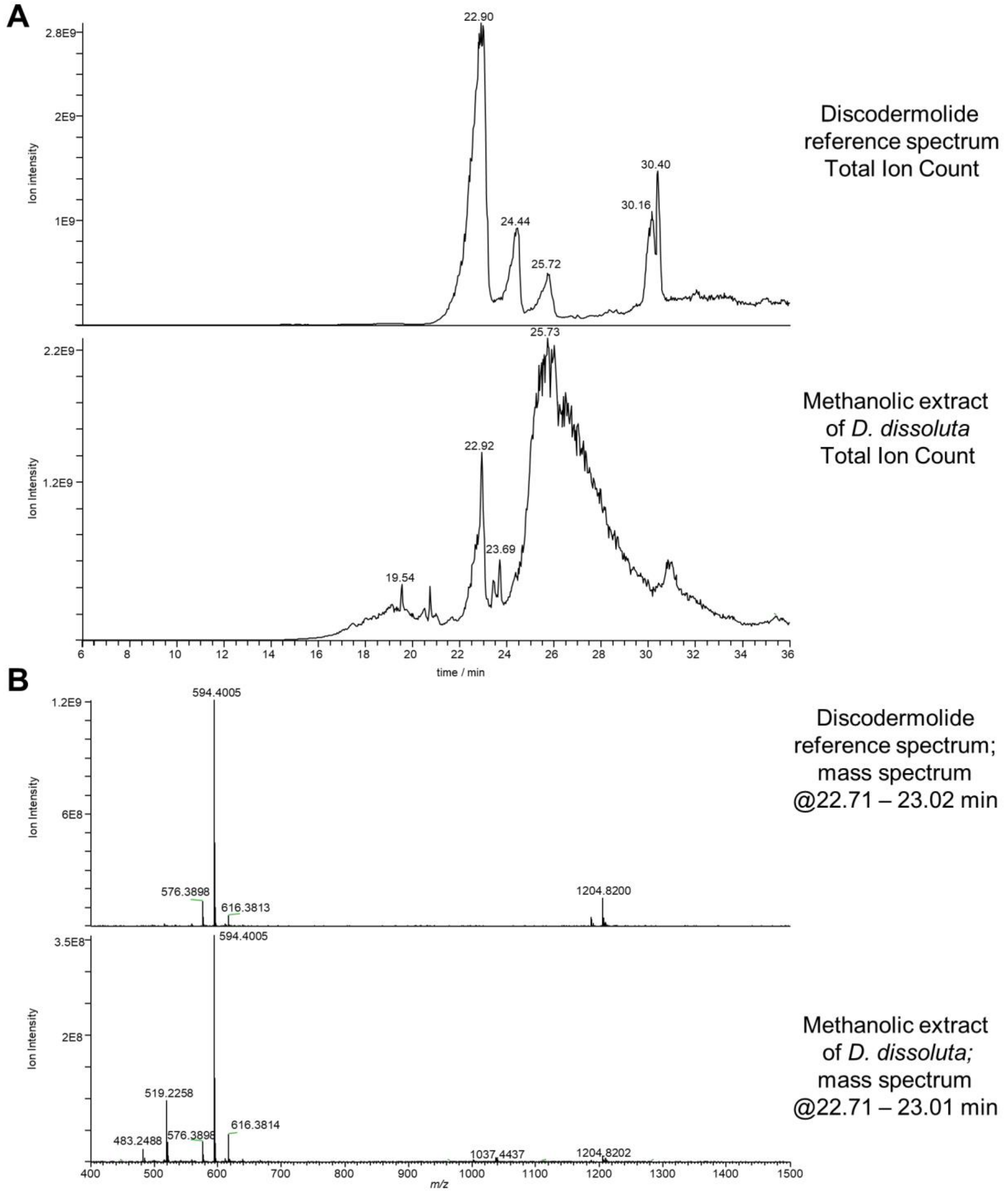

Supplementary Fig. 17: Discodermolide detection in methanolic extracts of the sponge *Discodermia dissoluta*. **A**, The discodermolide reference compound and the methanolic sponge extract were analysed using HPLC-MS. The total ion count (TIC) is shown. **B**, The mass spectra of the HPLC-MS run at the retention time between 22.71 and 23.01 minutes are shown for the discodermolide reference compound and the methanolic sponge extract. Discodermolide is expected to have an $[M+H]^+$ of 594.40.

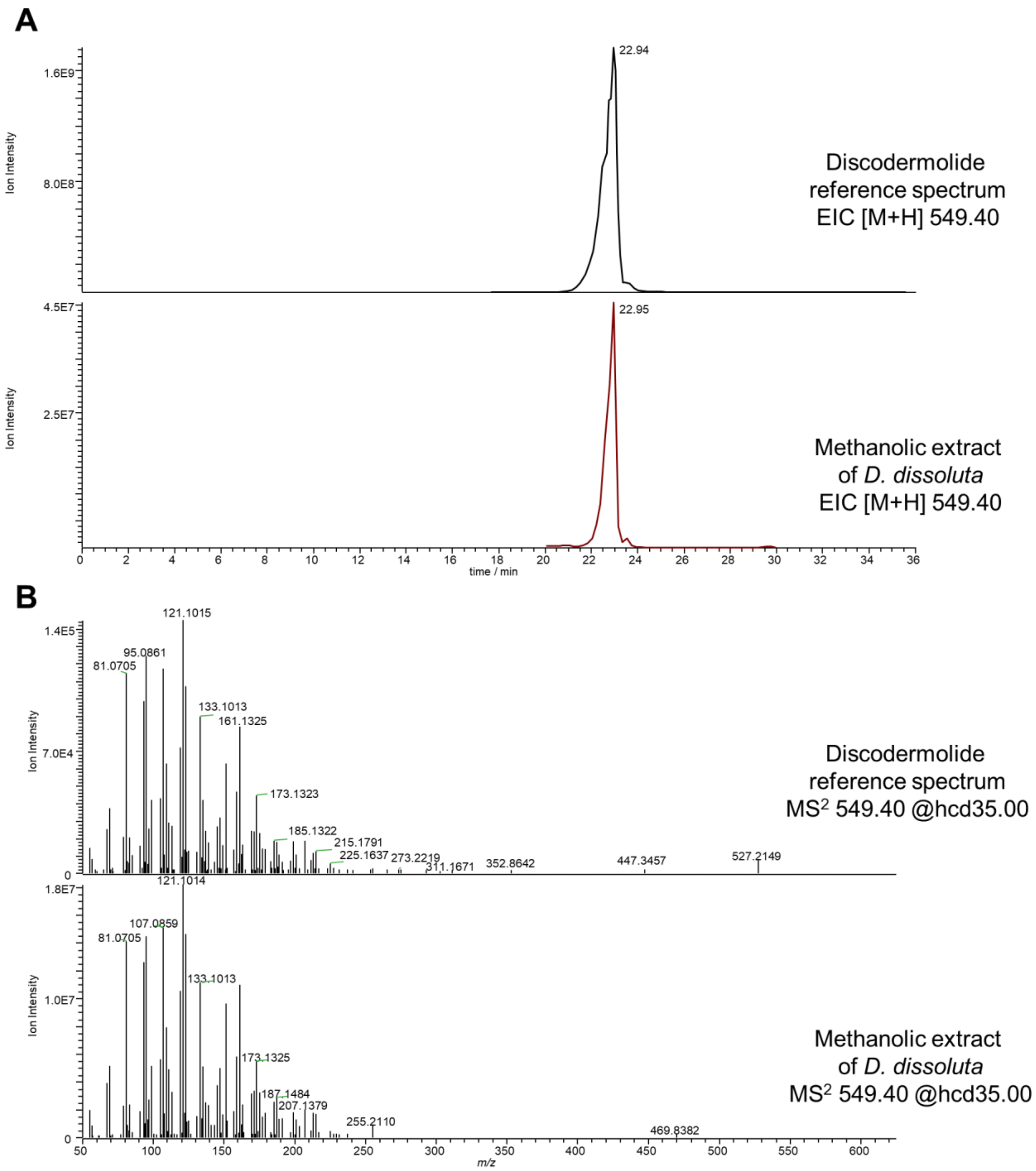

Supplementary Fig. 18: Comparison of MS^2 fragmentation patterns for discodermolide in the methanolic extracts of *Discodermia dissoluta*. A, The analysis was performed using HPLC-MS/MS. The extracted ion chromatograms (EIC) for $[M+H]^+$ 549.40 are shown for the discodermolide reference compound and the methanolic sponge extracts. The retention time for this compound in both samples is similar. **B**, Fragmentation pattern as a result of MS/MS measurements for the discodermolide reference compound and the compound identified in the methanolic sponge extract.

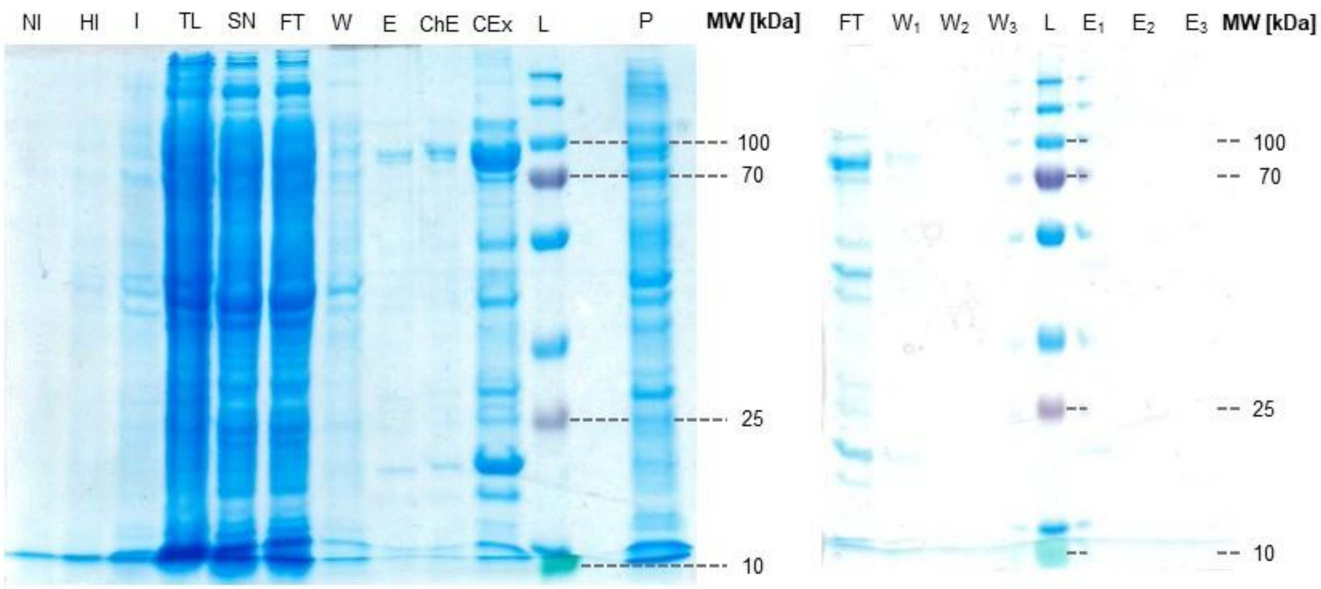

Supplementary Fig. 19: SDS-PAGE of DscE overexpression and purification. At every stage during DscE expression and purification, samples were taken for SDS-PAGE. More information can be found in the methods section. NI: not induced, HI: half-induced (more details can be found in the method section), I: induced, TL: total lysate, SN: supernatant, FT: flow-through, W: wash, E: elution, ChE: chitin bead elution, CEx: concentrated elution fraction from chitin beads, L: ladder (Thermo Scientific™ PageRuler™ Plus Prestained Protein Ladder, 10 to 250 kDa), P: pellet. For uncropped versions of these SDS-PAGE gels, please see Supplementary Fig. 34.

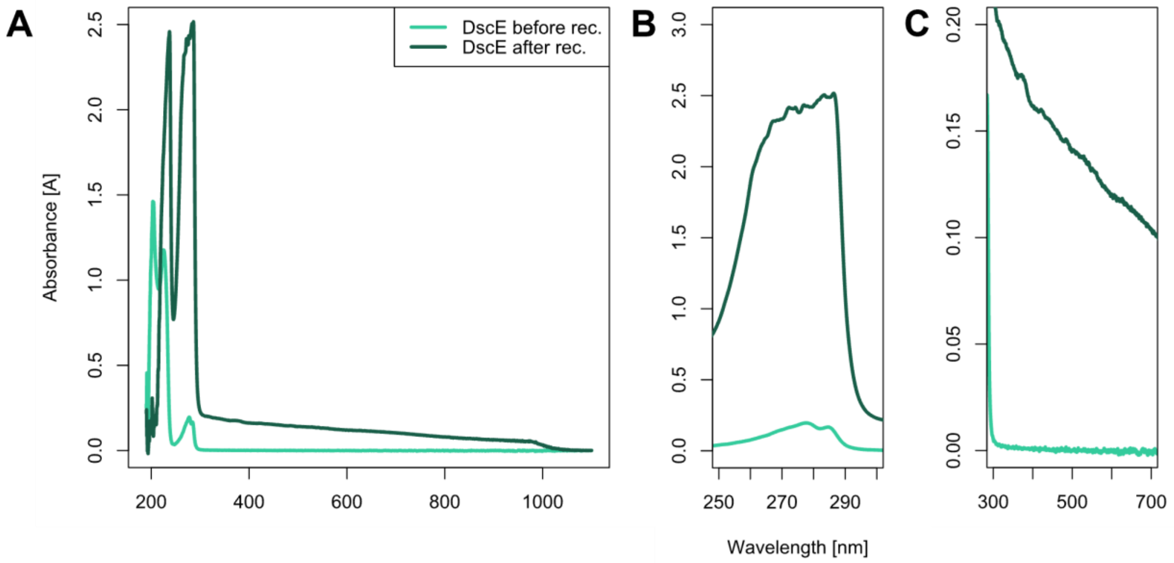

Supplementary Fig. 20: UV-Vis spectra of DscE before and after cofactor reconstitution. A, Full spectrum (200–1000 nm). **B**, Zoom in to the region showing the absorbance maximum of the protein (250 nm–300 nm). **C**, Enlargement of the region showing the absorbance maximum of the Fe-S cluster (300 nm–700 nm)

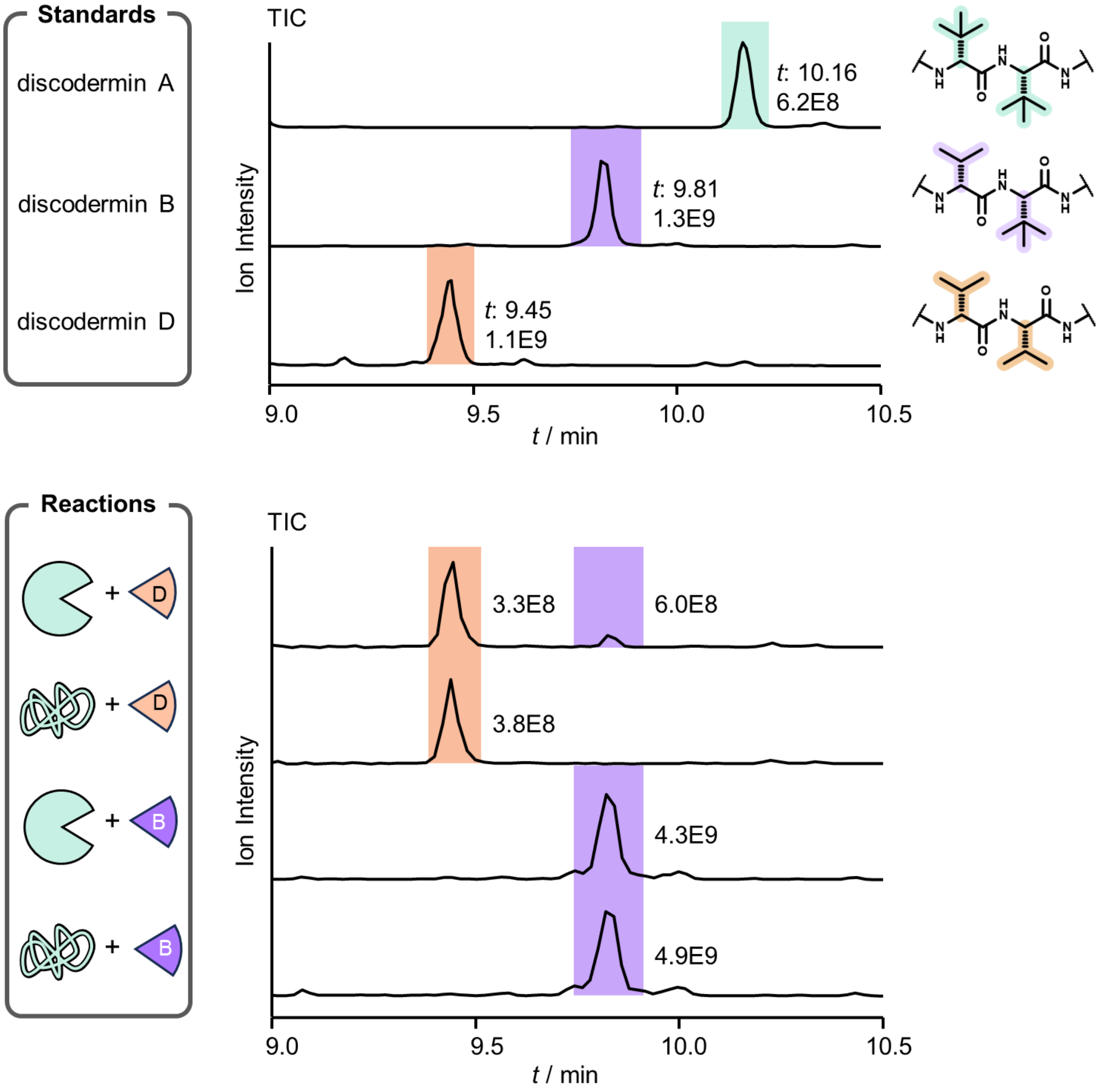

Supplementary Fig. 21: HPLC-HRMS traces (total ion count (TIC)) for the *in vitro* reconstitution of rSAM DscE methyltransferase activity. The TICs were compared to the retention times of the reference compounds discodermin A, discodermin B, and discodermin D in their TICs. In the Reactions-box, Pacman- and noodle-symbols refer to non-denatured and heat-denatured DscE, respectively, and wedges to discodermin D or B.

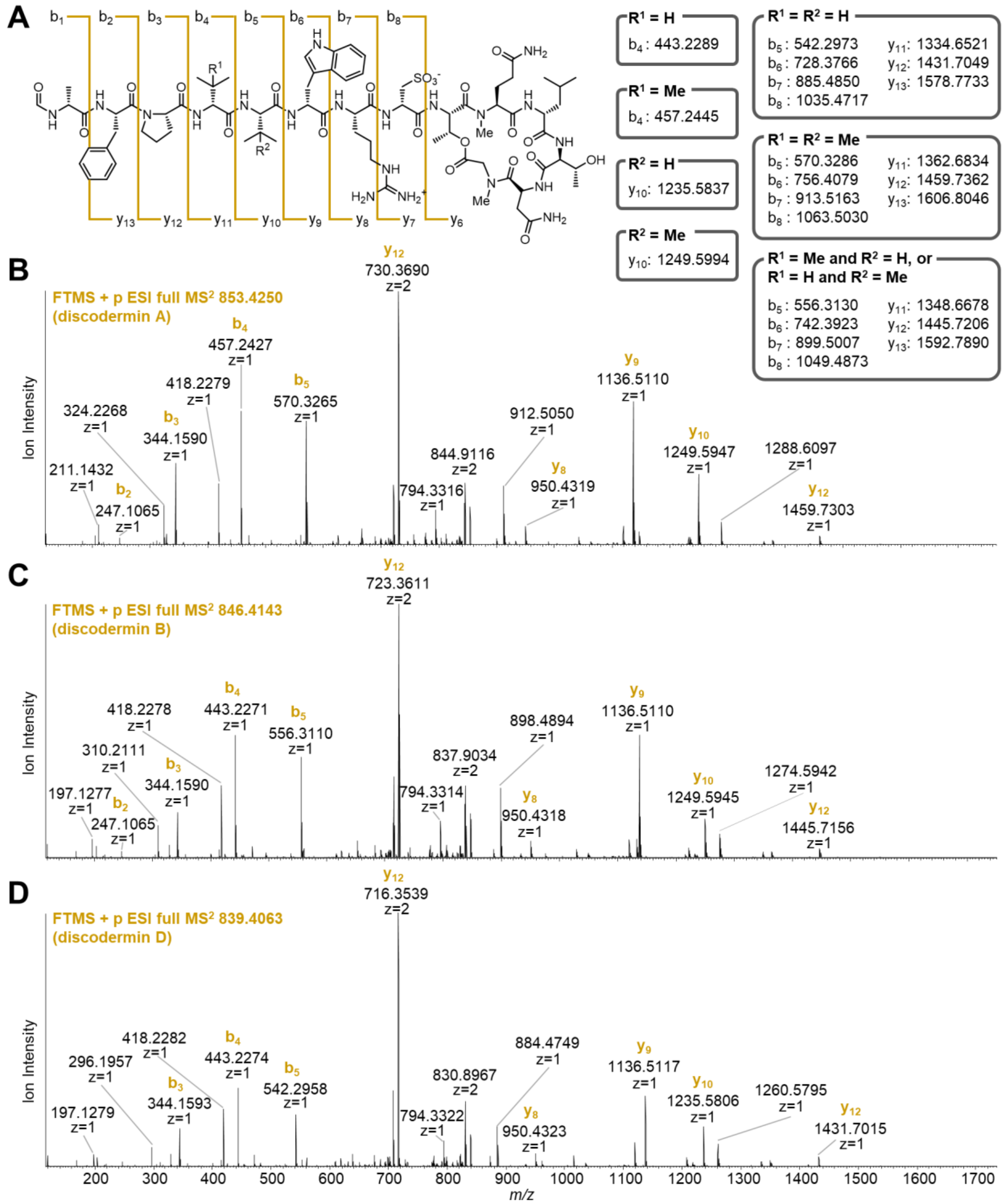

Supplementary Fig. 22: MS^2 spectra of discodermin reference compounds. A, The b- and y-ions resulting from fragmentation of the non-cyclic discodermin portion are shown. **B**, Fragmentation pattern of discodermin A. **C**, Fragmentation pattern of discodermin B. The fragment ion diagnostic for discodermin B is the b_4-ion (443.2271 m/z) that would in the case of discodermin C be detected at 457.2427 m/z. **D**, Fragmentation pattern of discodermin D.

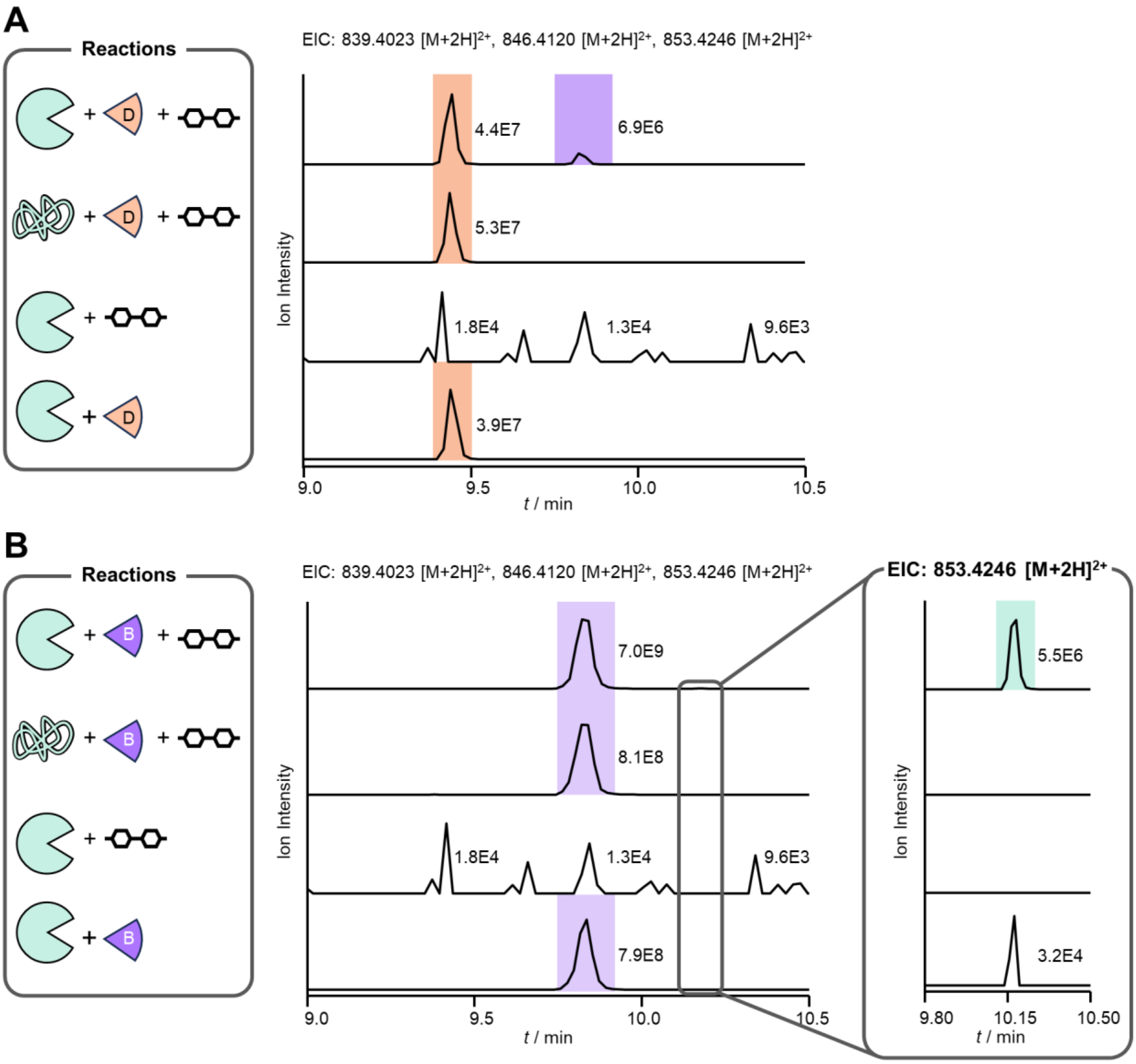

Supplementary Fig. 23: HPLC-HRMS traces for control reactions for the *in vitro* reconstitution of rSAM DscE methyltransferase activity. In control reactions, the enzyme was either heat-inactivated, or the substrate or the reduction system (methyl viologen) were omitted from the reaction. In the Reactions-boxes, Pacman- and noodle-symbols refer to non-denatured and heat-denatured DscE, respectively, wedges to discodermin D (**A**) or B (**B**), and the idealized chemical structure to methylviologen.

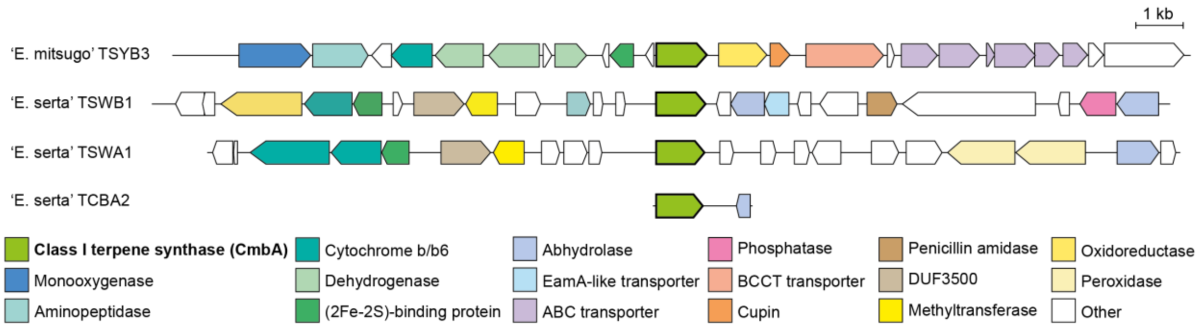

Supplementary Fig. 24: Four genome loci encoding the terpene synthase Cmb. All four *cmb* variants have different genetic environments. The terpene synthase gene was mostly likely incomplete in '*Ca.* E. serta' TCBA2, and the known amino acid sequences was identifical to that of the homolog in '*Ca.* E. serta' TSWA1. Amino acid sequences of the Cmb homolog in '*Ca.* E. serta' TSWA1 and '*Ca.* E. serta' TSWB1 differed at two positions. On the other hand, Cmb from '*Ca.* E. mitsugo' TSYB3 showed a 71% similarity to the Cmb from '*Ca.* E. serta' TSWB1. Therefore, for further characterization we chose Cmb from '*Ca.* E. serta' TSWB1 and '*Ca.* E. mitsugo' TSYB3.

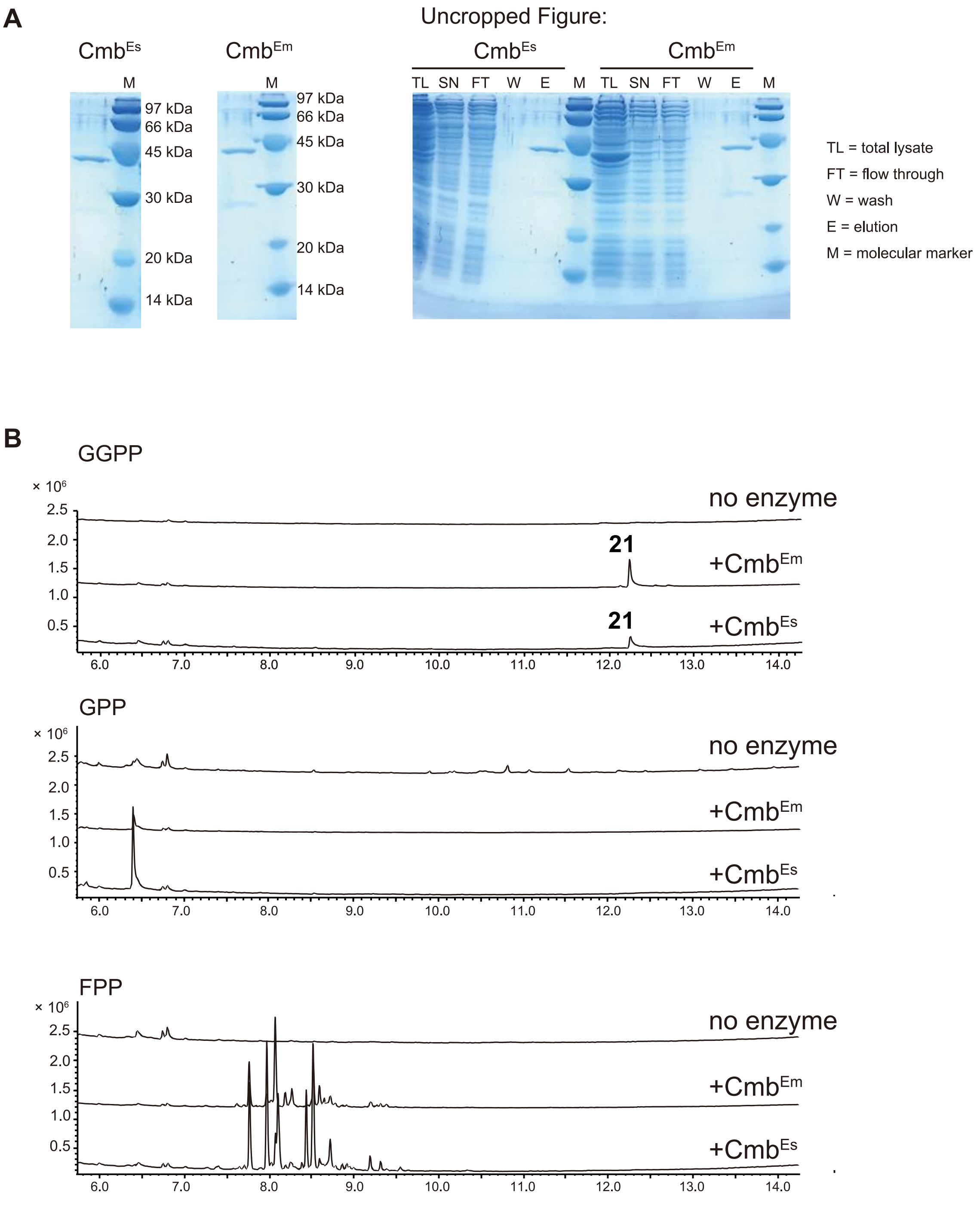

Supplementary Fig. 25: *In vitro* reconstitution of the activity of the terpene synthase Cmb. A, SDS-PAGE analysis of the Cmb variants used in this study. M, molecular weight marker. **B**, GC-MS TIC traces of ethyl acetate extracts of *in vitro* assays with GGPP, GPP, or FPP and terpene synthases.

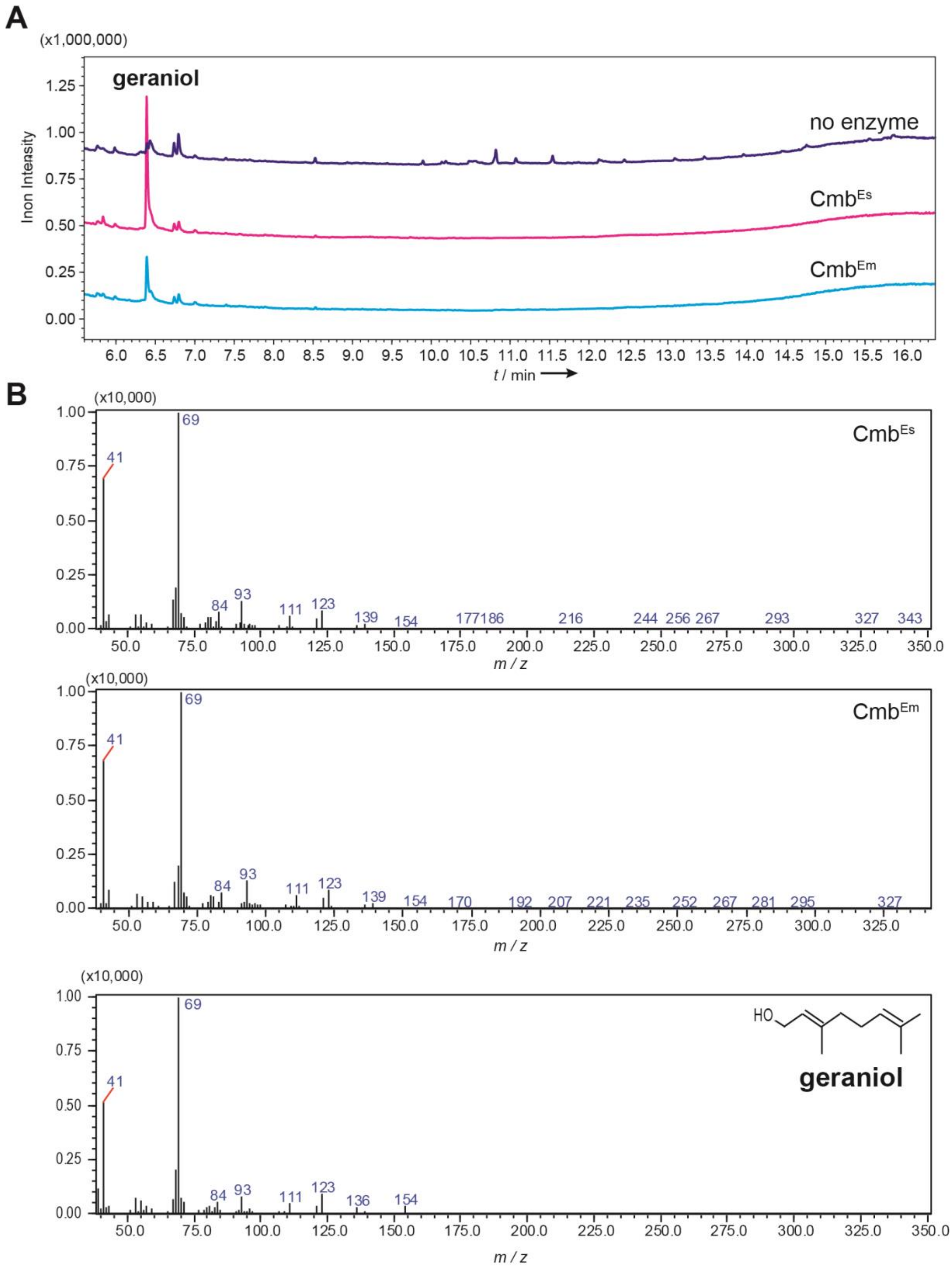

Supplementary Fig. 26: *In vitro* reconstitution of the terpene synthase Cmb with GPP as a substrate. A, GC-MS TIC traces of ethyl acetate extracts of *in vitro* assays with GPP and terpene synthases. **B**, EI mass spectra of the reaction products of either Cmb^{Es} (top) or Cmb^{Em} (middle) and geraniol (bottom)**.**

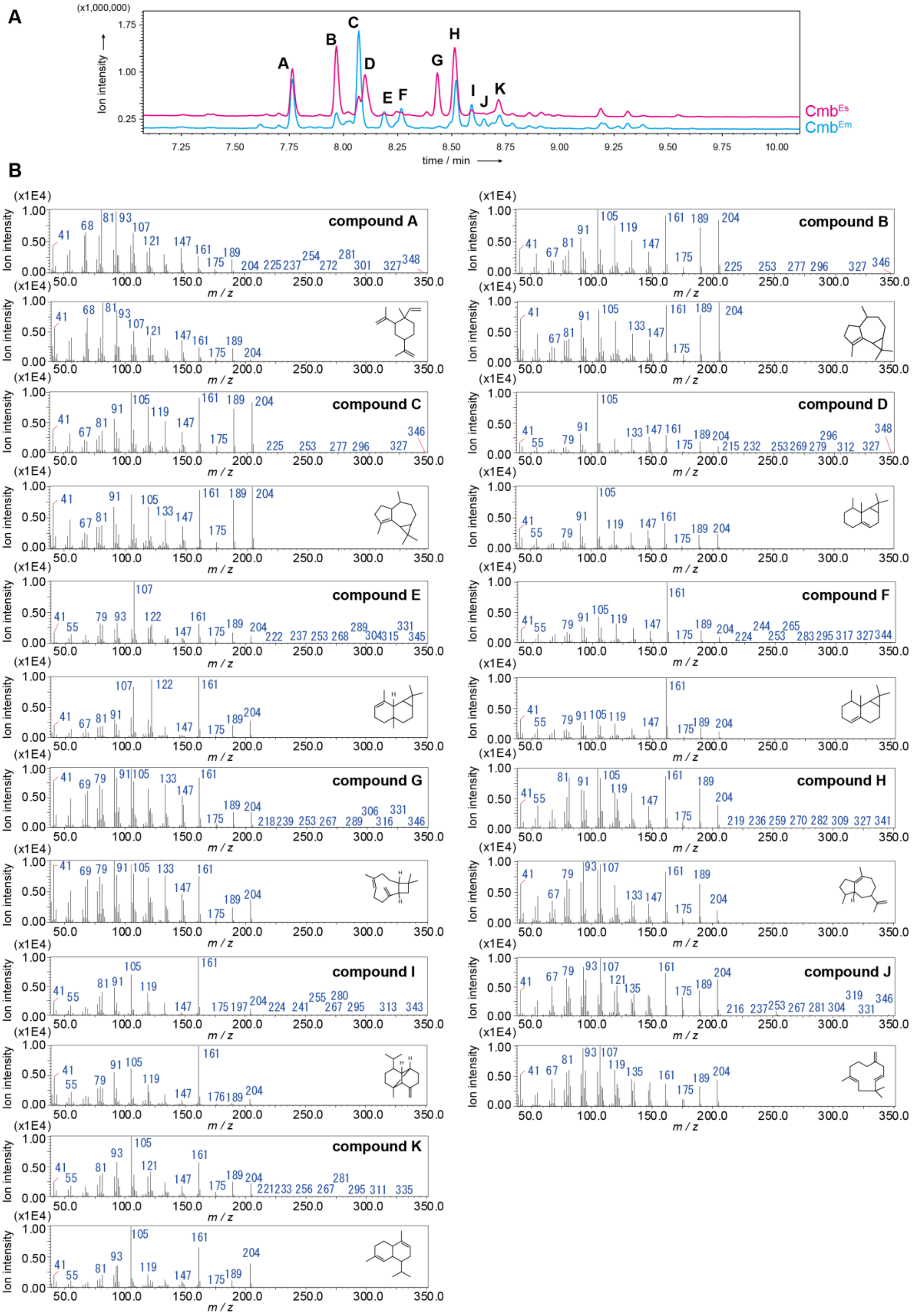

Supplementary Fig. 27: *In vitro* reconstitution of the terpene synthase Cmb with FPP as a substrate. A, GC-MS TIC traces of ethyl acetate extracts of *in vitro* assays with FPP and terpene synthases. **B**, EI mass spectra of each reaction product. Under each spectrum, the spectrum and the structures registered in the NIST MS database with the highest similarity are shown.

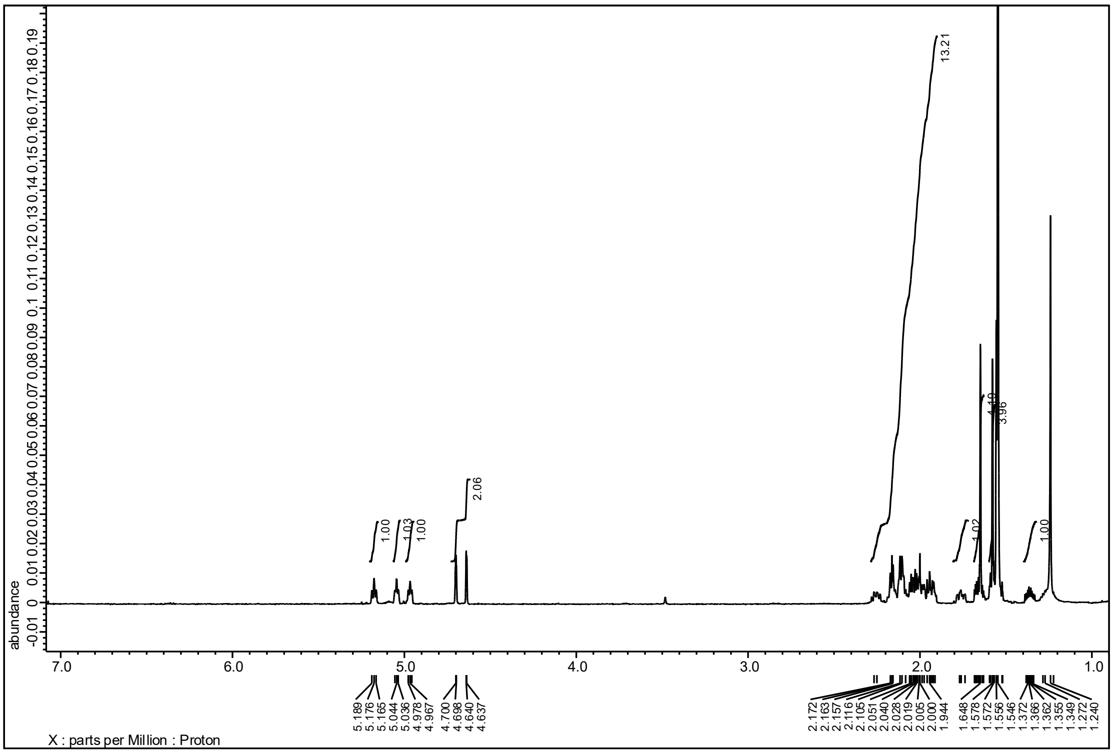

Supplementary Fig. 28: Structure elucidation of the reaction product from terpene synthase Cmb *in vitro* reconstitution. ^1H NMR spectrum (600 MHz) for cembrene A (**21**) in $CDCl_3$.

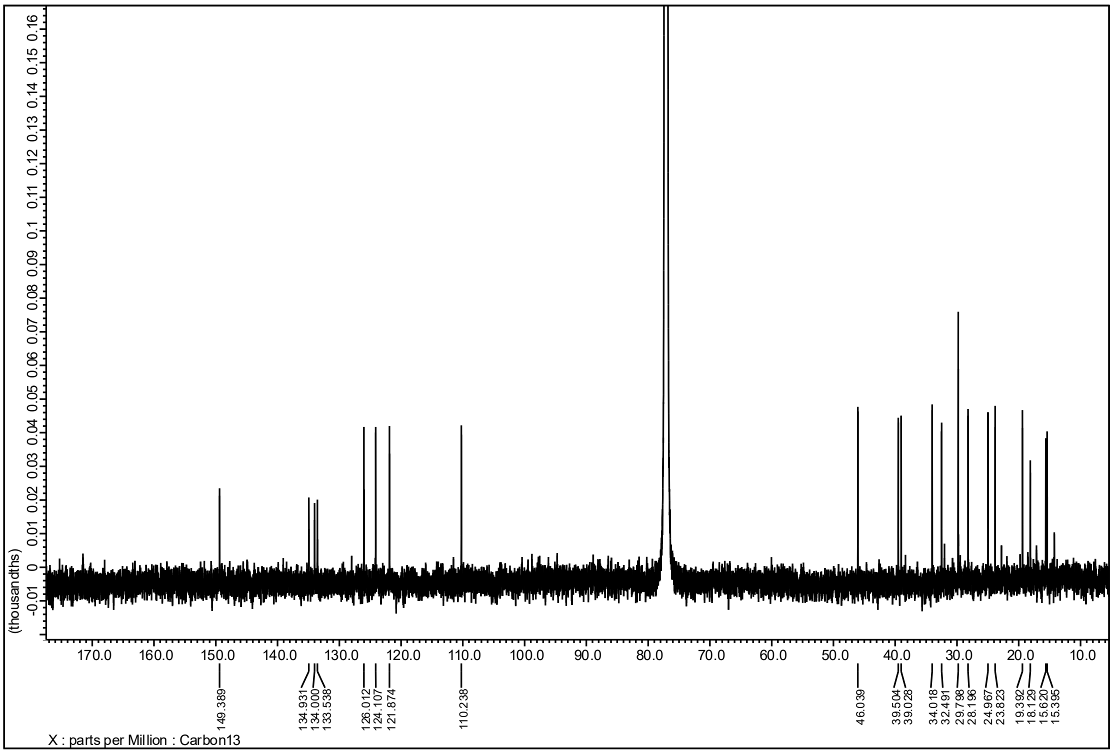

Supplementary Fig. 29: Structure elucidation of reaction product from terpene synthase Cmb *in vitro* reconstitution ^{13}C NMR spectrum (150 MHz) for cembrene A (**21**) in $CDCl_3$.

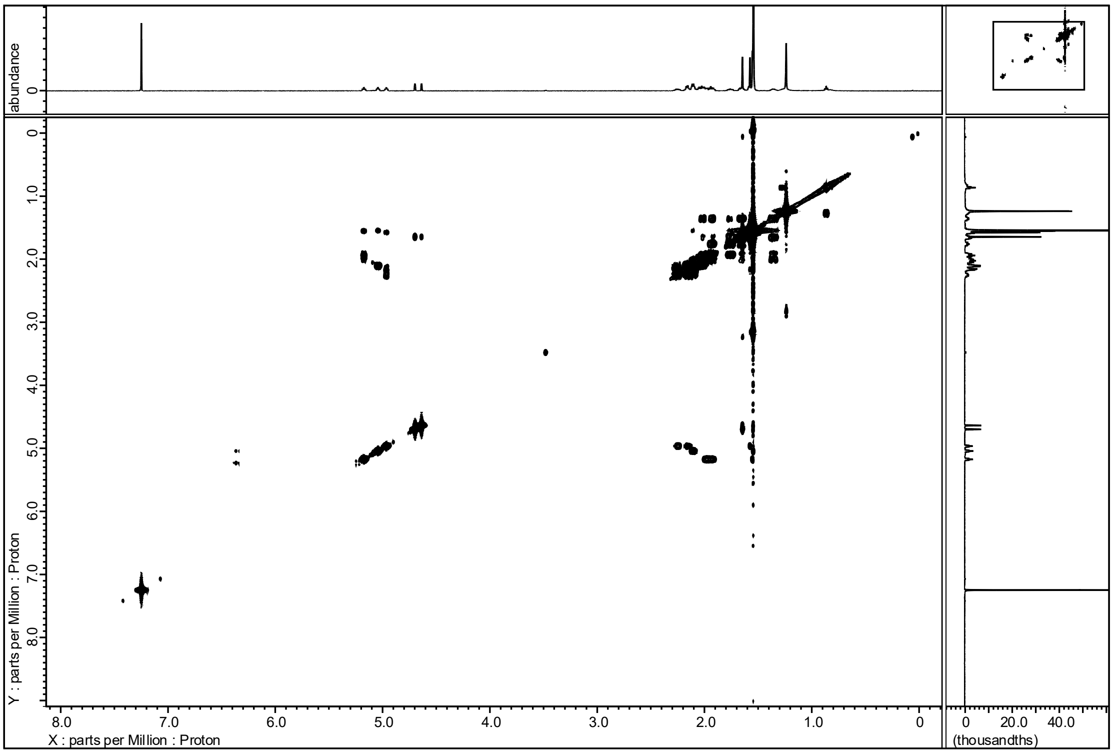

Supplementary Fig. 30: Structure elucidation of reaction product from terpene synthase Cmb *in vitro* reconstitution. COSY spectrum for cembrene A (**21**).

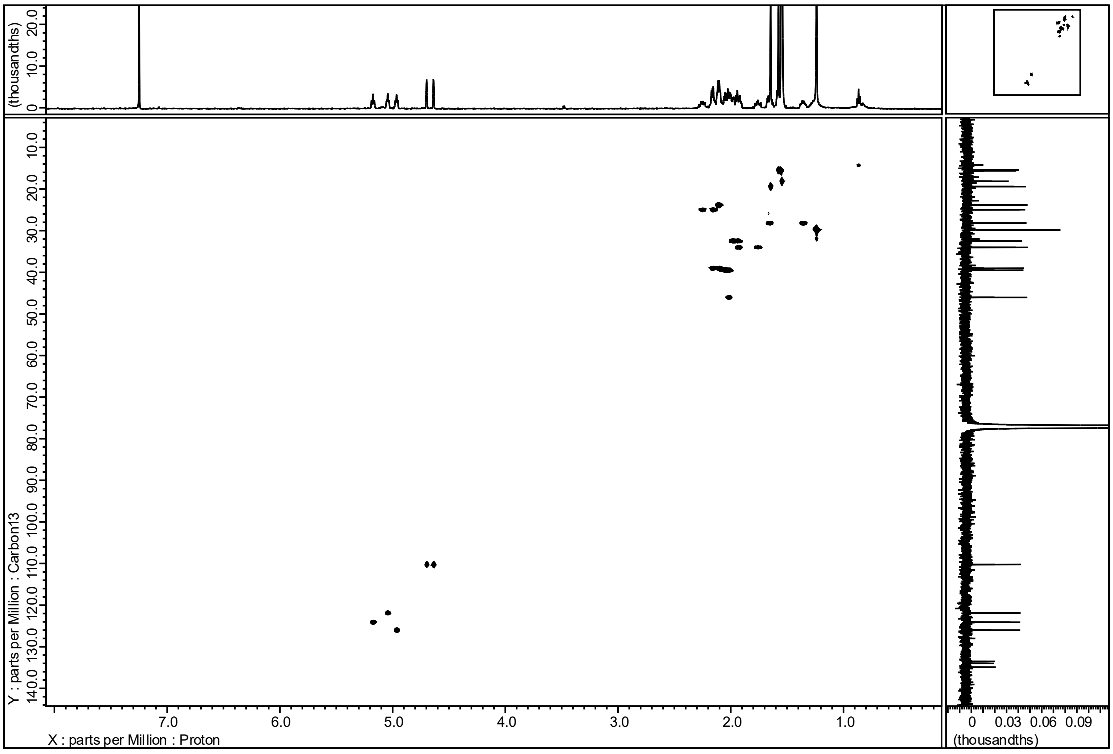

Supplementary Fig. 31: Structure elucidation of reaction product from terpene synthase Cmb *in vitro* reconstitution. HSQC spectrum for cembrene A (**21**).

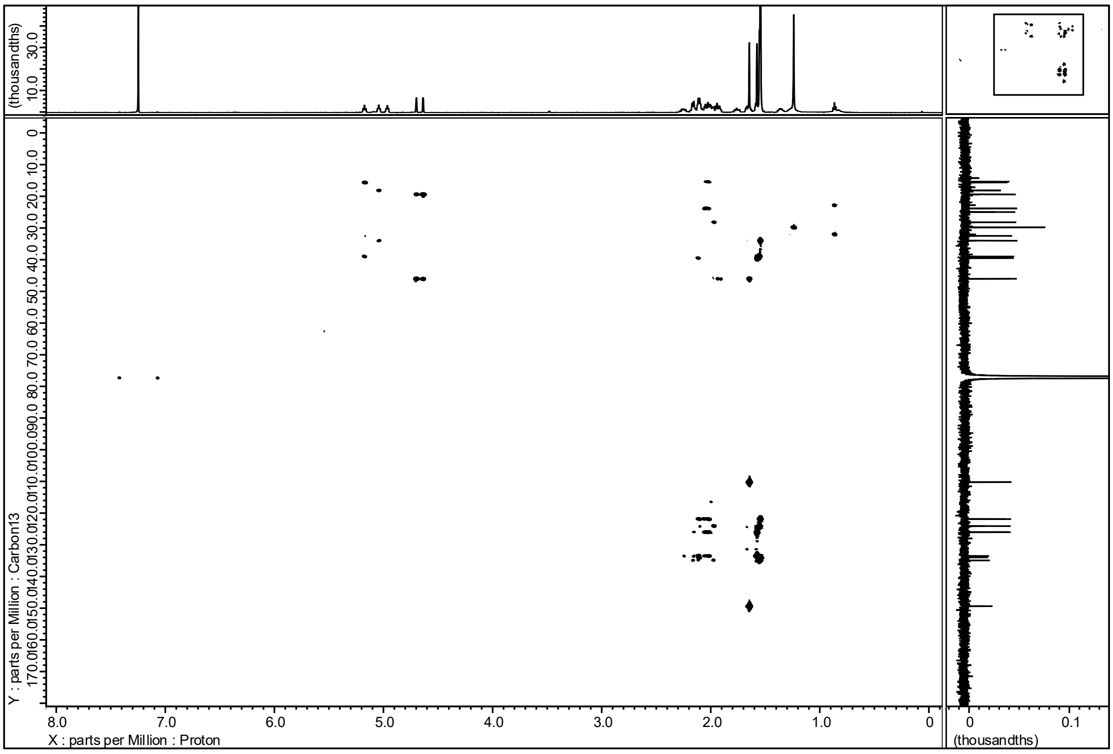

Supplementary Fig. 32: Structure elucidation of reaction product from terpene synthase Cmb *in vitro* reconstitution. HMBC spectrum for cembrene A (**21**).

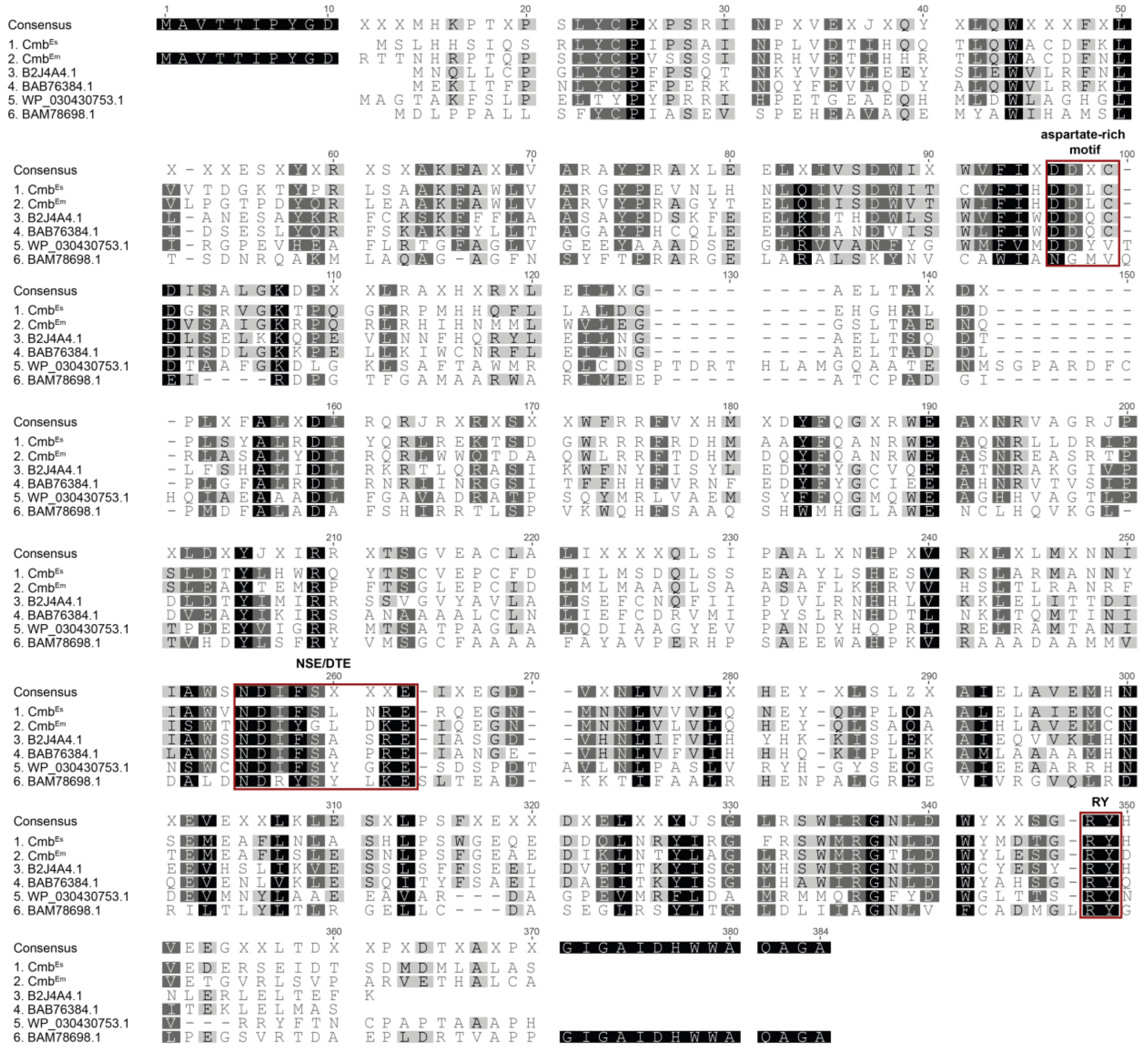

Supplementary Fig. 33: Alignment of the newly identified Cmb terpene synthases and reported microbial terpene synthases. Conserved motifs are highlighted in red boxes.

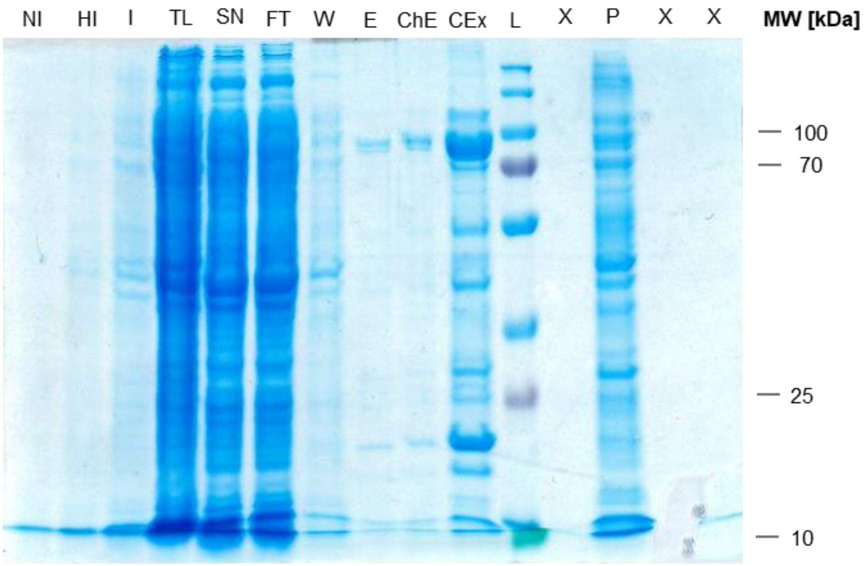

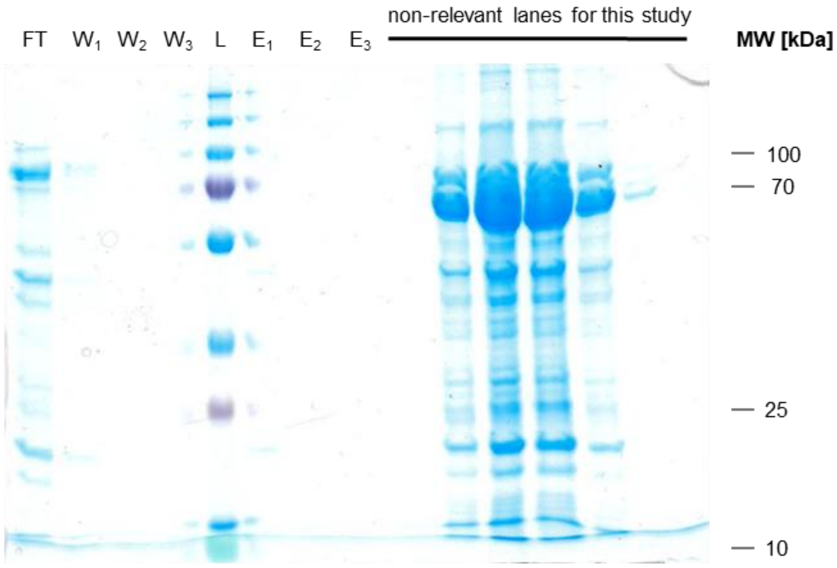

Supplementary Fig. 34: Uncropped SDS-PAGE of DscE overexpression and purification. For cropped version and more details, see Supplementary Fig. 19. NI: not induced, HI: half-induced (more details can be found in the method section), I: induced, TL: total lysate, SN: supernatant, FT: flow-through, W: wash, E: elution, ChE: chitin bead elution, CEx: concentrated elution fraction from chitin beads, L: ladder (Thermo Scientific™ PageRuler™ Plus Prestained Protein Ladder, 10 to 250 kDa), P: pellet, X: empty lane.

Supplementary Tables

Supplementary Table 1: List of sponge samples analyzed in this study.

Sponge (suborder)	Collection site (geographic coordinates, depth, year)	Known compounds (numbers in parentheses refer to representative structures in Fig. 1)	Ref.
Theonella swinhoei Y (Astrophorina)	Hachijo-Jima, Japan (33°13'46.2"N 140°13'28.2"E, 15 m, 2002 & 2011)	Konbamide (**2**), keramamides (**3**), nazumamide A (**4**), pseudotheonamides (**5**), cyclotheonamide (**6**), onnamide and theopederins (**7**), polytheonamides (**8**), aurantosides, sterols	[11]
Theonella swinhoei YB (Astrophorina)	Hachijo-Jima, Japan (33°8'16.0"N, 139°44'05.0"E, 15 m, 2017)	See above	This study
Theonella swinhoei WA (Astrophorina)	Hachijo-Jima, Japan (33°13'46.20"N 140°13'28.20"E, 15 m, 2002 & 2011)	Theonellamides (**9**), misakinolides (**10**),	[12]
Theonella swinhoei WB (Astrophorina)	Eilat, Israel, Red Sea (29°29'57.63"N, 34°54'54.61"E, 20 m, 2014)	Theonellamides (**9**), swinholides (**11**)	This study
Theonella sp. 1 BA (Astrophorina)	Mozambique Channel (16°42'47.50"S, 43°20'17.40"E, 54 m, 2015)	Unknown	This study[a]
Theonella sp. 2 BT (Astrophorina)	Shimoji-Shima, Okinawa, Japan (24°49'240."N, 125°08'07.0"E, 12 m, 2018)	Unknown	This study
Discodermia calyx (Astrophorina)	Nakagi, Shizuoka, Japan (34°61'24"N, 138°82'22"E, 10 m, 2017)	Calyculins (**12**), kasumigamide (**13**), calyxamides (**14**), indole derivatives, cyclodipeptides	This study
Discodermia kiiensis (Astrophorina)	Nakagi, Shizuoka, Japan (34°61'21"N, 138°82'20"E, 10 m, 2015)	Discodermins (**15**-**19**), lipodiscamides (**20**)	This study
Discodermia dissoluta (Astrophorina)	The Bahamas, Grand Bahama Island, Lucaya, (26°30'31.23"N, 78°35'31.17"W, 148 m, 2005)	Discodermolides	This study[b]
Aciculites cribrophora (Spirophorina)	Connor Bay, Dominica (15°38'14.13"N, 61°27'39.13"W, 213 m, 2016),	Unknown	This study[c]

[a]Sampling was performed during a cruise organized through the combined effort of the National Museum of Natural History in Paris (MNHN), the Institute for Research and Development (IRD), and the French Fisheries and Marine Institute (IFREMER). Expedition number: MOZ-04 IFREMER 2016; voucher MNHN-IP-2015-1356.

[b]Sampling was performed under a permit issued to Harbor Branch Oceanographic Institute by The Bahamas Department of Fisheries.

[c]Collected by submersible; collection number: DOM043

Supplementary Table 2: Comparison of genome assembly quality control values before and after MULTI-CSAR[13] refinement of genome assemblies for newly sequenced samples. *N50 = The size of the largest contig and contigs larger than the largest contig that have at least 50 % of the assembly bases.; **L50 = Smallest number of contigs when their length sum result in half of the genome size.

	Before MULTI-CSAR refinement					After MULTI-CSAR refinement							
Genome	N50*	L50**	# Contigs	Assembly size (bp)	%N (%)	N50	L50	# Contigs	Assembly size (bp)	%N (%)	Fold increase N50	Fold decrease L50	Fold decrease contigs
‘*Ca.* E. factor’ TSYB1	23283	541	663	9031621	5.0856	7395217	149	149	9083021	5.6228	317.6	3.6	4.4
‘*Ca.* E. gemina’ TSYB2	18203	771	929	9713960	3.4164	8044875	211	211	9785760	4.1251	442.0	3.7	4.4
‘*Ca.* E. mitsugo’ TSYB3	25906	804	935	12392349	0.0000	9091116	465	465	12439349	0.3779	350.9	1.7	2.0
‘*Ca.* E. serta’ TSWA1	7258	2001	2339	8698461	0.0148	5792140	635	635	8984442	2.1525	798.0	3.2	3.7
‘*Ca.* E. serta’ TSWB1	12312	889	1113	9180238	0.0000	4900409	264	264	9265138	0.9163	398.0	3.4	4.2
‘*Ca.* E. consors’ TSWB2	12056	1005	1259	9528011	0.0000	8009554	355	355	9618411	0.9399	664.4	2.8	3.5
‘*Ca.* E. melakyensis’ TCBA1	8242	821	1115	7739606	0.00	6421244	169	169	7834206	1.2075	779.1	4.9	6.6
‘*Ca.* E. serta’ TCBA2	4123	1977	2643	9001400	0.0000	6985004	273	273	9238400	2.5654	1694.2	7.2	9.7
‘*Ca.* E. symbiotica’ BT01	9179	1978	2262	9647102	0.0000	1926660	1013	1014	9911242	1.5962	209.9	2.0	2.2
‘*Ca.* E. inquilina’ BT02	4763	2379	2766	6713026	0.0000	4861922	1292	1292	7225974	3.2729	1020.8	1.8	2.1
‘*Ca.* E. melakyensis’ BT03	3295	2367	2894	6119652	0.0000	4297189	787	787	6926778	5.2088	1304.2	3.0	3.7
‘*Ca.* E. catenata’ BT04	4276	2112	2493	6037420	0.0000	3995698	1171	1171	6468468	3.1507	934.4	1.8	2.1
‘*Ca.* E. monilis’ DK1	72112	225	275	12621761	0.0002	3786908	76	77	12641561	0.1568	52.5	3.0	3.6
‘*Ca.* E. armillaria’ DC1	4392	1641	2054	6912118	0.0000	4696293	561	561	7061418	2.1143	1069.3	2.9	3.7
‘*Ca.* E. tacita’ DD1	9100	546	746	5570689	0.0042	3983834	176	176	5627689	1.0170	437.8	3.1	4.2
‘*Ca.* E. baccata’ DD2	9965	684	896	6989347	0.0076	4612761	174	174	7061547	1.0300	462.9	3.9	5.1
‘*Ca.* E. tertia’ DD3	2887	590	835	2085060	0.0069	10677	408	416	2126960	1.9767	3.7	1.4	2.0
‘*Ca.* P. opulenta’ AC1	16066	513	662	7316387	0.0151	17875	495	586	7323987	0.1189	1.1	1.0	1.1

Supplementary Table 3: Statistics on the newly obtained 'Entotheonella' genome sequences as calculated using CheckM[14]. SAG, single-bacterial amplified genome; LR, Long Read; MAG, metagenome-assembled genome.

Sponge source	Genome	Sequencing method	Assembly size (Mbp)	Completeness (%)	Contamination (%)	Estimated genome size (Mbp)
Theonella swinhoei YB (Japan)	'*Ca.* E. factor' TSYB1	SAG & LR	9.08	91.50	13.68	8.57
	'*Ca.* E. gemina' TSYB2	SAG & LR	9.79	92.18	9.57	9.56
	'*Ca.* E. mitsugo' TSYB3	SAG & LR	12.44	85.30	4.27	13.96
Theonella swinhoei WA (Japan)	'*Ca.* E. serta' TSWA1	SAG	8.80	75.64	10.68	10.39
Theonella swinhoei WB (Israel)	'*Ca.* E. serta' TSWB1	MAG	9.27	93.96	7.79	9.09
	'*Ca.* E. consors' TSWB2	MAG	9.62	95.67	7.86	9.26
Theonella sp. 1 BA (Mozambique Channel)	'*Ca.* E. melakyensis' TCBA1	MAG	7.83	91.87	14.03	7.33
	'*Ca.* E. serta' TCBA2	MAG	9.24	79.09	5.60	11.03
Theonella sp. 2 BT (Japan)	'*Ca.* E. symbiotica' BT01	SAG	9.60	89.26	8.02	9.89
	'*Ca.* E. inquilina' BT02	SAG	6.75	63.64	5.98	9.97
	'*Ca.* E. melakyensis' BT03	SAG	6.66	72.68	4.27	8.77
	'*Ca.* E. catenata' BT04	SAG	6.03	52.99	0.85	11.29
Discodermia kiiensis (Japan)	'*Ca.* E. monilis' DK1	SAG & LR	12.64	91.83	6.41	12.88
Discodermia calyx (Japan)	'*Ca.* E. armillaria' DC1	SAG & LR	7.06	72.74	1.71	9.54
Discodermia dissoluta (The Bahamas)	'*Ca.* E. tacita' DD1	MAG	5.63	92.25	12.11	5.34
	'*Ca.* E. baccata' DD2	MAG	7.06	90.54	7.69	7.20
	'*Ca.* E. tertia' DD3	MAG	2.13	12.86	0.00	16.54
Aciculites cribrophora (Dominica)	'*Ca.* P. opulenta' AC1	MAG	7.28	94.82	5.30	7.27

Supplementary Table 4: The number of biosynthetic gene clusters (BGCs) or BGC fragments is based on an analysis with antiSMASH[15]. Assignments of compounds to BGCs are based on either biosynthetic logic or functional studies.

'Entotheonella'	BGCs (fragments)	Assigned compounds
'*Ca.* E. factor' TSYB1	29	
'*Ca.* E. gemina' TSYB2	27	Polytheonamides[16], konbamides, keramamides, nazumamides, onnamides, cyclotheonamides,
'*Ca.* E. mitsugo' TSYB3	35	
'*Ca.* E. serta' TSWA1	35	Theonellamides, misakinolides
'*Ca.* E. serta' TSWB1	35	Theonellamides, swinholides
'*Ca.* E. consors' TSWB2	29	
'*Ca.* E. melakyensis' TCBA1	18	
'*Ca.* E. serta' TCBA2	38	Theonellamides, misakinolides/swinholides
'*Ca.* E. symbiotica' BT01	41	
'*Ca.* E. inquilina' BT02	26	
'*Ca.* E. melkyensis' BT03	10	
'*Ca.* E. catenata' BT04	25	
'*Ca.* E. armillaria' DC1	31	Calyculins, kasumigamides
'*Ca.* E. monilis' DK1	27	Discodermins, lipodiscamides
'*Ca.* E. tacita' DD1	18	
'*Ca.* E. abita' DD2	27	
'*Ca.* E. tertia' DD3	10	
'*Ca.* P. opulenta' AC1	18	

Supplementary Table 5: Biosynthetic potential, genome quality metrics, sample of origin, and taxonomy for the 63 Tectomicrobia MAGs identified in the mOTUs database v4.0 [17]. The biosynthetic potential is captured by the Biosynthetic Gene Clusters (BGCs) predicted by antiSMASH (v6.1) and further curated manually (BGC contigs, BGC contigs curated). Manual curation consisted of eliminating BGCs with an architecture suggesting a function other than secondary metabolism (carotenoids, hopanoids, ladderane, PKS-like type I fatty acid synthase), as well as genes encoding single NRPS modules. Genome quality metrics (Q Score (Completeness – 5 x Contamination), Completeness, Contamination), the bioinformatic tool used to compute those metrics (Tool), the biosample of origin (NCBI sample), and the species-level taxonomy (Taxon) are derived from the mOTUs database v.4.0 metadata. The table is provided as a separate Excel file.

Supplementary Table 6: The *cax* BGC encodes biosynthetic enzymes putatively catalyzing calyxamide biosynthesis. The table shows the encoded proteins, their respective size, the proposed function and the closest homolog together with its sequence identify and accession number. NRPS, nonribosomal peptide synthetase; Fmt, formyltransferase; C, condensation domain; CP, carrier protein; A, adenylation domain; PKS, polyketide synthase; KS, ketosynthase; AT, acyltransferase; KR, ketoreductase; MT, methyltransferase; Ox, oxygenase; DH, dehydratase domain; TE, thioesterase.

Protein	Protein size (aa)	Proposed function	Closest homolog, protein (origin)	Identity	Accession number
CaxA	2263	NRPS (domains: Fmt-C-CP-C-A-CP)	amino acid adenylation domain-containing protein (*Dolichospermum* sp. UHCC 0352)	49.60%	MTJ20109.1
CaxB	1067	NRPS (A-CP)	amino acid adenylation domain-containing protein (*Pseudanabaenales* cyanobacterium)	50.65%	MCG8363543.1
CaxC	2251	NRPS (domains: C-A-CP-C-A-CP)	hybrid non-ribosomal peptide synthetase/type I polyketide synthase (*Scytonema hofmannii*)	58.81%	WP_017748552.1
CaxD	1577	PKS (domains: KS-AT-KR-CP)	KrmO (‘*Ca.* Entotheonella serta’ TSWA1)	78.67%	UZC79443.1
CaxE	411	Luciferase	KrmP (‘*Ca.* Entotheonella serta’ TSWA1)	82.77%	UZC79444.1
CaxF	1427	NRPS (domains: C-A-MT-CP)	KrmR (‘*Ca.* Entotheonella serta’ TSWA1)	83.56%	UZC79447.1
CaxG	1413	NRPS (domains: HC-A-OX-CP)	KrmS (‘*Ca.* Entotheonella serta’ TSWA1)	86.89%	UZC79448.1
CaxH	1816	PKS (domains: KS-AT-DH-KR-CP)	hypothetical protein ETSY1_00130 (‘*Ca.* Entotheonella factor’)	60.88%	ETX03402.1
CaxI	1401	NRPS- (domains: C-A-CP-CP-TE)	hypothetical protein ETSY1_00135 (‘*Ca.* Entotheonella factor’)	78.34%	ETX03403.1

Supplementary Table 7: Predicted selectivity-conferring codes for A domains in discodermin, lipodiscamide and calyxamide biosynthesis and the respective prediction of substrate specificitiy based on antiSMASH. Kiv, α-ketoisovaleric acid; Dpr, 2,3-diaminopropionic acid.

Adenylation domain	Active site code	Substrate specificity prediction (nonribosomal code match)
DscA_A1	DLFNNALTYK	Ala (100%)
DscA_A2	DAWVIAAVCK	Phe (90%)
DscA_A3	DVQFIAHVVK	Pro (100%)
DscB_A4	DAFWLGGTFK	Val (100%)
DscB_A5	DAFWLGGTFK	Val (100%)
DscB_A6	DAALIGEVWK	Trp (90%)
DscC_A7	DPEDIGAITK	Arg (90%)
DscC_A8	DSTKIGHVGK	Asp (90%)
DscC_A9	DFWNIGMVHK	Thr (100%)
DscC_A10	DAWQFGLIDK	Gln (100%)
DscD_A11	DAWFLGQVVK	Leu (100%)
DscD_A12	DFWNIGMVHK	Thr (100%)
DscD_A13	DATKVGEVGK	Asn (100%)
DscD_A14	DILQLGMIWK	Gly (100%)
LpcE_A1	GLFWIAASAK	Kiv (80%)
LpcG_A2	DFWNIGMVHK	Thr (100%)
LpcG_A3	DVENIGAITK	Arg (90%)
LpcH_A4	DAWHISLVDK	Ser (90%)
LpcH_A5	DAQDQAIVNK	Dpr (80%)
LpcH_A6	DILQWGLIWK	Gly (100%)
CaxA_A1	DAWQFGLIDK	Gln (100%)
CaxA_A2	DAFFLGVTYK	Ile (100%)
CaxB_A3	DAFTAALIWK	Phe-Ser or Phe (80%)
CaxC_A4	DLFNNALTYK	Ala (100%)
CaxC_A5	DAFFLGVTYK	Ile (100%)
CaxF_A6	DVWHISLIDK	Ser (100%)
CaxG_A7	DLYDMSLIWK	Cys (90%)
CaxI_A8	DALHVGNVAK	Leu (100%)

Supplementary Table 8: The *lpc* BGC encodes biosynthetic enzymes putatively catalyzing lipodiscamide biosynthesis. The table shows the encoded proteins, their respective size, the proposed function and the closest homolog together with its sequence identify and accession number. PKS, polyketide synthase; KS, ketosynthase; AT, acyltransferase; CP, carrier protein; DH, dehydratase domain; OMT, *O*-methyltransferase; NRPS, nonribosomal peptide synthetase; KR, ketoreductase; C, condensation domain; A, adenylation domain; E, epimerization domain; CAL, coenzyme A ligase domain; CMT, *C*-methyltransferase; TE, thioesterase.

Protein	Protein size (aa)	Proposed function	Closest homolog, protein (origin)	Identity	Accession number
LpcA	1612	PKS (domains: KS-AT-KR-CP)	SDR family NAD(P)-dependent oxidoreductase (*Archangium minus*)	48.84%	WNG46910.1
LpcB	98	KS domain	polyketide synthase (*Desmonostoc muscorum* FACHB-395)	67.16%	MBD2512887.1
LpcC	1010	PKS (domains: KS-DH-CP)	type I polyketide synthase (*Nostoc* sp. UHCC 0702)	54.76%	QSJ15494.1
LpcD	1749	PKS (domains: KS-OMT-KR-CP)	type I polyketide synthase (*Chondromyces crocatus*)	53.60%	WP_050432595.1
LpcE	3623	PKS-NRPS hybrid (domains: KS-DH-CMT-KR-CP-C-A-KR-CP)	SDR family NAD(P)-dependent oxidoreductase (*Chroococcidiopsidaceae* cyanobacterium CP_BM_ER_R8_30)	44.62%	MBV9390134.1
LpcF	1853	PKS (domains: KS-AT-KR-DH-CP)	SDR family NAD(P)-dependent oxidoreductase (*Nostoc* sp.)	47.26%	WP_335202642.1
LpcG	2611	NRPS (domains: C-A-CP-C-A-CP-E)	non-ribosomal peptide synthase / polyketide synthase (*Nostoc* sp. TH1S01)	45.03%	MBU7585508.1
LpcH	3388	NRPS (domains: C-A-CP-C-A-CP-C-A-CP-TE	non-ribosomal peptide synthetase (*Archangium primigenium*)	46.25%	WP_204488109.1
LpcI	1293	NRPS-like (domains: CAL-CP)	AMP-binding protein (*Anaerolineae* bacterium)	54.22%	MCB0167207.1

Supplementary Table 9: The *dsc* BGC encoding discodermin biosynthesis. The table shows the encoded proteins, their respective size, the proposed function and the closest homolog together with its sequence identify and accession number. NRPS, nonribosomal peptide synthetase; Fmt, formyltransferase; C, condensation domain; A, adenylation domain; CP, carrier protein; E, epimerization domain; nMT, *N*-methyltransferase; MbtH, helper protein for A domain; TE, thioesterase.

Protein	Protein size (aa)	Proposed function	Closest homolog, protein (origin)	Identity	Accession number
DscA	3759	NRPS (domains: Fmt-A-E-CP-C-A-CP-C-A-CP)	non-ribosomal peptide synthase/polyketide synthase (*Scytonema* sp. UIC 10036)	52.05%	WP_166481987.1
DscB	4183	NRPS (domains: C-A-E-CP- C-A-CP-C-A-E-CP)	non-ribosomal peptide synthase (*Methylocaldum marinum*)	51.80%	BBA33066.1
DscC	5146	NRPS (domains: C-A-CP-C-A-E-CP-C-A-CP-C-A-nMT-CP)	non-ribosomal peptide synthase/polyketide synthase (*Methylocaldum marinum*)	52.86%	WP_269461530.1
DscD	5428	NRPS (domains: C-A-E-CP-C-A-CP-C-A-CP-C-A-nMT-CP-TE)	non-ribosomal peptide synthetase (*Scytonema hofmannii*)	53.88%	WP_100898072.1
DscE	693	rSAM methyltransferase (SAM-dependent and B12-dependent)	RiPP maturation radical SAM *C*-methyltransferase (*Azospirillum fermentarium*)	52.05%	MCL4542996.1
ORF1	74	MbtH-like protein	MbtH family protein (*Stigmatella erecta*)	90.77%	HEY0079189.1

Supplementary Table 10: Nucleotide sequences of genes encoding Cmb that were codon-optimized for *E. coli* as used in this work and amino acid sequences of the resulting proteins.

	Cmb^{Es}	Cmb^{Em}
Amino acid sequence	MSLHHSIQSRLYCPIPSAINPLVDTIHQQTLQWAC<br>DFKLVVTDGKTYPRLSAAKFAWLVARGYPEVNLH<br>NLQIVSDWITCVFIHDDLCDGSRVGKTPQGLRPM<br>HHQFLLALDGEHGHALDDPLSYALRDIYQRLREKT<br>SDGWRRRFRDHMAAYFQANRWEAQNRLLDRIPS<br>LDTYLHWRQYTSCVEPCFDLILMSDQLSSEAAYL<br>SHESVRSLARMANNYIAWVNDIFSLNRERQEGNM<br>NNLVVVLQNEYQLPLQAALELAIEMCNSEMEAFL<br>NLASHLPSWGEQEDDQLNRYIRGFRSWMRGNLD<br>WYMDTGRYHVEDERSEIDTSDMDMLALAS	MAVTTIPYGDRTTNHRPTQPSIYCPVSSSINRHVETI<br>HHRTLQWACDFNLVLPGTPDYQRLEAAKFAWLVAR<br>VYPRAGYTELQIISDWVTWIFIHDDLCDVSAIGKRPQ<br>RLRHIHNMMLWVLEGGSLTAENQRLASALYDIRQRL<br>WWQTDAQWLRRFTDHMDQYFQANRWEASNREA<br>SRTPSLEAYTEMRPFTSGLEPCIDLMLMAAQLSAAS<br>AFLKHRVVHSLTLRANRFISWTNDIYGLDKEIQEGN<br>MNNLVLVLQHEYQLSAQAAIHLAVEMCNTEMEAFLS<br>LESNLPSFGEAEDIKLNTYLAGLRSWMRGTLDWYL<br>ESGRYDVETGVRLSVPARVETHALCA
Original nucleotide sequence	ATGAGCTTACACCACTCTATCCAATCTCGATTATA<br>TTGCCCGATCCCTTCTGCGATCAATCCACTTGT<br>CGATACCATTCACCAGCAAACCTTGCAGTGGGC<br>CTGTGATTTCAAGCTAGTGGTGACCGACGGCAA<br>GACTTATCCGCGTCTGAGTGCCGCCAAATTCGC<br>CTGGTTAGTGGCACGCGGATATCCCGAAGTGAA<br>CCTTCACAATCTACAAATTGTTTCTGATTGGATC<br>ACTTGTGTCTTTATACATGACGACTTATGTGACG<br>GTTCTAGGGTTGGAAAAACGCCACAAGGTTTAC<br>GGCCTATGCATCACCAATTTTTGTTGGCATTAGA<br>TGGGGAGCATGGTCATGCTCTAGACGATCCGTT<br>GAGCTACGCGTTGCGTGATATCTATCAACGGTT<br>GCGGGAGAAGACGAGCGATGGCTGGCGGCGA<br>CGATTTCGGGATCACATGGCCGCCTATTTCCAA<br>GCTAACCGTTGGGAGGCCCAGAATCGACTTCTT<br>GATCGTATCCCTAGCCTCGACACGTATCTCCACT<br>GGCGTCAATACACAAGTTGTGTTGAGCCATGTT<br>TTGATCTCATTCTGATGAGTGACCAACTCTCATC<br>AGAAGCGGCTTATCTCAGCCATGAGAGCGTGA<br>GATCATTGGCCAGGATGGCGAACAATTATATCGC<br>TTGGGTCAATGATATTTTTAGCCTCAACCGGGAG<br>CGTCAAGAAGGCAATATGAATAATCTCGTTGTCG<br>TCTTACAAAACGAGTACCAGTTGCCCCTCCAGG<br>CAGCCCTCGAGCTGGCCATTGAGATGTGTAATA<br>GTGAAATGGAAGCATTTCTCAACTTAGCCTCTCA<br>TCTGCCTTCATGGGGCGAGCAAGAAGATGACC<br>AGCTCAACCGATATATCCGGGGTTTTCGATCCT<br>GGATGCGTGGCAACTTGGATTGGTATATGGATA<br>CCGGGCGCTACCACGTCGAGGATGAGCGCTCC<br>GAAATAGACACGAGTGACATGGACATGCTTGCT<br>CTGGCGTCTTAG	ATGGCCGTGACCACAATTCCGTATGGCGATCGTA<br>CGACGAACCATCGTCCAACCCAACCGAGCATTTA<br>TTGTCCCGTTTCTTCCAGCATCAATCGACATGTCG<br>AAACCATTCATCACCGGACCTTGCAGTGGGCATG<br>TGATTTTAATCTTGTGTTGCCTGGCACCCCAGACT<br>ACCAGCGTTTAGAGGCAGCAAAATTCGCCTGGCT<br>GGTGGCACGTGTTTATCCTCGGGCTGGGTATACC<br>GAACTGCAGATCATCTCGGATTGGGTCACTTGGA<br>TTTTTATTCATGACGATTTATGCGACGTGTCGGCG<br>ATTGGAAAGCGCCCACAGCGATTGCGACACATTC<br>ACAACATGATGCTTTGGGTGCTGGAAGGTGGTAG<br>CTTGACGGCTGAAAATCAGCGGCTCGCCTCTGCA<br>TTGTACGATATTCGTCAACGGCTATGGTGGCAAAC<br>GGATGCGCAATGGCTGCGGCGCTTTACCGACCAT<br>ATGGACCAATATTTCCAGGCCAATCGATGGGAGG<br>CCAGCAATCGAGAGGCCAGTCGGACCCCTTCTCT<br>CGAGGCCTATACCGAGATGCGCCCCTTCACGAGT<br>GGCTTAGAGCCCTGTATTGATCTGATGCTCATGGC<br>GGCACAGCTCTCTGCTGCATCAGCGTTTTTGAAA<br>CACCGCGTCGTCCATTCGCTGACCTTGCGGGCA<br>AACCGTTTTATCTCCTGGACCAATGATATCTATGGT<br>CTCGACAAAGAAATTCAAGAGGGCAACATGAACA<br>ACCTCGTGCTGGTGCTACAGCACGAGTATCAGCT<br>TTCAGCTCAAGCGGCCATACATCTTGCGGTTGAG<br>ATGTGCAATACGGAAATGGAAGCCTTTCTCAGCCT<br>AGAATCGAATCTCCCTTCGTTCGGGGAGGCTGAA<br>GATATCAAGCTCAACACGTACCTTGCCGGGCTGC<br>GGTCGTGGATGCGCGGCACCTTGGATTGGTATCT<br>GGAGTCAGGCCGGTACGACGTTGAGACGGGCGT<br>GCGTCTTAGTGTTCCTGCACGCGTTGAAACCCAC<br>GCCTTGTGCGCTTAA
Optimized nucleotide sequence	ATGAGTTTGCACCACAGCATCCAAAGCCGCCTG<br>TACTGTCCGATTCCGTCCGCTATTAACCCACTG<br>GTCGATACGATTCATCAGCAAACGTTGCAGTGG<br>GCGTGCGACTTTAAGCTGGTAGTTACAGATGGC	ATGGCCGTCACGACCATTCCGTATGGGGACCGTA<br>CCACAAATCACCGTCCTACACAGCCGAGCATCTAT<br>TGCCCCGTAAGTTCGAGCATTAACCGTCATGTTGA<br>GACTATTCATCATCGTACCTTACAGTGGGCTTGTG

	AAGACGTATCCACGCTTGAGCGCAGCGAAGTTT<br>GCTTGGTTAGTAGCCCGTGGATACCCAGAAGTT<br>AACTTGCACAACCTTCAAATCGTGTCTGACTGG<br>ATTACTTGCGTGTTCATCCACGATGACCTCTGTG<br>ACGGGTCACGCGTGGGCAAAACCCCTCAGGGT<br>CTCCGTCCCATGCATCATCAATTTCTGCTGGCTC<br>TCGATGGGGAGCACGGTCACGCACTTGACGAC<br>CCACTTTCTTATGCGTTACGCGATATTTATCAGC<br>GCTTACGTGAGAAGACGTCAGACGGTTGGCGT<br>CGCCGTTTCCGTGACCACATGGCGGCGTACTT<br>CCAAGCGAATCGTTGGGAAGCCCAAAATCGCTT<br>GCTTGATCGCATCCCCTCACTGGACACGTATCT<br>CCACTGGCGCCAATACACCAGTTGCGTGGAAC<br>CCTGTTTCGATCTGATCCTGATGTCTGACCAGC<br>TCAGCAGCGAGGCAGCGTACCTCTCACATGAG<br>TCAGTTCGCTCACTTGCGCGTATGGCAAACAAC<br>TATATCGCCTGGGTGAACGATATCTTCTCGTTAA<br>ATCGCGAGCGCCAAGAGGGGAATATGAACAATC<br>TTGTGGTCGTGCTCCAAAATGAGTATCAATTGCC<br>GCTGCAAGCCGCGCTTGAACTCGCGATCGAGA<br>TGTGTAATAGTGAGATGGAAGCGTTCTTAAATCT<br>CGCATCGCACCTTCCTAGTTGGGGTGAACAAGA<br>GGATGACCAGCTTAATCGCTATATTCGCGGATTT<br>CGCTCCTGGATGCGCGGGAACCTTGACTGGTA<br>CATGGATACAGGGCGTTACCATGTGGAAGACGA<br>GCGTAGTGAGATTGATACGTCAGACATGGATATG<br>CTGGCCCTCGCATCGTGA	ACTTTAATCTGGTTCTGCCAGGGACCCCGGATTAT<br>CAACGTCTTGAGGCTGCGAAGTTCGCATGGTTGG<br>TAGCACGTGTGTACCCCCGTGCGGGATATACGGA<br>GCTTCAAATCATCTCCGATTGGGTCACTTGGATCT<br>TTATCCATGACGACCTGTGTGACGTGTCGGCGAT<br>CGGGAAGCGTCCTCAACGTTTGCGCCATATCCAC<br>AATATGATGCTGTGGGTTCTCGAAGGTGGTTCGTT<br>AACGGCCGAGAATCAGCGCTTGGCCAGCGCGCT<br>GTACGACATCCGCCAACGCCTTTGGTGGCAAACC<br>GATGCCCAGTGGTTGCGTCGTTTTACTGATCACAT<br>GGACCAATATTTTCAAGCCAACCGCTGGGAAGCG<br>TCGAACCGCGAGGCGAGCCGTACGCCCTCACTT<br>GAGGCTTATACGGAAATGCGCCCTTTTACGAGCG<br>GGCTGGAGCCTTGTATCGATTTGATGCTCATGGC<br>AGCTCAGTTATCTGCCGCAAGTGCCTTCTTAAAGC<br>ATCGTGTCGTTCACAGCCTCACGCTTCGTGCAAA<br>CCGTTTTATCTCGTGGACCAATGACATTTATGGTC<br>TTGACAAGGAGATCCAAGAAGGTAATATGAATAAC<br>TTGGTTCTCGTGCTGCAACATGAGTATCAGCTGA<br>GTGCGCAGGCAGCCATCCACTTAGCAGTAGAAAT<br>GTGCAACACTGAGATGGAAGCGTTTCTGTCGTTG<br>GAGTCGAACCTGCCCTCTTTCGGCGAGGCAGAG<br>GACATCAAGCTGAATACGTACTTGGCCGGACTTC<br>GTAGTTGGATGCGCGGTACGCTTGACTGGTACTT<br>GGAAAGTGGCCGTTACGACGTAGAGACCGGCGT<br>GCGCTTGTCTGTTCCTGCCCGTGTCGAGACGCAT<br>GCGCTGTGCGCATAA

Supplementary Table 11: Protein list for sequences used to construct the phylogenetic tree of Cmb-related terpene synthases in Fig. 5D.

Accession number
A0A291SJC7.1
A9FZ87.1
ABG03467.1
ACC41646.1
ACU72436.1
AMP04969.1
B2J4A4.17
BAB76384.1
BAC67785.1
BAJ25873.1
BAJ27126.1
BAL14866.1
BAL14867.1
BAM 78697.00
BAM 78698.10
BAP82203.1
BAP82213.1
BAP82216.1
BAP82229.1
CBG74359.1
A0A291SJC7.1
A9FZ87.1
ABG03467.1
ACC41646.1
ACU72436.1
AMP04969.1
B2J4A4.17
BAB76384.1
BAC67785.1
BAJ25873.1

Accession number
BAJ27126.1
BAL14866.1
BAL14867.1
BAM 78697.00
BAM 78698.10
BAP82203.1
BAP82213.1
BAP82216.1
BAP82229.1
CBG74359.1
CCA53839.1
COTB2_STRMJ
D2B747.1
E8W6C7.1
EDY47508.1
EDY49122.1
EDY49701.1
EDY50541.1
EDY62784.1
EFG04252.1
EFG04472.1
EFG04655.1
EFG04671.1
EFG04889.1
EFL30447.1
EFL36708.1
I2N045.1
K0K750.1
P0DPK6.1
Q9K499.1

Accession number
Q55012.4
QGF19026.1
SAVERM_2998
SAVERM_3032
SEW49220.1
SGR2079
SGR6065
SHK33953.1
SHL60838.1
WP_004941320.1
WP_005317515.1
WP_010314578.1
WP_012119179.1
WP_012789469.1
WP_012792334.1
WP_014150548.1
WP_020663213.1
WP_030426588.1
WP_030430753.1
WP_030431358.1
WP_030432512.1
WP_039931950.1
WP_051165600.1
WP_051714436.1
WP_061917807
WP_061922997
WP_067429395.1
WP_091045378.1
WP_091046421

Supplementary Table 12: List of genes and their functions used to generate the tree in Supplementary Fig. 2 by running the autoMLST locally.

NCBI HMM accession	Gene name	Gene function
TIGR00135	*gatC*	Protein synthesis
TIGR00445	*mraY*	Cell envelope
TIGR01574	*miaB-methiolase*	Protein synthesis
TIGR02273	*16S_RimM*	Transcription
TIGR00002	*S16*	Protein synthesis
TIGR02225	*recomb_XerD*	DNA metabolism
TIGR00165	*S18*	Protein synthesis
TIGR00166	*S6*	Protein synthesis
TIGR00713	*hemL*	Biosynthesis of cofactors, prosthetic groups, and carriers
TIGR00496	*frr*	Protein synthesis
TIGR00492	*alr*	Cell envelope
TIGR03534	*RF_mod_PrmC*	Protein fate
TIGR01128	*holA*	DNA metabolism
Ribosomal_S9	*PF00380.15*	Unclassified
TIGR00012	*L29*	Protein synthesis
TIGR00150	*T6A_YjeE*	Protein synthesis
TIGR01164	*rplP_bact*	Protein synthesis
TIGR03654	*L6_bact*	Protein synthesis
TIGR00114	*lumazine-synth*	Biosynthesis of cofactors, prosthetic groups, and carriers
TIGR00020	*prfB*	Protein synthesis
TIGR00187	*ribE*	Biosynthesis of cofactors, prosthetic groups, and carriers
TIGR00233	*trpS*	Protein synthesis
TIGR00337	*PyrG*	Purines, pyrimidines, nucleosides, and nucleotides
TIGR00331	*hrcA*	Regulatory functions
TIGR00962	*atpA*	Energy metabolism
TIGR00967	*3a0501s007*	Protein fate
TIGR00061	*L21*	Protein synthesis
TIGR00060	*L18_bact*	Protein synthesis
TIGR00062	*L27*	Protein synthesis
Ribosomal_L5	*PF00281.15*	Unclassified
TIGR01174	*ftsA*	Cellular processes
TIGR01071	*rplO_bact*	Protein synthesis

TIGR00952	*S15_bact*	Protein synthesis
TIGR01079	*rplX_bact*	Protein synthesis
TIGR00512	*salvage_mtnA*	Amino acid biosynthesis
TIGR00517	*acyl_carrier*	Fatty acid and phospholipid metabolism
TIGR00105	*L31*	Protein synthesis
TIGR00473	*pssA*	Fatty acid and phospholipid metabolism
TIGR01951	*nusB*	Transcription
TIGR00036	*dapB*	Amino acid biosynthesis
TIGR00326	*eubact_ribD*	Biosynthesis of cofactors, prosthetic groups, and carriers
TIGR00088	*trmD*	Protein synthesis
TIGR00086	*smpB*	Protein synthesis
TIGR00082	*rbfA*	Transcription
TIGR01021	*rpsE_bact*	Protein synthesis
TIGR01024	*rplS_bact*	Protein synthesis
TIGR00431	*TruB*	Protein synthesis
TIGR01853	*lipid_A_lpxD*	Cell envelope
TIGR01852	*lipid_A_lpxA*	Cell envelope
TIGR00487	*IF-2*	Protein synthesis
TIGR03635	*uS17_bact*	Protein synthesis
TIGR01066	*rplM_bact*	Protein synthesis
TIGR01060	*eno*	Energy metabolism

Supplementary Table 13: Primers used in this study.

Primer name	Sequence
Cmb^{Es}-F	5’-GCCATATGAGTTTGCACCACAGCATCCAAA-3’
Cmb^{Es}-R	5’-GGAAGCTTTCACGATGCGAGGGCCAGCAT-3’
Cmb^{Em}-F	5’-GCCATATGGCCGTGACCACAATTCCGTATG-3’
Cmb^{Em}-R	5’-GGAAGCTTTTAAGCGCACAAGGCGTGGGT-3’

Supplementary References

1. Alanjary, M., Steinke, K. & Ziemert, N. AutoMLST: an automated web server for generating multi-locus species trees highlighting natural product potential. *Nucleic Acids Res.* **47**, W276–W282 (2019).
2. Parks, D.H. et al. GTDB: an ongoing census of bacterial and archaeal diversity through a phylogenetically consistent, rank normalized and complete genome-based taxonomy. *Nucleic Acids Res.* **50**, D785–D794 (2022).
3. Jain, C., Rodriguez, R.L., Phillippy, A.M., Konstantinidis, K.T. & Aluru, S. High throughput ANI analysis of 90K prokaryotic genomes reveals clear species boundaries. *Nat. Commun.* **9**, 5114 (2018).
4. Nei, M. & Kumar, S. *Molecular Evolution and Phylogenetics*, (Oxford University Press, 2000).
5. Saitou, N. & Nei, M. The Neighbor-joining Method: a new method for reconstructing phylogenetic trees. *Mol. Biol. Evol.* **4**, 406–425 (1987).
6. Felsenstein, J. Confidence limits on phylogenies: an approach using the bootstrap. *Evolution* **39**, 783–791 (1985).
7. Kumar, S., Stecher, G. & Tamura, K. MEGA7: molecular evolutionary genetics analysis version 7.0 for bigger datasets. *Mol. Biol. Evol.* **33**, 1870–1874 (2016).
8. Gilchrist, C.L.M. & Chooi, Y.H. clinker & clustermap.js: automatic generation of gene cluster comparison figures. *Bioinformatics* **37**, 2473–2475 (2021).
9. Lackner, G., Peters, E.E., Helfrich, E.J. & Piel, J. Insights into the lifestyle of uncultured bacterial natural product factories associated with marine sponges. *Proc. Natl. Acad. Sci. USA* **114**, E347–E356 (2017).
10. Reiter, S., Cahn, J.K.B., Wiebach, V., Ueoka, R. & Piel, J. Characterization of an orphan type III polyketide synthase conserved in uncultivated "Entotheonella" sponge symbionts. *ChemBioChem* **21**, 564–571 (2020).
11. Wilson, M.C. et al. An environmental bacterial taxon with a large and distinct metabolic repertoire. *Nature* **506**, 58–62 (2014).
12. Ueoka, R. et al. Metabolic and evolutionary origin of actin-binding polyketides from diverse organisms. *Nat. Chem. Biol.* **11**, 705–712 (2015).
13. Chen, K.T., Shen, H.T. & Lu, C.L. Multi-CSAR: a multiple reference-based contig scaffolder using algebraic rearrangements. *BMC Syst. Biol.* **12**, 139 (2018).
14. Parks, D.H., Imelfort, M., Skennerton, C.T., Hugenholtz, P. & Tyson, G.W. CheckM: assessing the quality of microbial genomes recovered from isolates, single cells, and metagenomes. *Genome Res.* **25**, 1043–1055 (2015).
15. Blin, K. et al. antiSMASH 7.0: new and improved predictions for detection, regulation, chemical structures and visualisation. *Nucleic Acids Res.* **51**, W46–W50 (2023).
16. Freeman, M.F. et al. Metagenome mining reveals polytheonamides as posttranslationally modified ribosomal peptides. *Science* **338**, 387–390 (2012).
17. Dmitrijeva, M. et al. The mOTUs online database provides web-accessible genomic context to taxonomic profiling of microbial communities. *Nucleic Acids Res.* **53**, D797–D805 (2024).
