# Supplementary material for: Chemical richness and diversity of uncultivated ‘Entotheonella’ symbionts in marine sponges
Source: Nat Chem Biol. 2025 Nov 13;22(2):217–28. doi: 10.1038/s41589-025-02066-0 (PMC12858407; doi:10.1038/s41589-025-02066-0)
Supplement: Supplementary file 2 — Reporting Summary [file 41589_2025_2066_MOESM2_ESM.pdf]

nature portfolio

Corresponding author(s): Jörn Piel, Haruko Takeyama, Toshiyuki Wakimoto

Last updated by author(s): Sep 19, 2024

Reporting Summary

Nature Portfolio wishes to improve the reproducibility of the work that we publish. This form provides structure for consistency and transparency in reporting. For further information on Nature Portfolio policies, see our Editorial Policies and the Editorial Policy Checklist.

Statistics

For all statistical analyses, confirm that the following items are present in the figure legend, table legend, main text, or Methods section.

n/a	Confirmed	
☐	☒	The exact sample size (*n*) for each experimental group/condition, given as a discrete number and unit of measurement
☐	☒	A statement on whether measurements were taken from distinct samples or whether the same sample was measured repeatedly
☒	☐	The statistical test(s) used AND whether they are one- or two-sided<br>*Only common tests should be described solely by name; describe more complex techniques in the Methods section.*
☒	☐	A description of all covariates tested
☐	☒	A description of any assumptions or corrections, such as tests of normality and adjustment for multiple comparisons
☒	☐	A full description of the statistical parameters including central tendency (e.g. means) or other basic estimates (e.g. regression coefficient) AND variation (e.g. standard deviation) or associated estimates of uncertainty (e.g. confidence intervals)
☒	☐	For null hypothesis testing, the test statistic (e.g. *F*, *t*, *r*) with confidence intervals, effect sizes, degrees of freedom and *P* value noted<br>*Give P values as exact values whenever suitable.*
☒	☐	For Bayesian analysis, information on the choice of priors and Markov chain Monte Carlo settings
☒	☐	For hierarchical and complex designs, identification of the appropriate level for tests and full reporting of outcomes
☒	☐	Estimates of effect sizes (e.g. Cohen's *d*, Pearson's *r*), indicating how they were calculated

Our web collection on statistics for biologists contains articles on many of the points above.

Software and code

Policy information about availability of computer code

Data collection	Sequencing techniques: Illumina HiSeq2500 system and Illumina Novaseq600 platform (Illumina), MinION Mk1C (Oxford Nanopore Technologies), MiSeq (Illumina); MS acquisition: Xcalibur 4.1, Chromeleon Xpress 7.2 (Thermo Fisher), HPLC: Open Lab CDS 2.2 (Agilent), NMR acquisition: Delta v5.3 (JEOL, Japan), GC-MS acquisition: LabSolutions CS (v.4.42) (Shimadzu)
Data analysis	DNA assemblies and binning: BBDuk (v37.55, Joint Genome Institute), BBNorm (v37.55, Joint Genome Institute) (BBMap download | SourceForge.net), metaSPAdes (v3.11.0 and v3.12) (https://doi.org/10.1101/gr.213959.116), SAMtools (v1.9), (https://doi.org/10.1093/gigascience/giab008), BWA (v0.7.1 and v0.7.17), (Burrows-Wheeler Aligner (sourceforge.net)), MetaBAT2 (v2.12.1 and 2.15), (https://doi.org/10.7717/peerj.7359), metaSPAdes (v3.12) (doi:10.1101/gr.213959.116), fastp (v0.20.0) (https://doi.org/10.1093/bioinformatics/bty560), Flye (v2.8.1-b1676), (https://doi.org/doi:10.1038/s41592-020-00971-x), Canu (v.1.4) (https://doi.org/10.1101/gr.215087.116), Pilon (v.1.22) (https://doi.org/10.1371/journal.pone.0112963), MetaWRAP v1.2, (https://doi.org/10.1186/s40168-018-0541-1), SPAdes 3.12.0, (https://doi.org/10.1002/cpbi.102), CheckM v1.0.6 and v1.0.13 (10.1101/gr.186072.114), QUAST (v4.5), (QUAST (sourceforge.net), Kaiju (v.1.6.2) (https://doi.org/10.1038/ncomms11257), Prodigal (v2.6.3) (https://doi.org/10.1186/1471-2105-11-119), Multi-CSAR (https://doi.org/10.1186/s12918-018-0654-y), autoMLST (https://doi.org/10.1093/nar/gkz282), FastANI (https://doi.org/10.1038/s41467-018-07641-9), RASTtK (https://doi.org/10.1038/srep08365), GTDB-Tk (https://doi.org/10.1093/nar/gkab776), Geneious v.8.1.9 (Biomatters Limited), MEGA7 (https://doi.org/10.1093/molbev/msw054), antiSMASH7 (https://doi.org/10.1093/nar/gkad344), BiG-SCAPE (https://doi.org/10.1038/s41589-019-0400-9), Cytoscape 3 (https://doi.org/10.1101/gr.1239303.), HPLC-HRMS: Xcalibur 4.1 (Thermo Fisher); NMR Topspin 4.1 (Bruker), GC-MS:LabSolutions CS (v4.42) (Shimadzu), NMR: Delta v5.3 (JEOL, Japan)

For manuscripts utilizing custom algorithms or software that are central to the research but not yet described in published literature, software must be made available to editors and reviewers. We strongly encourage code deposition in a community repository (e.g. GitHub). See the Nature Portfolio guidelines for submitting code & software for further information.

Data

Policy information about availability of data

All manuscripts must include a data availability statement. This statement should provide the following information, where applicable:

- Accession codes, unique identifiers, or web links for publicly available datasets
- A description of any restrictions on data availability
- For clinical datasets or third party data, please ensure that the statement adheres to our policy

All data supporting the findings of this study are available within the main text and the supplemental information. DNA sequences are deposited at the European Nucleotide Archive (ENA) under the BioProject numbers PRJEB80215 (all except for 'Ca. P. opulenta' AC1) and PRJEB59408 ('Ca. P. opulenta' AC1), and accession numbers for assemblies are as following:

'Ca. E. symbiotica' BT01: GCA_964656635, 'Ca. E. inquilina' BT02: GCA_964656685, 'Ca. E. melakyensis' BT03: GCA_964656765, 'Ca. E. catenata' BT04: GCA_964656715, 'Ca. E. armillaria' DC1: GCA_964656755, 'Ca. E. tacita' DD1: GCA_964656785, 'Ca. E. baccata' DD2: GCA_964656735, 'Ca. E. tertia' DD3: GCA_964656775, 'Ca. E. monilis' DK1: GCA_964656725, 'Ca. E. melakyensis' TCBA1: GCA_964656645, 'Ca. E. serta' TCBA2: GCA_964656625, 'Ca. E. serta' TSWA1: GCA_964656745, 'Ca. E. serta' TSWB1: GCA_964656705, 'Ca. E. consors' TSWB2: GCA_964656675, 'Ca. E. factor' TSYB1: GCA_964656695, 'Ca. E. gemina' TSYB2: GCA_964656665, 'Ca. E. mitsugo' TSYB3: GCA_964656655, 'Ca. P. opulenta' AC1: ERZ25059164.

The dsc BGC is deposited on MIBiG with the following accession number BGC0003182.

Other data related to this work (e.g. HPLC-HRMS) are available from the lead contact upon request because they might include data for ongoing projects that will be processed at a later stage of this project.

Human research participants

Policy information about studies involving human research participants and Sex and Gender in Research.

Reporting on sex and gender	N/A
Population characteristics	N/A
Recruitment	N/A
Ethics oversight	N/A

Note that full information on the approval of the study protocol must also be provided in the manuscript.

Field-specific reporting

Please select the one below that is the best fit for your research. If you are not sure, read the appropriate sections before making your selection.

☒ Life sciences ☐ Behavioural & social sciences ☐ Ecological, evolutionary & environmental sciences

For a reference copy of the document with all sections, see nature.com/documents/nr-reporting-summary-flat.pdf

Life sciences study design

All studies must disclose on these points even when the disclosure is negative.

Sample size	The sample size (number) of analysed sponges was determined by the occurence of 'Ca. Entotheonella' phylotypes and the availability of sponge samples. Sampling sponges is not an easy and cheap thing to do. This is why we had usually only one sponge sample per specimen.
Data exclusions	No data were excluded from this study.
Replication	All protein expressions were repeated multiple times, with consistent outcomes that were measured. Enzyme assays were performed with freshly purified enzyme and in triplicates and were all successful and had the same outcome.
Randomization	No randomization of samples was necessary to this study because we looked at specific samples and wanted to link sponge metabolites to their respective genomic origin. We needed to know what the respecitve sample contains.
Blinding	Blinding was not necessary to this study, because we looked at the specific samples and wanted to link sponge metabolites to their respeictiv genomic origin. We needed to know what the respecitve sample contains.

Reporting for specific materials, systems and methods

We require information from authors about some types of materials, experimental systems and methods used in many studies. Here, indicate whether each material, system or method listed is relevant to your study. If you are not sure if a list item applies to your research, read the appropriate section before selecting a response.

Materials & experimental systems

n/a	Involved in the study
☒	☐ Antibodies
☒	☐ Eukaryotic cell lines
☒	☐ Palaeontology and archaeology
☒	☐ Animals and other organisms
☒	☐ Clinical data
☒	☐ Dual use research of concern

Methods

n/a	Involved in the study
☒	☐ ChIP-seq
☒	☐ Flow cytometry
☒	☐ MRI-based neuroimaging
